# Efficacy and tolerability of an endogenous metabolic modulator (AXA1125) in fatigue-predominant long COVID: a single-centre, double-blind, randomised controlled phase 2a pilot study

**DOI:** 10.1016/j.eclinm.2023.101946

**Published:** 2023-04-14

**Authors:** Lucy E.M. Finnigan, Mark Philip Cassar, Margaret James Koziel, Joel Pradines, Hanan Lamlum, Karim Azer, Dan Kirby, Hugh Montgomery, Stefan Neubauer, Ladislav Valkovič, Betty Raman

**Affiliations:** a Oxford Centre for Clinical Magnetic Resonance Research (OCMR), Division of Cardiovascular Medicine, Radcliffe Department of Medicine, University of Oxford, John Radcliffe Hospital, Oxford, UK; b Axcella Therapeutics, Cambridge, MA, USA; c University College London, London, UK; d Oxford NIHR Biomedical Research Centre, Oxford University Hospitals Foundation Trust, Oxford, UK; e Institute of Measurement Science, Slovak Academy of Sciences, Bratislava, Slovakia

**Keywords:** Long COVID, Mitochondrial dysfunction, Fatigue

## Abstract

**Background:**

‘Long COVID’ describes persistent symptoms, commonly fatigue, lasting beyond 12 weeks following SARS-CoV-2 infection. Potential causes include reduced mitochondrial function and cellular bioenergetics. AXA1125 has previously increased β-oxidation and improved bioenergetics in preclinical models along with certain clinical conditions, and therefore may reduce fatigue associated with Long COVID. We aimed to assess the efficacy, safety and tolerability of AXA1125 in Long COVID.

**Methods:**

Patients with fatigue-dominant Long COVID were recruited in this single-centre, double-blind, randomised controlled phase 2a pilot study completed in the UK. Patients were randomly assigned (1:1) using an Interactive Response Technology to receive either AXA1125 or matching placebo in a clinical-based setting. Each dose (33.9 g) of AXA1125 or placebo was administered orally in a liquid suspension twice daily for four weeks with a two-week follow-up period. The primary endpoint was the mean change from baseline to day 28 in the phosphocreatine (PCr) recovery rate following moderate exercise, assessed by ^31^P-magnetic resonance spectroscopy (MRS). All patients were included in the intention to treat analysis. This trial was registered at ClinicalTrials.gov, NCT05152849.

**Findings:**

Between December 15th 2021, and May 23th 2022, 60 participants were screened, and 41 participants were randomised and included in the final analysis. Changes in skeletal muscle phosphocreatine recovery time constant (τ_PCr_) and 6-min walk test (6MWT) did not significantly differ between treatment (n = 21) and placebo group (n = 20). However, treatment with AXA1125 was associated with significantly reduced day 28 Chalder Fatigue Questionnaire [CFQ-11] fatigue score when compared with placebo (least squares mean difference [LSMD] −4.30, 95% confidence interval (95% CI) −7.14, −1.47; *P* = 0.0039). Eleven (52.4%, AXA1125) and four (20.0%, placebo) patients reported treatment-emergent adverse events; none were serious or led to treatment discontinuation.

**Interpretation:**

Although treatment with AXA1125 did not improve the primary endpoint (τ_PCr_-measure of mitochondrial respiration), when compared to placebo, there were significant improvements in fatigue-based symptoms among patients living with Long COVID following a four-week treatment period. Further multicentre studies are needed to validate our findings in a larger cohort of patients with fatigue-dominant Long COVID.

**Funding:**

Axcella Therapeutics.

**Research in context**

**Evidence before this study**

We conducted a search from inception of our study up to March 2023 (PubMed, Scopus Web of Science, Cochrane, MEDLINE, EMBASE, CINAHL). Search terms included but were not limited to “Long COVID”, “metabolic”, “double-blind” and “placebo”. There was only one complete peer-reviewed study which evaluated the role of l-Arginine and vitamin C supplementation in Long COVID and showed some benefit of nutritional supplementation. All other studies were either interim reports or protocol design papers.

Long COVID remains a poorly understood condition with no approved disease-specific therapies. Prior studies have suggested a role of SARS-CoV-2 in disrupting mitochondrial homeostasis, which is centrally important for numerous cellular processes. Mitochondrial dysfunction has been implicated in other conditions like chronic fatigue syndrome that manifest with symptoms of fatigue. In this study we evaluated the efficacy of AXA1125, an endogenous metabolic modulator, on a measure of mitochondrial health and symptoms of physical and mental fatigue among patients as assessed by a validated questionnaire.

**Added value of this study**

This is the first double-blind placebo-controlled trial of a metabolic modulator in Long COVID. We showed that AXA1125 was associated with significant improvements in physical and mental fatigue symptoms following 4 weeks of oral therapy. While there was no improvement in the measure of mitochondrial oxidative capacity by treatment allocation, the high baseline values of this measure support the role of mitochondrial dysfunction as a potential pathophysiologic mechanism in Long COVID.

**Implications of all the available evidence**

This phase 2a proof of concept study demonstrated that treatment with AXA1125 did not improve phosphocreatine recovery rate time constant following exercise, a measure of mitochondrial respiration. However, AXA1125 did significantly improve symptoms of physical and cognitive fatigue experienced by patients suffering from Long COVID, which was not seen in the placebo arm. These findings underscore the need for further efforts to evaluate the efficacy of AXA1125 on patients with Long COVID symptoms in a larger phase 3 multicentre clinical trial.

## Introduction

By September 2022, over 607 million confirmed COVID-19 cases were reported worldwide.^1^ Many of these patients have suffered ongoing symptoms beyond 12 weeks (defined as ‘post-COVID-19 syndrome’ or ‘Long COVID'), placing a significant burden on healthcare systems globally. Fatigue affects 37%–53% of patients with Long COVID.^2^^,^^3^ Effective treatments have yet to be identified, largely because the pathogenesis remains unclear. While vascular inflammation, immune dysregulation, altered gut microbiome and sustained viral replication may play a role in some cases, mounting evidence suggests a role for mitochondrial dysfunction and impaired cellular bioenergetics.^4^, ^5^, ^6^ The SARS-CoV-2 virus is thought to ‘hijack’ the host mitochondria to facilitate viral replication, compromising function, increasing inflammation and oxidative stress.^7^ Subsequent energy dysregulation triggered by metabolic reprogramming (a switch from oxidative phosphorylation to glycolysis) is proposed to manifest as chronic fatigue.^7^ Consistent with this hypothesis, low levels of several metabolites and impaired energy production from all cellular sources (including amino acids) have also been reported in patients with chronic fatigue syndrome, a closely related condition.^8^

Amino acids are not only the building blocks of proteins but are also metabolic modulators. AXA1125, an orally administered endogenous metabolic modulator (EMM) comprised of five amino acids (leucine, isoleucine, valine, arginine, glutamine) and N-acetylcysteine, which increases β-oxidation (which provides fuel substrate for oxidative phosphorylation, a more efficient source of energy than glycolysis when oxygen is not limiting) leading to improved mitochondrial function, and decreased oxidative stress in a preclinical model of non-alcoholic steatohepatitis (NASH).^9^ Administration of AXA1125 to patients with non-alcoholic fatty liver disease resulted in the reduction of liver fat volume markers and inflammation.^10^ These findings suggest that AXA1125 might offer therapeutic benefits in patients with Long COVID. The aims of this phase 2a study were to assess the efficacy, safety, and tolerability of AXA1125 in patients with fatigue-predominant Long COVID.

## Methods

### Study design

This randomised, double blind, placebo-controlled (1:1 (treatment : placebo) allocation), single centre (University of Oxford) study (ClinicalTrials.gov identifier: NCT05152849) was conducted in accordance with the Good Clinical Practice guideline (International Conference on Harmonisation of Technical Requirements for Registration of Pharmaceuticals for Human Use [ICH] E6), and applicable local regulations governing clinical research. The study was approved by the Health Research Authority Fast Track research ethics committee (REC) (REC Reference: 21/FT/0158) and the United Kingdom Medicines and Healthcare Products Regulatory Authority (MHRA) (reference number: CTA 54043/0003/001-0001). All participants provided written informed consent prior to study entry. There were no changes to methods following trial commencement. Data was presented in adherence to CONSORT guidelines for reporting randomised clinical trials.

### Patients

Eligible for study inclusion were individuals (i) 18–64 years of age, (ii) with clinically suspected COVID-19 ≥ 12 weeks prior to screening, (iii) displayed fatigue predominant Long COVID, as defined by a total fatigue (bimodal) score of ≥8 on the Chalder Fatigue Questionnaire [CFQ-11]), and (iv) with a post-exertional skeletal muscle phosphocreatine recovery rate constant [τ_PCr_] >50 s (a marker of impaired mitochondrial oxidative capacity) measured using phosphorus magnetic resonance spectroscopy (^31^P-MRS). Patients presenting with other possible causes of fatigue (e.g., chronic cardiovascular, neurological, neuromuscular, or hepatic disease, hypothyroidism, or clinically significant anaemia) were excluded, as were those with heart failure (serum B-type natriuretic Peptide Test (NT-proBNP) >100 pg/mL), clinically significant echocardiographic abnormality, abnormal markers of liver function (total bilirubin >1.3 mg/dL, direct bilirubin >0.40 mg/dL, aspartate aminotransferase >126 IU/L or alanine transaminase >135 IU/L), elevated haemoglobin A1c (>6.0%), estimated glomerular filtration rate <60 mL/min/1.73 m^2^, resting oxygen saturation of peripheral arterial blood (SpO_2_) <95%, or a history of COVID-related ventilatory support, intensive care, or hospitalisation for >1 week. Participants were invited from the community via word of mouth and through social media advertisements. Data was collected in a single-centre clinical-based setting. Patients gave written informed consent on arrival for the baseline visit. The data collection method for reporting sex data was self-reported by patients.

### Randomisation and masking

Eligible patients were randomised (1:1) using a widely used proprietary interactive response technology (ClinTrak) to receive oral AXA1125 (33.9 g) or matched placebo, which were identical in colour and flavour (orange) with a closely matched aroma and appearance. The clinical research organisation had access to the system which generated the code. The system was password protected, which only authorised researchers had access to. Using the code given from ClinTrak, a prescription, was generated by either MC or BR which was then passed onto the pharmacy. The code corresponded with a non-identifiable box in the pharmacy with either placebo or AXA1125. Both AXA1125 and placebo were concealed using plain white sachets. Patients, study site, pharmacy and study sponsor team members were all blinded to treatment assignment.

### Procedures

**Fig. 1:**
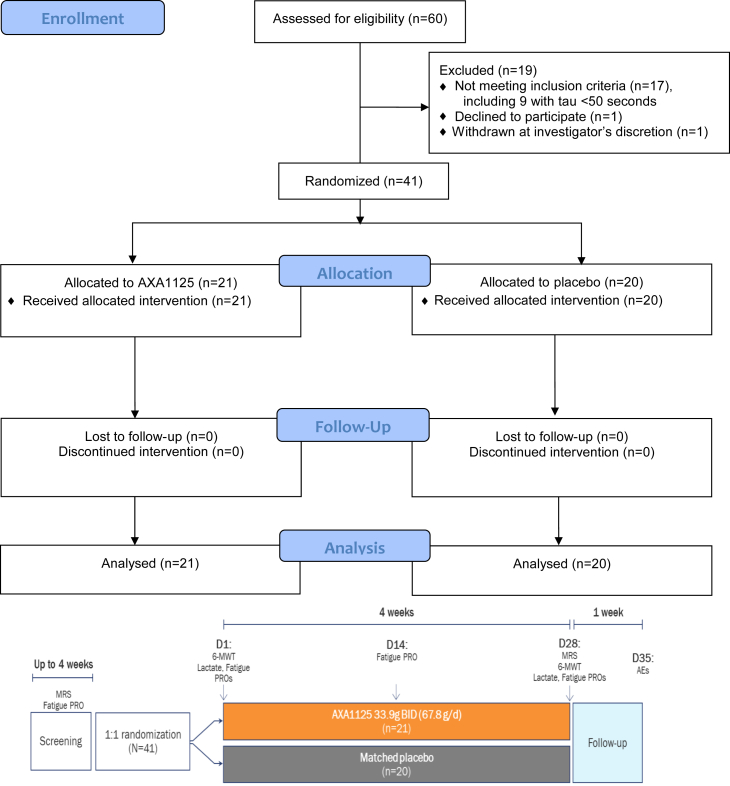
**CONSORT flow diagram and study design**. AE, adverse event; BID, twice daily; CFB, change from baseline; MRS, magnetic resonance spectroscopy; PRO, patient-reported outcome; 6-MWT, 6-min walk test.

Firstly, a screening visit was conducted in a single centre hospital setting which included blood tests, patient-reported fatigue using a validated tool (CFQ-11), echocardiogram and ^31^P-MRS to assess if prospective patients met the inclusion criteria. For those suitable, the study included a 4-week treatment and a 1-week follow-up period, with clinic visits scheduled on Days 1 (baseline) and 28 of treatment, and telephone assessments on Day 14 of treatment and 1 week after treatment completion (Fig. 1). Quantitative evaluations of physical activity (6-min walk test [6MWT]), venous serum lactate levels (immediately before and after the 6MWT, then 5, 10, 20 and 30 min post-6MWT via earlobe puncture) and CFQ-11^11^ were conducted at the Day 1 and Day 28 clinic visits. Pre- and post-exercise τ_PCr_ per ^31^P-MRS were performed on screening visit and 28. An additional assessment of fatigue was conducted during the telephone visit on Day 14. Treatment-emergent adverse events (TEAEs) were solicited using non-leading questions at each study visit through to the final telephone visit (Day 35) and categorised by severity, seriousness, and relationship to the study drug. Adverse events (AE) were categorised using grades 1–5 using Common Terminology Criteria for Adverse Events (CTCAE). Grade 1 was mild, symptomatic or mild symptoms. Grade 2 was moderate, minimal, local non-invasive intervention indicated. Grade 3 was severe or medically significant such as hospitalisation. Grade 4 was life threatening consequences. Grade 5 was death related to AE. Physical examination, vital signs, clinical chemistry, haematology, and urinalysis were conducted at each clinic visit. Study design flow chart can be found in Fig. 1.

Each patient fully enrolled was provided with either AXA1125 or placebo which was reconstituted as a suspension in approximately 180 mL of water and administered twice daily for 4 weeks, with a minimal interval of 4 h between consecutive doses. Patients were required to fast (water was permitted) for ≥10 h prior to study drug administration on clinical visit days. Patients were instructed to maintain their usual dietary and physical activity patterns for the duration of the study.

#### Six-minute walk test

The 6-MWT is a sub-maximal exercise test used to assess aerobic capacity and endurance and is commonly employed as a measure of functional status in patients with chronic respiratory disease.^12^ Patients were asked to walk as far as possible along a 20-m pre-marked corridor for a period of 6 min, and the distance covered in this time was recorded. In accordance with European Respiratory Society/American Thoracic Society guidelines, patients were allowed to take as many standing rests as required but were offered no physical assistance to complete the task.^13^ Results were analysed as both the absolute distance covered as well as the proportion of predicted distance based on correction for age and sex, as detailed below.^14^

▪ Men: (7.57 × height) − (5.02 × age) − (1.76 × weight) − 309
▪ Women: (2.11 × height) − (5.78 × age) − (2.29 × weight) + 667

(Height (cm), age (years), weight (kg))

#### Phosphorus magnetic resonance spectroscopy

^31^P-MRS was used to estimate the skeletal muscle concentration of high-energy phosphate compounds and to characterise the bioenergetic state of skeletal muscle tissue *in vivo*. Dynamic ^31^P-MRS permits assessment of the bioenergetic response of ^31^P metabolites to exercise. Upon exercise cessation, the phosphocreatine recovery rate serves as a marker of oxidative metabolism (in the absence of significant pH changes).

Post-exercise skeletal muscle recovery rate time constant (τ_PCr_) was determined from dynamic ^31^P-MRS assessments in the gastrocnemius medialis muscle of the calf following a single bout of exercise using a resistance band while in the scanner. Patients were positioned supine on the bed of the 3T Prisma MRI system (Siemens Healthineers, Erlangen, Germany) with their calf muscle over a flexible surface coil (11 cm in diameter, Rapid Biomedical, Rimpar, Germany) and an exercise resistance band was stretched around the dominant foot. After anatomical localisation of individual calf muscles, a depth-resolved surface coil spectroscopy (DRESS) localisation slab, was used to acquire signal from the active gastrocnemius and to avoid signal from the less active soleus muscle.^15^ Following ∼20 min of rest, patients were asked to extend their foot at a frequency of one extension every 2 seconds (using a metronome as an auditory pacing cue). Signal acquisition occurred during periods of rest between flexions. Patients were asked to continue this exercise for 3 min unless they were unable to maintain the same intensity at which point the exercise would stop. Recovery data were acquired during a further 8 min of rest, after which the patients were taken out of the MRI scanner. To avoid bias from previous exercise, ^31^P-MRS was never performed following the 6MWT. Previously reported τ_PCr_ in human gastrocnemius muscle are (mean) 31–50 s in healthy volunteers and increased in some disease states.^16^

Acquired spectra were fitted using the Oxford Spectroscopy Analysis (OXSA) fitting toolbox,^17^ assuming Lorentzian lineshapes for all peaks, and fitting PCr and inorganic phosphate (Pi) as singlets, gamma and alpha adenosine triphosphate (ATPs) as doublets and beta ATP as a triplet. The dynamics of PCr recovery were fitted by a monoexponential curve to compute the τ_PCr_.^16^ Intramyocellular pH was then calculated from the chemical shift between PCr and Pi using the Henderson–Hasselbach equation.

#### Chalder Fatigue Questionnaire

The CFQ-11 instrument is a self-administered questionnaire comprising 11 questions covering two dimensions of fatigue severity, mental fatigue (4 questions) and physical fatigue (7 questions).^18^ CFQ-11 has been validated in diverse patient populations, including those with myalgic encephalomyelitis/chronic fatigue syndrome,^19^ which shares similar clinical features – notably post-exertional fatigue ‒ and also a putative pathogenic role for redox imbalance, with fatigue-predominant Long COVID.^20^ CFQ-11 has also been used in studies of patients with Long COVID.^21^, ^22^, ^23^, ^24^ It has two scoring systems, Likert and bimodal. For patient screening, the bimodal scoring system was used: a score of either 0 (symptom absent) or 1 (symptom present) is assigned to each question, resulting in a total fatigue score ranging from 0 to 11. The Likert scoring system, where each question is answered on a 4-point scale ranging from asymptomatic (0) to maximally symptomatic (3), generating a total fatigue score of 0–33, was then used to assess physical, mental, and total (physical + mental) fatigue before and after the intervention. Physical and mental fatigue severity was categorised by Likert and Bimodal scores respectively as ‘normal’ (Likert 0–9, bimodal 0‒3), ‘mild’ (10–15, and 4–7), or moderate to severe (≥16, and ≥8).

### Outcomes

The primary efficacy endpoint was the mean change in τ_PCr_ from baseline to Day 28, chosen to reflect potential changes in mitochondrial oxidative metabolism rather than as a surrogate endpoint for Long COVID disease progression. Secondary efficacy endpoints, chosen to further understand changes in relevant biology, included (i) change from baseline in pre-exercise CFQ-11 fatigue score; (ii) proportion of patients with improvement in pre-exercise fatigue severity per CFQ-11 at Day 28; (iii) change from baseline in distance covered during a 6MWT, expressed as the ratio of observed distance to age- and sex-predicted distance^13^; and (iv) change from baseline in post-6MWT peak serum lactate level.

Post-hoc comparisons were performed as part of the ancillary analysis to compare improvements in symptoms between those that responded (responders) to the treatment and those that did not (non-responders). Correlations were explored between fatigue score, τ_PCr_, 6MWT performance, and peak serum lactate level.

### Sample size estimations

Sample size estimations based on the primary efficacy endpoint (above) indicated that a study population of 32 patients would provide 80% power at a 2-sided, 5% significance level to detect a 10-s absolute difference in ^31^P-MRS biomarker response between the AXA1125 and placebo groups, based on an expected standard deviation (SD) of 10 s from the published literature.^25^ Assuming a 20% drop-out rate, the recruitment target for study enrolment was approximately 40 patients (equal allocation of 20 patients per group).

An expected standard deviation of 10 s was used as previous literature in healthy individuals were reported to have a τ_PCr_ of up to 50 s in the calf muscle, whereas disease states are associated with prolonged τ_PCr_ of 60 s.^26^ Therefore, a minimum difference of 10 s was shown to be sufficient to distinguish metabolic disease states from healthy states.

### Statistical methods

Efficacy analyses were conducted using the intent-to-treat (ITT) population, comprising all randomised patients (cohort A) who received ≥1 dose of the study drug, based on the treatment to which they were randomised, regardless of the treatment received. The data was completed with no missing data points, therefore there was no need for imputation. Safety was analysed in all patients who received ≥1 dose of study drug, based on the treatment received. Study findings were summarised using descriptive statistics, including mean, SD, median, and ranges for continuous variables, and frequency counts and percentages for categorical variables. Intergroup comparisons for categorical endpoints were performed with the chi-square test. Continuous endpoints were analysed using analysis of covariance models with the change or percent change from baseline as the dependent variable and adjusted for baseline value. As this was a phase 2a study, there was no allowance for multiplicity for the secondary outcomes.

### Ancillary analysis

Separate to the main analysis, we undertook a post-hoc analysis in which intra-group comparisons of outcomes between ‘responders’ and ‘non-responders’ were conducted using Wilcoxon's rank sum test, and correlations were assessed using Spearman's rank correlation. Statistical significance was set at *P* < 0.05. All analyses used SAS statistical software (version 9.4, Cary, NC). Responders were defined as patients who showed an improvement in CFQ-11 scores between baseline and Day 28 in their category of physical fatigue severity (‘responders’) and those who did not (‘non-responders’). The selected comparisons included (τ_PCr_ and 6MWT performance) between patients who showed an improvement between baseline and Day 28 in their category of physical fatigue severity (‘responders’) and those who did not (‘non-responders’). Additional post hoc analyses evaluated correlations between fatigue score and, variously, τ_PCr_, 6MWT performance, and peak serum lactate level. Safety and tolerability endpoints included adverse events and serious adverse events, physical examination findings, body weight, and changes in clinical laboratory assessments.

### Independent data monitoring committee

An independent data monitoring committee was not required given the size and phase of the study.

### Interim analysis and protocol amendment

An interim analysis was performed for the planning of future trials. The clinical trial was not adaptive and the interim result did not alter the trial design or affect stopping guidelines. The research team and patients remained blinded throughout the study, with only the sponsor statistician and chief medical officer aware of the results of the interim analysis. There was no adjustment for bias made following interim analysis. The study team were unaware of these results for the duration of the study, and as such this did not influence the conduct of the study.

An amendment was added to the protocol in August 2022 following cessation of recruitment (May 2022). This was to add a secondary cohort (cohort B) which consisted of patients who had fatigue symptoms (based on CFQ-11) but who did not meet the criteria from the primary endpoint (τ_PCr_ >50 s). The decision to continue with this additional cohort was terminated due to a decision to move forward with planning a larger phase 3 clinical trial.

### Role of the funding source

The funder of the study had a role in study design, data analysis, data interpretation, and writing of the report. BR and MJK had access to the full dataset. The final decision to submit for publication was provided by BR.

## Results

### Main results

Patients were recruited from 15th December 2021 until 23rd May 2023. Out of 60 patients screened, 41 patients met the study eligibility criteria and were randomised to treatment with AXA1125 33.9 g twice daily (BID) (n = 21) or placebo BID (n = 20) (Fig. 1). All patients received ≥1 dose of study treatment (ITT population) and completed the study. The study population had a mean age of 43.6 years (range 24–56); was predominantly female (68%) and Caucasian (90%), displayed moderate to severe fatigue (mean CFQ-11 total fatigue score, Likert scale = 27.1) and reduced physical performance (mean ratio of observed to expected walking distance in 6MWT = 0.846, interquartile range = 0.712–0.982). Demographic and baseline clinical characteristics were generally well balanced across the two treatment groups (Table 1).

**Table 1:** Baseline demographic and clinical characteristics of the ITT population.

	AXA1125 33.9 g BID (n = 21)	Placebo BID (n = 20)
Age, years		
Mean (SD)	43.6 (10.12)	43.6 (7.8)
Range	24–56	29–54
Median (IQR)	48.0 (37.0, 52.0)	(36.0, 51.0)
Sex, n (%)		
Male	6 (28.6)	7 (35.0)
Female	15 (71.4)	13 (65.0)
Race, n (%)		
White	19 (90.5)	18 (90.0)
South Asian	2 (9.5)	1 (5.0)
Other	0	1 (5.0)
Body weight, kg		
Mean (SD)	77.2 (13.9)	77.3 (17.1)
Range	62–111	48–101
Median (Q1,Q3)	73.9 (66.2, 88.4)	(61.1, 92.7)
Body mass index, kg/m^2^		
Mean (SD)	26.4 (4.32)	26.4 (4.25)
Range	20.9–34.8	18.7–33.7
Median (Q1,Q3)	25.0 (23.7, 28.1)	26.6 (22.7, 29.7)
Duration of Long COVID, days^a^		
Mean (SD)	488.9 (210.6)	537.2 (173.0)
Range	123–762	99–795
Median (Q1,Q3)	527 (385.0, 675.0)	525.5 (429.5, 694.0)
6-MWT distance, observed:predicted, %		
Mean (SD)	82.4 (19.6)	86.8 (17.3)
Range	48.0–115.5	57.9–118.7
Median (Q1,Q3)	83.4 (66.8, 100.0)	85.4 (73.4, 97.7)
CFQ-11 total fatigue (bimodal) score^b^		
Mean (SD)	10.48 (1.21)	10.50 (0.89)
Range	6–11	8–11
Median (Q1,Q3)	11 (11, 11)	11 (10, 11)
CFQ-11 physical fatigue (bimodal) score^b^		
Mean (SD)	6.71 (0.78)	6.85 (0.49)
Range	4–7	5–7
Median (Q1,Q3)	7 (7, 7)	7 (7, 7)
CFQ-11 mental fatigue (bimodal) score^b^		
Mean (SD)	3.76 (0.54)	3.65 (0.75)
Range	2–4	1–4
Median (Q1,Q3)	4.0 (4.0, 4.0)	4.0 (3.5, 4.0)
CFQ-11 total fatigue (Likert) score^b^		
Mean (SD)	26.24 (3.59)	28.05 (2.96)
Range	18.0, 32.0	22.0, 32.0
Median (Q1,Q3)	26.00 (24.0, 28.0)	(28.0 (25.5, 31.0)
CFQ-11 physical fatigue (Likert) score^b^		
Mean (SD)	17.29 (2.54)	18.95 (1.70)
Range	10.0, 21.0	14.0, 21.0
Median (Q1,Q3)	17.0 (16.0, 19.0)	19.5 (18.0, 21.0)
CFQ-11 mental fatigue (Likert) score^b^		
Mean (SD)	8.95 (1.56)	9.10 (1.97)
Range	6.0, 12.0	5.0, 12.0
Median (Q1,Q3)	8.00 (8.0, 10.0)	8.5 (8.0, 11.0)

BID, twice daily; CFQ-11, Chalder Fatigue Questionnaire, 11-item; SE, standard error.

a Time from initial COVID-19 diagnosis to randomization to study treatment.

b Pre-exercise fatigue.

### Outcomes

Post-exertional τ_PCr_ for the overall study population on Day 1 (baseline) was high (median = 81.6 s) and showed inter-subject variability (interquartile range = 66.2–115.7 s); similarly, at Day 28 overall τ_PCr_ was elevated and highly variable (median = 88.9 s, interquartile range = 57.9–120.1 s). The mean change in post-exertional τ_PCr_ from baseline to Day 28 was not significant between the AXA1125 and placebo treatment groups (least squares [LS] mean difference, 18.2; 95% confidence interval (95% CI) −12.7, 49.1; *P* = 0.24. The distributions of individual changes (from baseline to Day 28) in post-exertional τ_PCr_ in the AXA1125 and placebo treatment groups are shown in supplement Fig. S1.

**Fig. 2:**
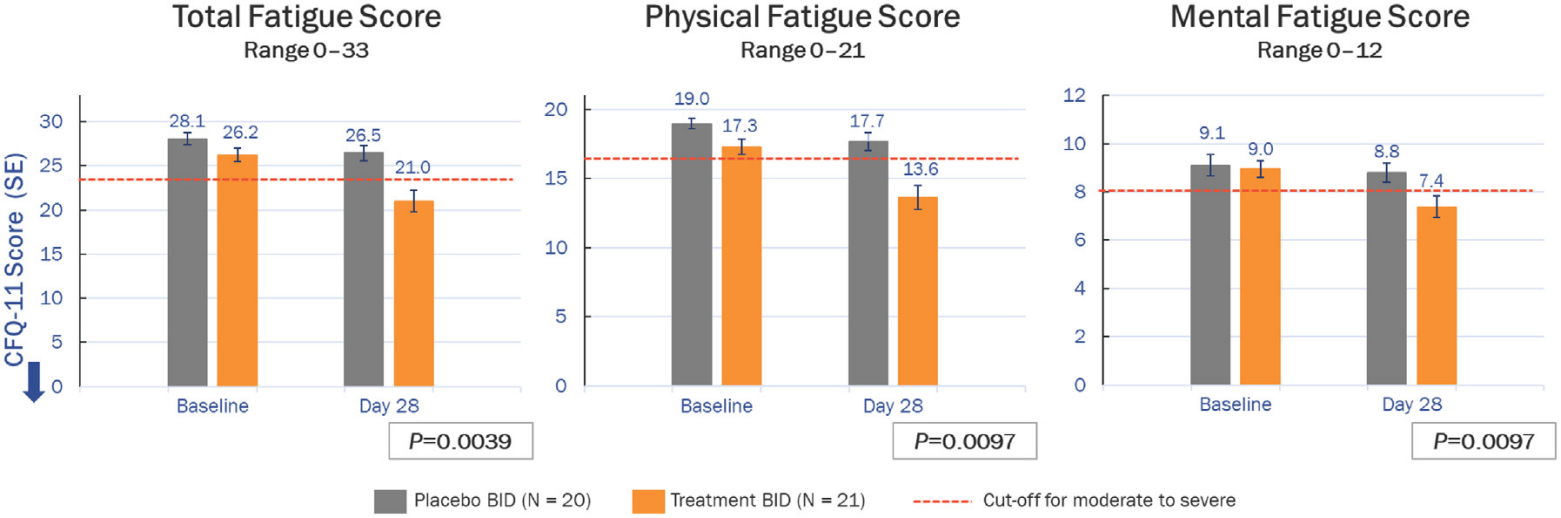
**Mean (±SE) changes from baseline in CFQ-11 total, physical and mental fatigue scores (Likert scale) at Day 28**. The arrow indicates the direction of improvement along the y axis. Moderate-to-severe fatigue is signified by Likert scores of ≥24 (total fatigue), ≥16 (physical fatigue) and ≥8 (mental fatigue). *P* values are from analyses of covariance and represent least squares mean adjusted for differences in baseline. BID, twice daily; CFQ-11, Chalder Fatigue Questionnaire, 11-item; SE, standard error.

**Fig. 3:**
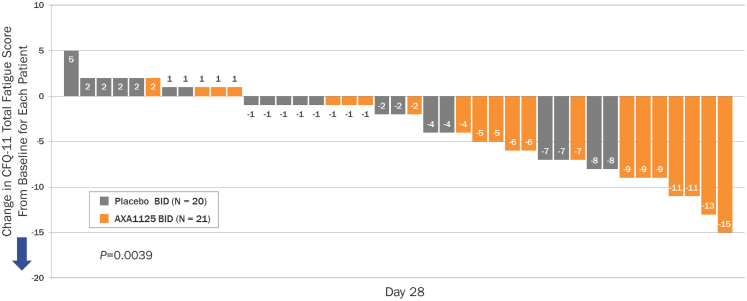
**Waterfall plot showing individual changes in CFQ-11 total fatigue score from baseline to Day 28, per the Likert scale**. The arrow indicates the direction of improvement along the y axis. BID, twice daily; CFQ-11, Chalder Fatigue Questionnaire, 11-item.

**Fig. 4:**
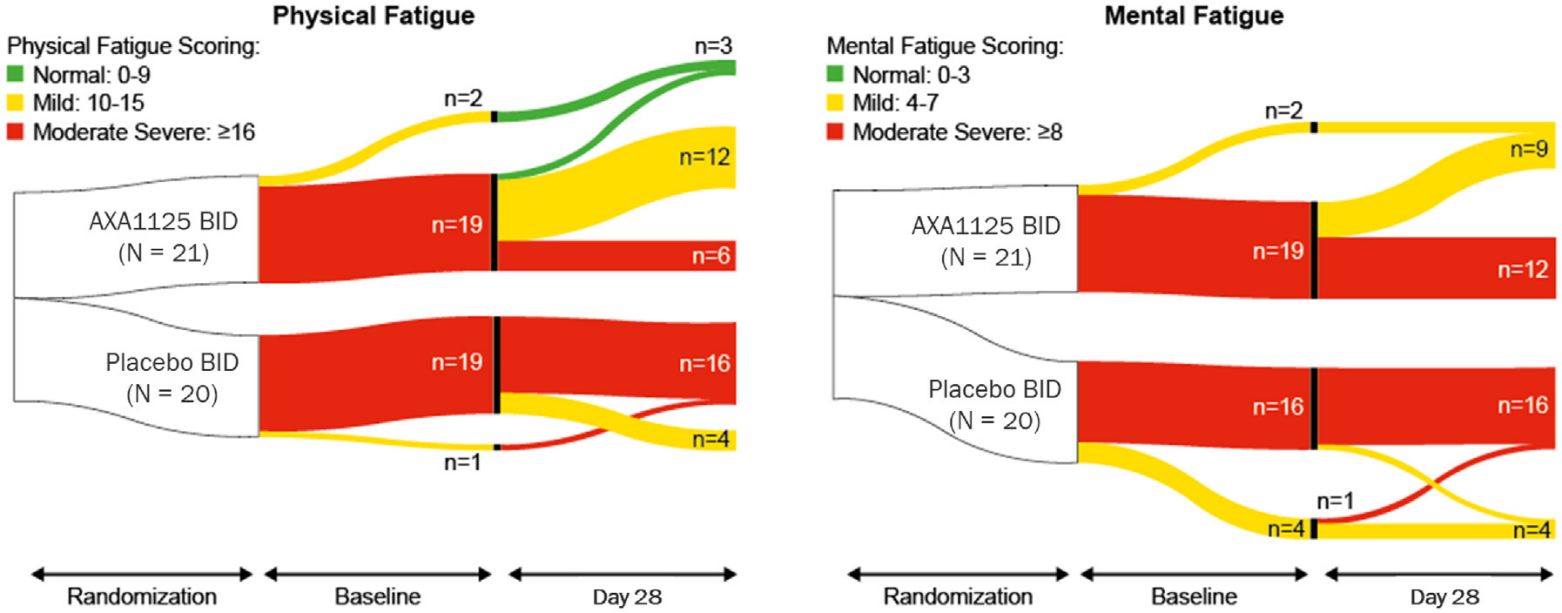
**Sankey plots showing shifts in CFQ-11 physical and mental fatigue severity category from baseline to Day 28**. BID, twice daily; CFQ-11, Chalder Fatigue Questionnaire, 11-item.

A positive correlation was demonstrated between change in pre-exertional physical fatigue score and change in τ_PCr_ in the AXA1125 treatment group (Spearman correlation coefficient, 0.444; *P* = 0.044), but not in the placebo group (Spearman correlation coefficient, 0.386; *P* = 0.0927). Intergroup comparisons indicated significant reductions (from baseline) at Day 28 in pre-exercise CFQ-11 total (least squares [LS] mean difference, −4.30; 95% CI, −7.14, −1.47; *P* = 0.0039), physical (LS mean difference, −2.94; 95% CI –5.12, −0.75; *P* = 0.0097), and mental (LS mean difference, −1.32; 95% CI ‒2.30, −0.34; *P* = 0.0097) fatigue scores with AXA1125 compared to placebo (Fig. 2). A waterfall plot of individual changes in CFQ-11 total fatigue score from baseline to Day 28 indicated marked clustering of responses in the AXA1125 treatment group (Fig. 3). Between baseline and Day 28, pre-exercise physical and mental fatigue improved in 71% (15/21) and 33% (7/21) of AXA1125-treated patients respectively, compared with 20% (4/20) and 5% (1/20) in the placebo arm (Fig. 4). Likewise, AXA1125 produced significant reductions compared to placebo in post-exercise (6MWT) CFQ-11 scores (supplement Table S1), similar in magnitude to those observed in the pre-6MWT fatigue assessment.

AXA1125-treated patients showed a trend towards reduction in post-exertional peak serum lactate level from baseline at Day 28 compared with placebo (LS mean difference, −0.42; 95% CI –0.89, 0.04; *P* = 0.073*)*. The difference in percentage change in post-exertional peak serum lactate was not significant between the two treatment groups (LS mean difference, −24.49; 95% CI –59.20, 10.22; *P* = 0.16*)*. No significant correlation was noted between change in pre-exertional total fatigue score and change in post-exertional peak lactate level in either the AXA1125 group (Spearman correlation coefficient, −0.224; *P* = 0.33) or placebo group (Spearman correlation coefficient, −0.113; *P* = 0.64).

### Ancillary post-hoc analyses

Within the AXA1125 treatment arm, an improvement in phosphocreatine response (i.e, reduction in post exertional τ_PCr_) was observed in patients who experienced an improvement in category of physical fatigue severity (‘responders’) than in those who did not (‘non-responders’), which was significant (*P* = 0.0024; supplement Fig. S1). In contrast, within the placebo arm no significant difference in phosphocreatine response was noted between fatigue ‘responders’ and ‘non-responders’ (*P* = 0.29; Fig. 1c in the supplement). Additionally, within the AXA1125 treatment arm, patients who experienced an improvement in physical fatigue category (‘responders’) showed a significant (*P* = 0.045) increase in walking distance at Day 28 than those who had no improvement in physical fatigue category (‘non-responders’) (supplement Fig. S2). In contrast, within the placebo arm there was no significant difference in the change from baseline in total walked distance (*P* = 0.64) between ‘responders’ and ‘non-responders’ (supplement Fig. S2).

### Efficacy outcomes and HARMS

Results of direct comparisons of efficacy outcomes in the AXA1125 and placebo treatment groups are summarised in Table 2. 11 (52.4%, AXA1125) and 4 (20.0%, placebo) patients reported treatment emergent adverse events (TEAEs) during the study; none were serious, or led to death or treatment discontinuation. In the AXA1125 group, 10 of the 11 TEAEs (91%) were mild in severity and 1 (9%) was severe (syncope, considered treatment-unrelated). In the placebo group, the TEAEs were either mild or moderate in severity (50% each). The most common TEAEs were diarrhea (14.3%, AXA1125 group), abdominal distension (10.0%, placebo group), and nausea (9.5%, AXA1125 group). Among these, only two cases were deemed to be treatment-related. One grade 3 TEAE (syncope associated with the imaging procedure) was reported in the AXA1125 group.

**Table 2:** Summary of efficacy outcome measures at baseline and Day 28 in the AXA1125 and placebo treatment groups.

Parameter	AXA1125 33.9 g BID (n = 21)			Placebo BID (n = 20)		
	Baseline	Day 28	Δ	Baseline	Day 28	Δ
τ_PCr_, sec						
Mean (SD)	96.8 (34.6)	118.0 (68.1)	21.2 (52.2)	87.9 (36.3)	91.9 (43.2)	4.0 (43.0)^†^
Median (Q1,Q3)	87.7 (70.9, 119.0)	111.0 (70.7, 146.2)	4.5 (−12.0, 34.2)	75.2 (63.0, 102.0)	84.8 (55.8, 118.3)	−3.7 (−23.1, 47.7)L
6-MWT distance, m						
Mean (SD)	511.0 (117.1)	536.6 (91.8)	25.57 (54.0)	540.5 (106.5)	565.8 (20.6)	25.3 (12.1)^†^
Median (Q1,Q3)	500 (427.0, 576.0)	500 (480.0, 604.0)	23.0 (0.0, 39.0)	559 (455.0, 651.50)	559.0 (506.0, 636.0)	10 (−3.0, 36.5)
6-MWT distance, observed:predicted, %						
Mean (SD)	82.4 (19.6)	86.7 (17.3)	4.3 (8.5)	86.8 (3.9)	90.8 (14.1)	4.0 (8.6)^†^
Median (Q1,Q3)	(83.4 (66.8, 100.0)	80.0 (76.0, 94.3)	4.3 (0.0, 7.1)	85.5 (73.4, 97.7)	89.0 (82.1, 100.6)	1.7 (−0.5, 5.4)
Peak serum lactate post-6MWT, mmol/L						
Mean (SD)	1.30 (1.1)	1.00 (0.98)	−0.30 (1.0)	1.64 (1.1)	1.65 (1.0)	0.01 (0.6)^†^
Median (Q1,Q3)	1.4 (0.7, 1.6)	0.8 (0.0, 1.5)	−0.1 (−0.90, 0.20)	1.75 (0.75, 2.2)	1.5 (1.00, 2.00)	0.0 (−0.6, 0.45)
CFQ-11 Total fatigue (Likert) score^a^						
Mean (SD)	26.2 (3.59)	21.0 (5.51)	−5.25 (5.49)	28.05 (2.96)	26.45 (3.78)	−2.25 (2.92)∗∗
Median (Q1, Q3)	26.0 (24.0, 28.0)	22 (17.3)	−4.0 (−8.5, −1.0)	28 (25.5, 31.0)	26.5 (24.3)	−1.5 (−4.0, 0)
CFQ-11 Physical fatigue (Likert) score^a^						
Mean (SD)	17.3 (2.54)	13.6 (3.93)	−3.67 (0.75)	19.0 (1.70)	17.7 (2.87)	−1.30 (3.05)∗∗
Median (Q1,Q3)	17.0 (16.0, 19.0)	13.0 (11.0, 16.0)	−3.0 (−6.0, −1.0)	19.5 (18.0, 21.0)	18.0 (16.0, 20.0)	−1.0 (−3.0,-0.50)
CFQ-11 Mental fatigue (Likert) score^a^						
Mean (SD)	8.95 (1.56)	7.38 (2.09)	−1.57 (1.99)	9.10 (1.97)	8.80 (1.80)	−0.30 (1.13)∗∗
Median (Q1,Q3)	8.00 (8.0, 10.0)	8.00 (6.0, 9.0)	−2.0 (−3.0, 0.00)	8.5 (8.0, 11.0)	8.0 (8.0, 10.0)	0.0 (−1.0, 0.5)
CFQ-11 Physical fatigue category, % patients^b^						
Normal	0	3 (14.3)		0	0	
Mild	2 (9.5)	12 (57.1)		1 (5.0)	4 (20.0)	
Moderate-severe	19 (90.5)	6 (28.6)		19 (95.0)	16 (80.0)	
CFQ-11 Mental fatigue category, n (%) patients^c^						
Normal	0	0		0	0	
Mild	2 (9.5)	9 (42.9)		4 (20.0)	4 (20.0)	
Moderate-severe	19 (90.5)	12 (57.1)		16 (80.0)	16 (80.0)	

BID, twice daily; CFQ-11, Chalder Fatigue Questionnaire, 11-item; SE, standard error; 6-MWT, 6-min walk test; τ_PCr_, phosphocreatine recovery rate time constant. ^†^*P* > 0.05, ∗*P* ≤ 0.05, ∗∗*P* ≤ 0.01, ∗∗∗*P* ≤ 0.001.

a Pre-exercise fatigue.

b Physical fatigue categories: normal = Likert score 0–9; mild = Likert score 10–15; moderate-severe = Likert score ≥16.

c Mental fatigue categories: normal = Likert score 0–3; mild = Likert score 4–7; moderate-severe = Likert score ≥8. Comparison of least squares mean change from baseline at Day 28: AXA1125 vs placebo (ANCOVA model).

## Discussion

This study assessed the efficacy, safety, and tolerability of AXA1125 in patients with fatigue-predominant Long COVID. Currently, there is no approved treatment for Long COVID, making this one of the first randomised, double blind, placebo-controlled clinical trials in this disease to demonstrate marked symptomatic improvement. Although a four-week treatment period with AXA1125 was not associated with a statistically significant improvement in post-exercise τ_PCr_, or distance covered during the 6MWT, patients who received AXA1125 did demonstrate a significant reduction in patient-reported fatigue as assessed with a previously established and validated measurement tool.^18^ The absence of a demonstratable treatment effect on τ_PCr_ is likely due to the variability seen in this parameter in this patient population. Nonetheless, the majority of patients had a prolonged τ_PCr_ at baseline, an important finding indicative of significant impairment of mitochondrial metabolism and energetics, highlighting a potential mechanism for ongoing symptoms. The positive trend in τ_PCr_ which accompanied fatigue reduction suggests that treatment response may be due to improvements in metabolism and oxidative stress. These metabolic effects corroborate previous research which found upregulation of fatty acid oxidation in preclinical (hepatocyte)^27^ and clinical (NASH) studies of AXA1125.^10^

Long COVID is still largely defined by patient symptoms rather than by its etiology and the former derives the greatest benefit from AXA1125, a multi-targeted treatment option. The reduction in fatigue obtained with AXA1125, the first to be reported in a placebo-controlled double-blind study, corroborates reports of improvements in patient-assessed Long COVID fatigue in non-randomised studies of amino acid-based nutritional supplements,^28^ and oxaloacetate.^29^ Skeletal muscle mitochondrial function (oxidative capacity) is linked with physical function, physical activity levels and perceived fatigability. Studies employing cardiopulmonary exercise testing have suggested that, by contributing to lower aerobic capacity, impaired mitochondrial function may account for the higher levels of fatigability seen in the elderly.^30^, ^31^, ^32^ The clinical applications of AXA1125 could potentially extend to other post-viral syndromes and chronic fatigue syndrome, which share some biological similarities. ^21^

Treatment with AXA1125 was well tolerated and raised no safety concerns, consistent with short and long-term studies that have established the safety of amino acid administration and the use of AXA1125 in the treatment of non-alcoholic fatty liver disease (NAFLD).^10^ Adverse events reported were mainly gastrointestinal in nature, infrequent, mild in intensity, and classified as AXA1125-unrelated. One severe event (syncope) was deemed unrelated to treatment. No patients discontinued the study due to adverse events. This study was successful using the criteria for a feasibility trial,^33^ which encourages the development of a larger trial, even though the effect sizes may be hard to infer.^34^

A limitation of this study was the variability observed in the primary endpoint; future studies might take this into consideration or employ alternative disease-specific outcome measures to determine treatment efficacy. Another limitation is the absence of other measures of exercise tolerance (e.g. cardiopulmonary exercise testing), omitted given patients’ concerns about a possible relapse of fatigue. Generalisability may also be limited as this was a single centre study. We also did not assess the impact of therapy on Long Covid symptoms other than fatigue. A final limitation would be the small sample size of the study, which is expected for a phase 2 clinical trial of this nature.

In conclusion, there was no significant improvement in τ_PCr_, a measure of skeletal muscle metabolism, which was the primary outcome of this clinical trial. However, administrating AXA1125 twice daily did lead to improvements in both physical and mental fatigue symptoms, which were measured using a validated assessment tool. AXA1125 was well tolerated, with minimal TEAEs reported. A larger randomised placebo-controlled, multicentre efficacy study is currently being planned as part of future work and will include additional assessments of physical function and quality of life in a multicentre setting.

### Registration

This trial is registered at ClinicalTrials.gov, NCT05152849.

### Protocol

The protocol can be accessed alongside this publication.

## Contributors

Conceptualisation: MK, DK, KA, HL, SN, LV, HM, BR

Data curation: LF, MPC, LV, BR

Formal analysis: LF, JP, MK, KA, LV

Funding acquisition: MK, DK, BR

Investigation: LF, MPC, LV, BR

Methodology: MK, DK, KA, HL, SN, LV, HM, BR

Project administration: LF, MPC, LV, BR

Resources: LF, MPC, MK, LV, BR

Software: LV

Supervision: LV, BR

Validation: LV, BR

Visualisation: All authors

Writing original draft: all authors

Writing-review and editing: all authors

The data was presented to the authors formally and available to the authors at request.

KA, MK and BR directly accessed and verified the underlying data reported in the manuscript.

## Data sharing statement

This clinical trial data can be requested by any qualified researchers who engage in independent scientific research and will be provided following review and approval of a research proposal. For more information on the process, or to submit a request contact the following: clinicaltrials@axcellahealth.com.

## Declaration of interests

MK, JP, KA, DK are employees of Axcella Therapeutics and hold stock options in the company. HM has received consultancy fees from Axcella Therapeutics. BR is a consultant and speaker for, and has received research support from, Axcella Therapeutics. LF, MPC, HL and LV have no relevant conflicts of interest to declare. SN has shares in Perspectum Ltd which is the system and methods for gated mapping of T1 values.

## Appendix A Supplementary data

Supplementary material

Protocol

Statistical analysis plan

Caption for supplementary material

## References

[1] World Health Organization (2022). https://covid19.who.int/.

[2] Wulf Hanson S., Abbafati C., Aerts J.G. (2022). A global systematic analysis of the occurrence, severity, and recovery pattern of long COVID in 2020 and 2021. medRxiv.

[3] Healey Q., Sheikh A., Daines L., Vasileiou E. (2022). Symptoms and signs of long COVID: a rapid review and meta-analysis. J Glob Health.

[4] Alfarouk K.O., Alhoufie S.T.S., Hifny A. (2021). Of mitochondrion and COVID-19. J Enzyme Inhib Med Chem.

[5] Srinivasan K., Pandey A.K., Livingston A., Venkatesh S. (2021). Roles of host mitochondria in the development of COVID-19 pathology: could mitochondria be a potential therapeutic target?. Mol Biomed.

[6] Goracy A., Rosik J., Szostak B., Ustianowski L., Ustianowska K., Goracy J. (2022). Human cell organelles in SARS-CoV-2 infection: an up-to-date overview. Viruses.

[7] Sze S., Pan D., Moss A.J. (2022). Overstimulation of the ergoreflex-A possible mechanism to explain symptoms in long COVID. Front Cardiovasc Med.

[8] Vogt H., Ulvestad E., Wyller V.B. (2016). Metabolic features of chronic fatigue syndrome revisited. Proc Natl Acad Sci U S A.

[9] Daou N., Viader A., Cokol M. (2021). A novel, multitargeted endogenous metabolic modulator composition impacts metabolism, inflammation, and fibrosis in nonalcoholic steatohepatitis-relevant primary human cell models. Sci Rep.

[10] Harrison S.A., Baum S.J., Gunn N.T. (2021). Safety, tolerability, and biologic activity of AXA1125 and AXA1957 in subjects with nonalcoholic fatty liver disease. Am J Gastroenterol.

[11] Chilcot J., Norton S., Kelly M.E., Moss-Morris R. (2016). The Chalder Fatigue Questionnaire is a valid and reliable measure of perceived fatigue severity in multiple sclerosis. Mult Scler.

[12] Agarwala P., Salzman S.H. (2020). Six-minute walk test: clinical role, technique, coding, and reimbursement. Chest.

[13] Holland A.E., Spruit M.A., Troosters T. (2014). An official European Respiratory Society/American Thoracic Society technical standard: field walking tests in chronic respiratory disease. Eur Respir J.

[14] Enright P.L., Sherrill D.L. (1998). Reference equations for the six-minute walk in healthy adults. Am J Respir Crit Care Med.

[15] Valkovic L., Chmelik M., Just Kukurova I. (2014). Depth-resolved surface coil MRS (DRESS)-localized dynamic (31) P-MRS of the exercising human gastrocnemius muscle at 7 T. NMR Biomed.

[16] Meyerspeer M., Boesch C., Cameron D. (2020). 31) P magnetic resonance spectroscopy in skeletal muscle: experts' consensus recommendations. NMR Biomed.

[17] Purvis L.A.B., Clarke W.T., Biasiolli L., Valkovic L., Robson M.D., Rodgers C.T. (2017). OXSA: an open-source magnetic resonance spectroscopy analysis toolbox in MATLAB. PLoS One.

[18] Chalder T., Berelowitz G., Pawlikowska T. (1993). Development of a fatigue scale. J Psychosom Res.

[19] Morriss R.K., Wearden A.J., Mullis R. (1998). Exploring the validity of the Chalder Fatigue scale in chronic fatigue syndrome. J Psychosom Res.

[20] Paul B.D., Lemle M.D., Komaroff A.L., Snyder S.H. (2021). Redox imbalance links COVID-19 and myalgic encephalomyelitis/chronic fatigue syndrome. Proc Natl Acad Sci U S A.

[21] Stavem K., Ghanima W., Olsen M.K., Gilboe H.M., Einvik G. (2021). Prevalence and determinants of fatigue after COVID-19 in non-hospitalized subjects: a population-based study. Int J Environ Res Public Health.

[22] Tuzun S., Keles A., Okutan D., Yildiran T., Palamar D. (2021). Assessment of musculoskeletal pain, fatigue and grip strength in hospitalized patients with COVID-19. Eur J Phys Rehabil Med.

[23] Townsend L., Dowds J., O'Brien K. (2021). Persistent poor health after COVID-19 is not associated with respiratory complications or initial disease severity. Ann Am Thorac Soc.

[24] O'Brien K., Townsend L., Dowds J. (2022). 1-year quality of life and health-outcomes in patients hospitalised with COVID-19: a longitudinal cohort study. Respir Res.

[25] Šedivý P., Kipfelsberger M.C., Dezortová M. (2015). Dynamic 31P MR spectroscopy of plantar flexion: influence of ergometer design, magnetic field strength (3 and 7 T), and RF-coil design. Med Phys.

[26] Meyerspeer M., Boesch C., Cameron D. (2021). 31P magnetic resonance spectroscopy in skeletal muscle: experts' consensus recommendations. NMR Biomed.

[27] Russell M., Newgard C., Zhang G., Nitzel A., Hamill M., Azer K. (2022). LIVRQNac increases fatty acid oxidization in a primary human hepatocyte model of non-alcoholic steatohepatitis. J Hepatol.

[28] Rossato M.S., Brilli E., Ferri N., Giordano G., Tarantino G. (2021). Observational study on the benefit of a nutritional supplement, supporting immune function and energy metabolism, on chronic fatigue associated with the SARS-CoV-2 post-infection progress. Clin Nutr ESPEN.

[29] Cash A., Kaufman D.L. (2022). Oxaloacetate treatment for mental and physical fatigue in myalgic encephalomyelitis/chronic fatigue syndrome (ME/CFS) and long-COVID fatigue patients: a non-randomized controlled clinical trial. J Transl Med.

[30] Santanasto A.J., Glynn N.W., Jubrias S.A. (2015). Skeletal muscle mitochondrial function and fatigability in older adults. J Gerontol A Biol Sci Med Sci.

[31] Richardson C.A., Glynn N.W., Ferrucci L.G., Mackey D.C. (2015). Walking energetics, fatigability, and fatigue in older adults: the study of energy and aging pilot. J Gerontol A Biol Sci Med Sci.

[32] Liu F., Wanigatunga A.A., Zampino M. (2021). Association of mitochondrial function, substrate utilization, and anaerobic metabolism with age-related perceived fatigability. J Gerontol A Biol Sci Med Sci.

[33] Lewis M., Bromley K., Sutton C.J., McCray G., Myers H.L., Lancaster G.A. (2021). Determining sample size for progression criteria for pragmatic pilot RCTs: the hypothesis test strikes back!. Pilot Feasibility Stud.

[34] Rothwell J.C., Julious S.A., Cooper C.L. (2022). Adjusting for bias in the mean for primary and secondary outcomes when trials are in sequence. Pharmaceut Stat.

