# Supplementary material for: Efficacy and tolerability of an endogenous metabolic modulator (AXA1125) in fatigue-predominant long COVID: a single-centre, double-blind, randomised controlled phase 2a pilot study
Source: eClinicalMedicine. 2023 Apr 14;59:101946. doi: 10.1016/j.eclinm.2023.101946 (PMC10102537; doi:10.1016/j.eclinm.2023.101946)
Supplement: Supplementary material [file mmc1.docx]

**Supplementary Material**

**Post Hoc Analysis**

**Introduction**

Post hoc responder analyses were used to compare selected outcomes of skeletal muscle recovery rate time constant of phosphocreatine (τ_PCr_) and 6-minute walk test (6MWT) performance between subjects who showed an improvement between baseline and Day 28 in their category of physical fatigue severity (‘responders’) and those who did not (‘non-responders’).

Patients from the AXA1125 treatment arm was grouped into ‘responders’ and ‘non-responders’. The term ‘responder’ is used for a patient who demonstrates an improvement in Chadler fatigue questionnaire (CFQ-11) scores from baseline to day 28, resulting in an improvement in the severity of fatigue.

For example, if a patient before treatment had CFQ-11 Likert score of 17 (which is in the moderate-severe fatigue category) and by the end of end of treatment had a score of 10 (which is in the mild fatigue category), the patient was considered a responder.

**Methods**

Intra-group comparisons of outcomes between ‘responders’ and ‘non-responders’ were conducted using Wilcoxon’s rank sum test, and correlations were assessed using Spearman’s rank correlation. Statistical significance was set at P<.05. All analyses used SAS statistical software (version 9.4, Cary, NC). The selected comparisons included τ_PCr_ and 6MWT performance between responders and non-responders as defined above. Additional post hoc analyses evaluated correlations between fatigue score and, variously, τ_PCr_, 6MWT performance, and peak serum lactate level. Safety and tolerability endpoints included adverse events and serious adverse events, physical examination findings, body weight, and changes in clinical laboratory assessments.

**Results**

An overall summary of post-hoc analyses can be found in Supplement Table 1. Within the AXA1125 treatment arm, a significantly better phosphocreatine response (i.e, reduction in post-exertional τ_PCr_) was observed in patients who experienced an improvement in category of physical fatigue severity (‘responders’) than in those who did not (‘non-responders’) (P=0.0024; Supplement figure 1). In contrast, within the placebo arm no significant difference in phosphocreatine response was noted between fatigue ‘responders’ and ‘non-responders’ (P=0.29; Figure 2 in the supplement). Consistent with these findings, a positive correlation was demonstrated between change in pre-exertional physical fatigue score and change in τ_PCr_ in the AXA1125 treatment group (Spearman correlation coefficient, 0.444; P=0.044) but not in the placebo group (Spearman correlation coefficient, 0.386; P=0.093).

Overall, significant change from baseline in observed 6-MWT distance at Day 28 did not differ between the AXA1125 and placebo groups (Supplement Figure 2). However, within the AXA1125 treatment arm, walking distance at Day 28 significantly improved in responders (P=0.045) but not in non-responders (Supplement Figure 2). In contrast, within the placebo arm, the change from baseline in total walked distance did not differ significantly (P=0.64) between ‘responders’ and ‘non-responders’ (Supplement Figure 2). Consistent with these findings, reduction in pre-exercise physical fatigue score with AXA1125 was associated with improvement in 6MWT observed:predicted performance ratio (Spearman correlation coefficient, –0.661; P=0.0011), whereas no such association was noted in the placebo arm (Spearman correlation coefficient, –0.293; P=0.2107). Responder analysis findings for the separate AXA1225 and placebo treatment arms are presented in Supplement Table 1.

**Discussion**

While no significant difference was observed between the effects of AXA1125 and placebo on post-exercise τPCr, or on distance covered during the 6MWT, patients who demonstrated a reduction in physical fatigue severity with AXA1125 had significantly reduced phosphocreatine recovery rate time constants after MRS exercise and better 6MWT performance than patients with unimproved fatigue. The absence of a demonstrated treatment effect on τPCr is likely due to the unexpected variability seen in this parameter in this patient population.

Supplement Table 1. Summary of fatigue responder analysis findings

|  | AXA1125 33.9 g BID (n = 21) | | |
| --- | --- | --- | --- |
|  | Physical fatigue responders (n=15) | Physical fatigue non-responders (n=6) | *P*-value (Wilcoxon’s test) |
| Mean (SD) change from baseline in τ_PCr_ at Day 28, seconds | 0.42 (33.5)  0.4185 (8.6) | 73 (56.7)  73.2 (23.2) | 0.0024  0.0024 |
| Mean (SD) change from baseline in 6-MWT distance at Day 28, metres | 38.1 (57.7)  38.1333 (14.9012) | -5.8 (38.0)  -5.8333 (15.5) | 0.045  0.045 |
| Mean (SD) change from baseline in post-exertional peak serum lactate level at Day 28, mmol/L | -0.37 (0.99)  -0.3667 (0.2548) | -0.13 (1.04)  -0.1333 (0.42) | 0.73  0.73 |
|  | Placebo BID  (n = 20) | | |
|  | Physical fatigue responders (n=4) | Physical fatigue non-responders (n=16) | *P*-value (Wilcoxon’s test) |
| Mean (SD) change from baseline in τ_PCr_ at Day 28, seconds | -13.6 (13.0)  -13.6 (6.48) | 8.4 (47.0)  8.37 (11.8) | 0.29  0.29 |
| Mean (SD) change from baseline in 6-MWT distance at Day 28, metres | 32 (58.0)  32 (29.0) | 23.6 (54.8)  23.6 (13.7) | 0.64  0.64 |
| Mean (SD) change from baseline in post-exertional peak serum lactate level at Day 28, mmol/L | 0.075 (0.57)  0.075 (0.29) | -0.012 (0.60)  -0.013 (0.15) | 0.57  0.57 |

Abbreviations: BID, twice daily; SE, standard error; 6-MWT, 6-minute walk test; τ_PCr_, phosphocreatine recovery rate time constant.

Supplement Figure 1. Box plots of individual changes from baseline to Day 28 in phosphocreatine recovery time constant in (a) AXA1125 versus placebo-treated patients, (b) AXA1125 responders versus non-responders, and (c) placebo responders versus non-responders.


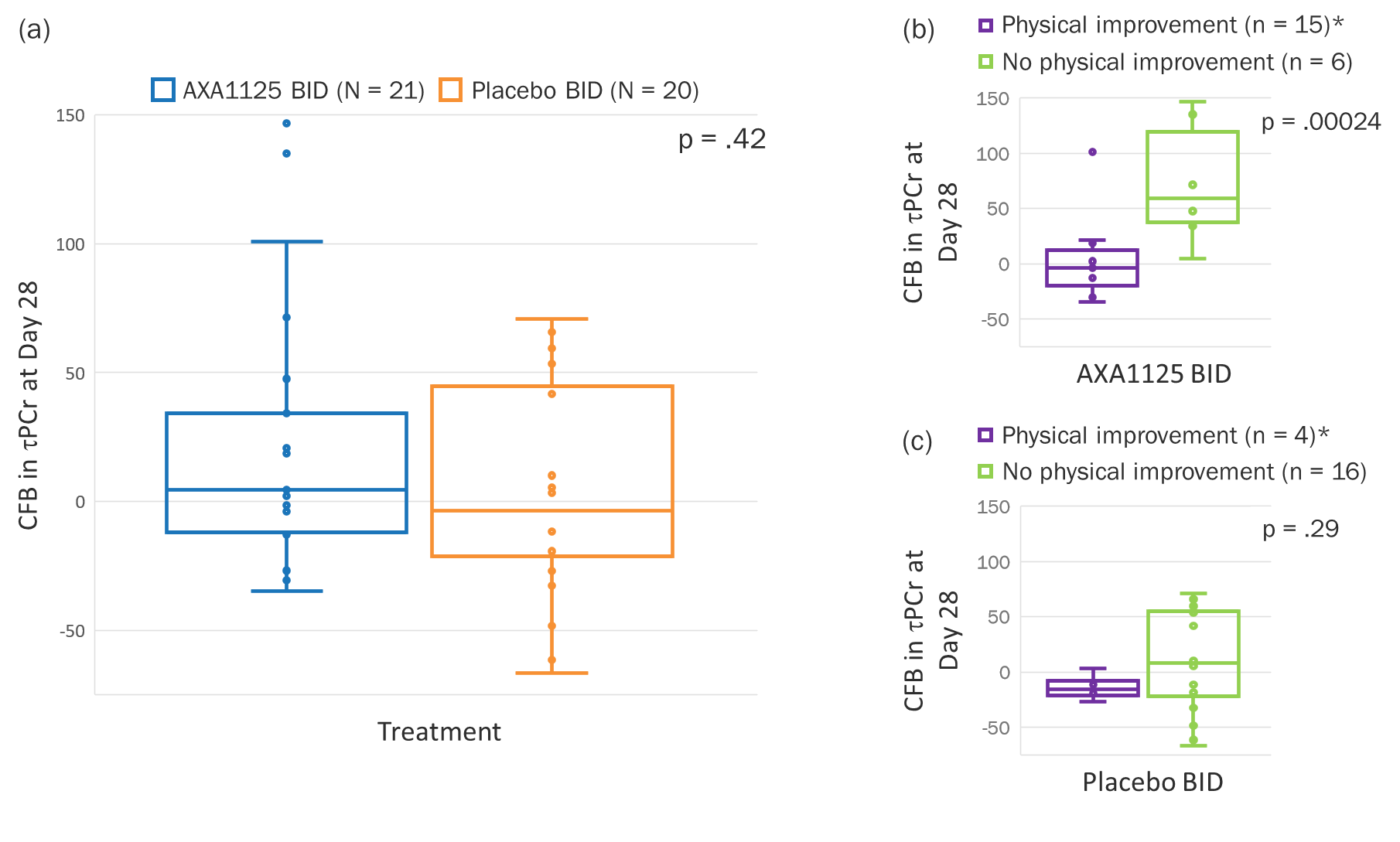


Responders were defined as subjects who experienced an improvement in category of physical fatigue severity (moderate/severe → mild or normal, or mild → normal) between baseline and Day 28. The box extends from the 25th to 75th percentiles, the line represents the median, and the whiskers show minimum and maximum values, excluding outliers. Abbreviations: BID, twice daily; CFB, change from baseline; τPCr, phosphocreatine recovery time constant

Supplement Figure 2. Box plots of individual changes from baseline to Day 28 in total distance covered during 6-minute walk test in (a) AXA1125 versus placebo-treated patients, (b) AXA1125 ‘responders’ versus ‘non-responders’, and (c) placebo ‘responders’ versus ‘non-responders’.


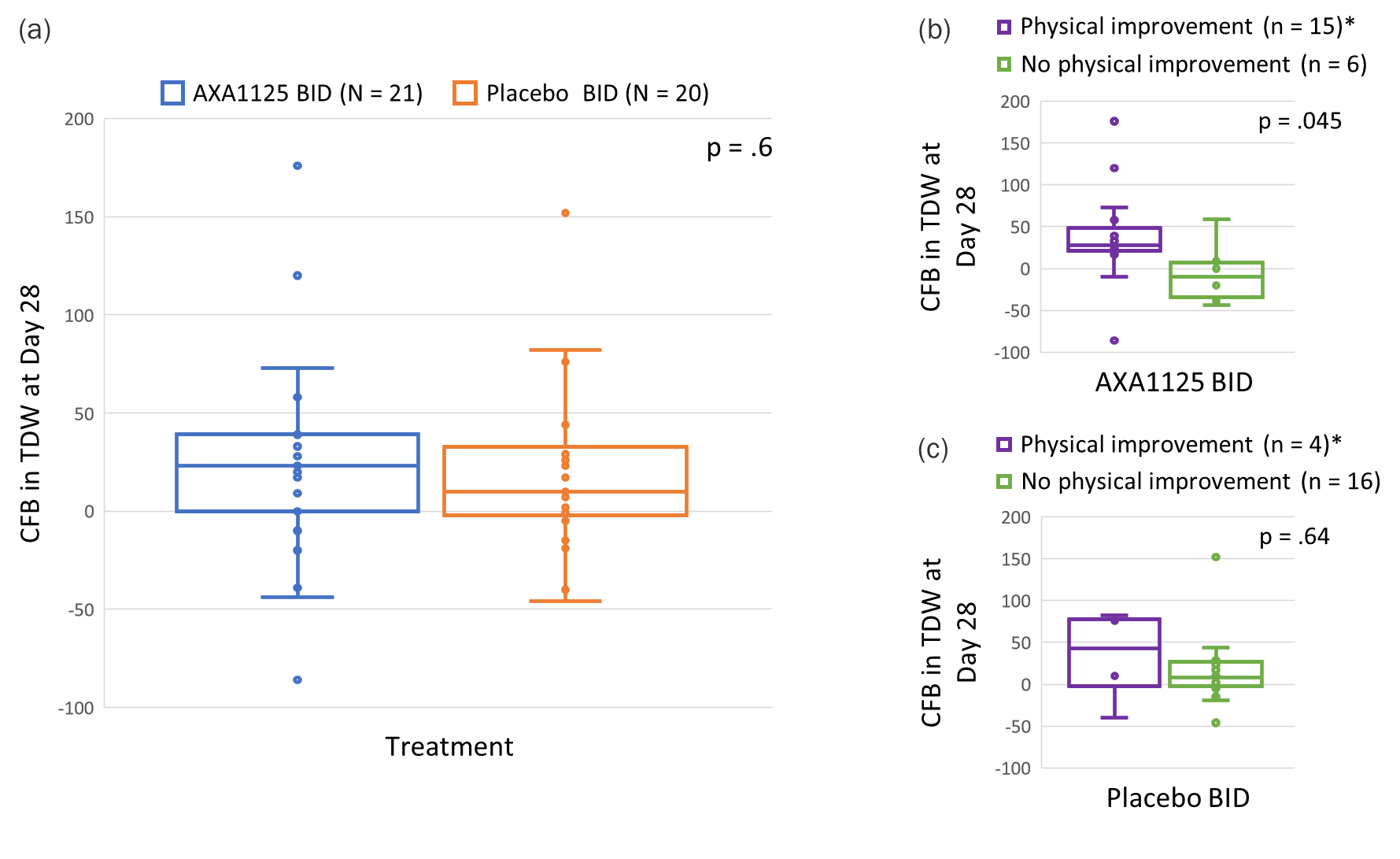


Responders were defined as subjects who experienced an improvement in category of physical fatigue severity (moderate/severe → mild or normal, or mild → normal) between baseline and Day 28. The box extends from the 25th to 75th percentiles, the line represents the median, and the whiskers show minimum and maximum values, excluding outliers. Abbreviations: BID, twice daily; CFB, change from baseline; TDW, total distance walked
