# Supplementary material for: Efficacy and tolerability of an endogenous metabolic modulator (AXA1125) in fatigue-predominant long COVID: a single-centre, double-blind, randomised controlled phase 2a pilot study
Source: eClinicalMedicine. 2023 Apr 14;59:101946. doi: 10.1016/j.eclinm.2023.101946 (PMC10102537; doi:10.1016/j.eclinm.2023.101946)
Supplement: Protocol [file mmc2.docx]

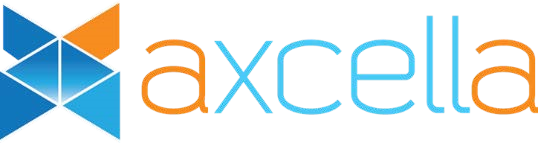


**Protocol Number: AXA1125-201**

**A Randomized, Double-Blind, Placebo-Controlled Pilot Study to Evaluate the Efficacy, Safety, and Tolerability of AXA1125 in Subjects With Fatigue-Predominant Post-Acute Sequelae of SARS-CoV-2 (PASC) Infection**

**Release Date: 03 August 2022** **Version: 4.0**

**Axcella Health, Inc.**

**840 Memorial Drive**

**Cambridge, MA 02139**

**United States**

**Tel: +1-857-320-2200**

This document is a confidential communication of Axcella Health, Inc. The recipient agrees that no information contained herein will be published or disclosed without the prior written approval of Axcella Health, Inc., except that this document may be disclosed to appropriate Institutional Review Board/Independent Ethics Committee or duly authorized representatives of the appropriate regulatory agencies under the condition they are requested to keep it confidential.

# INVESTIGATOR SIGNATURE PAGE

This protocol is sponsored by Axcella Health, Inc. All the data and any other information generated from the execution of this protocol are the sole property of Axcella Health, Inc. and may not be used for any purpose without written permission from Axcella Health, Inc. As the Principal Investigator, I agree to:

- Adhere to the protocol as outlined;
- Conduct this study in accordance with the International Council for Harmonisation (ICH) guidelines for Good Clinical Practices, the current version of the Declaration of Helsinki: Ethical Principles for Medical Research Involving Human Subjects, and applicable local ethical and legal requirements.

Principal Investigator’s Signature:

Principal Investigator’s Name:

Institution Name:

Date (dd MMM yyyy):

# SPONSOR APPROVAL PAGE

| Sponsor’s Name: | Margaret Koziel, MD |
| --- | --- |
| Title: | Chief Medical Officer |
| Address: | Axcella Health, Inc.  840 Memorial Drive  Cambridge, MA 02139 United States  8/5/2022 |
| Date (dd MMM yyyy): |  |

Sponsor’s Signature:


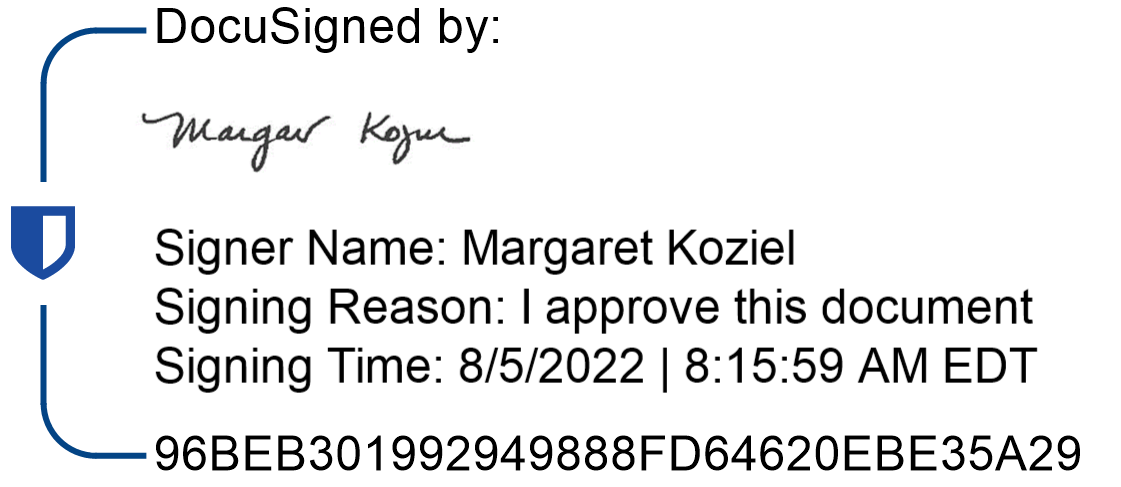


# TABLE OF CONTENTS

[INVESTIGATOR SIGNATURE PAGE _2_](#_Toc168908)

[SPONSOR APPROVAL PAGE _3_](#_Toc168909)

[TABLE OF CONTENTS _4_](#_Toc168910)

[LIST OF TABLES _9_](#_Toc168911)

[LIST OF FIGURES _9_](#_Toc168912)

[LIST OF APPENDICES _9_](#_Toc168913)

[LIST OF ABBREVIATIONS AND DEFINITIONS OF TERMS _10_](#_Toc168914)

[PROTOCOL SYNOPSIS _14_](#_Toc168915)

[1 INTRODUCTION _24_](#_Toc168916)

[1.1 Description of the Disease 24](#_Toc168917)

[1.2 Mode of Action of the Drug Product in PASC 25](#_Toc168918)

[1.3 Published Studies with AXA1125 or Components of AXA1125 25](#_Toc168919)

[1.4 Nonclinical Experience 26](#_Toc168920)

[1.4.1 Pharmacology 26](#_Toc168921)

[1.4.2 Nonclinical safety 27](#_Toc168922)

[1.5 Clinical Experience 27](#_Toc168923)

[1.5.1 Clinical safety data based on published literature 27](#_Toc168924)

[1.5.2 Clinical data for AXA1125 28](#_Toc168925)

[1.5.2.1 Clinical safety data for AXA1125 28](#_Toc168926)

[1.5.2.2 Clinical pharmacokinetic data for AXA1125 29](#_Toc168927)

[1.5.2.3 Clinical efficacy data for AXA1125 30](#_Toc168928)

[1.6 Study Rationale 32](#_Toc168929)

[1.7 Benefit-Risk Assessment 32](#_Toc168930)

[2 OBJECTIVES AND ENDPOINTS _34_](#_Toc168931)

[2.1 Study Objectives – Cohort A 34](#_Toc168932)

[2.1.1 Primary objective 34](#_Toc168933)

[2.1.2 Secondary objective 34](#_Toc168934)

[2.1.3 Exploratory objectives 34](#_Toc168935)

[2.2 Study Objectives – Cohort B 34](#_Toc168936)

[2.3 Study Endpoints 34](#_Toc168937)

[2.3.1 Efficacy endpoints – Cohort A 34](#_Toc168938)

[2.3.1.1 Primary efficacy endpoint 34](#_Toc168939)

[2.3.1.2 Secondary efficacy endpoints 34](#_Toc168940)

[2.3.1.3 Exploratory efficacy endpoints 35](#_Toc168941)

[2.3.2 Efficacy endpoints - Cohort B 35](#_Toc168942)

[2.3.3 Safety and tolerability endpoints - Cohort A and B 35](#_Toc168943)

[3 STUDY DESIGN _36_](#_Toc168944)

[3.1 Overall Study Design 36](#_Toc168945)

[3.2 Scientific Rationale for Study Design 38](#_Toc168946)

[3.3 Justification of Dose and Dosing Regimen 39](#_Toc168947)

[3.4 Study Duration 40](#_Toc168948)

[4 STUDY POPULATION AND SELECTION _41_](#_Toc168949)

[4.1 Inclusion Criteria 41](#_Toc168950)

[4.2 Exclusion Criteria (Cohort A and B) 42](#_Toc168951)

[4.3 Screen Failures and Rescreening (Cohort A and B) 43](#_Toc168952)

[4.4 Subject Enrollment (Cohort A and B) 44](#_Toc168953)

[4.5 Subject Withdrawal (Cohort A and B) 44](#_Toc168954)

[4.5.1 Withdrawal from study participation 44](#_Toc168955)

[4.5.2 Withdrawal from study drug only 45](#_Toc168956)

[4.5.3 Handling of discontinuation of study drug and study withdrawals 45](#_Toc168957)

[4.5.4 Lost to follow-up 46](#_Toc168958)

[5 STUDY INTERVENTIONS (Cohort A and B) _47_](#_Toc168959)

[5.1 Study Drug 47](#_Toc168960)

[5.1.1 AXA1125 47](#_Toc168961)

[5.1.2 Placebo 47](#_Toc168962)

[5.1.3 Study drug storage 48](#_Toc168963)

[5.1.4 Dosage, dose titration, and administration 48](#_Toc168964)

[5.1.5 Missed doses 49](#_Toc168965)

[5.1.6 Blinding 49](#_Toc168966)

[5.1.7 Accountability 49](#_Toc168967)

[5.2 Medications, Supplements, Treatments, and Procedures 49](#_Toc168968)

[5.2.1 Concomitant medications and/or supplements 49](#_Toc168969)

[5.2.2 Allowed medications, treatments, and/or procedures 50](#_Toc168970)

[5.2.3 Prohibited medications, treatments, and/or procedures 50](#_Toc168971)

[5.3 Prevention of Pregnancy 51](#_Toc168972)

[5.4 Dietary Restrictions and Lifestyle Modifications 52](#_Toc168973)

[5.5 Other Restrictions 52](#_Toc168974)

[6 STUDY ASSESSMENTS AND PROCEDURES (Cohort A and B) _53_](#_Toc168975)

[6.1 Study Visits 53](#_Toc168976)

[6.2 Demographics 53](#_Toc168977)

[6.3 Medical, Surgical, and Medication Histories 53](#_Toc168978)

[6.4 Body Measurements 53](#_Toc168979)

[6.5 Laboratory Sample Collections 53](#_Toc168980)

[6.6 Safety and Tolerability Assessments 54](#_Toc168981)

[6.6.1 Physical examination 54](#_Toc168982)

[6.6.2 Vital signs 54](#_Toc168983)

[6.6.3 Echocardiogram 54](#_Toc168984)

[6.6.4 Safety laboratory tests 54](#_Toc168985)

[6.6.4.1 Clinical laboratory assessments 54](#_Toc168986)

[6.6.4.2 Urine screen for drugs of abuse 55](#_Toc168987)

[6.6.4.3 Alcohol breath test 55](#_Toc168988)

[6.6.4.4 Pregnancy tests 55](#_Toc168989)

[6.7 Collection and Reporting of Adverse Events 55](#_Toc168990)

[6.7.1 Adverse event and treatment-emergent adverse event 56](#_Toc168991)

[6.7.2 Serious adverse event 56](#_Toc168992)

[6.7.3 Classification of adverse events 56](#_Toc168993)

[6.7.3.1 Severity 56](#_Toc168994)

[6.7.3.2 Relationship 57](#_Toc168995)

[6.7.3.3 Expectedness 57](#_Toc168996)

[6.7.4 Reporting of adverse event and serious adverse event 58](#_Toc168997)

[6.7.4.1 Adverse event 58](#_Toc168998)

[6.7.4.2 Serious adverse event 58](#_Toc168999)

[6.7.5 Pregnancy reporting 59](#_Toc169000)

[6.7.6 Medical events of interest 59](#_Toc169001)

[6.8 Efficacy Assessments 60](#_Toc169002)

[6.8.1 Phosphorus Magnetic Resonance Spectroscopy and Proton Magnetic Resonance](#_Toc169003)

[Spectroscopy (Cohort A Only) 60](#_Toc169004)

[6.8.2 Chalder Fatigue Questionnaire (CFQ-11 PRO for fatigue) (Cohort A and B) 60](#_Toc169005)

[6.8.3 6-Minute Walk Test (Cohort A and B) 61](#_Toc169006)

[6.8.4 Serum Lactate Test (Cohort A Only) 61](#_Toc169007)

[6.9 Pharmacokinetic Assessments 61](#_Toc169008)

[6.10 Exploratory Assessments (Cohort A Only) 61](#_Toc169009)

[7 STATISTICAL CONSIDERATIONS _62_](#_Toc169010)

[7.1 Sample Size Estimation (Cohort A and B) 62](#_Toc169011)

[7.2 Populations for Analysis (Cohort A and B) 62](#_Toc169012)

[7.2.1 Intent-to-treat analysis set 62](#_Toc169013)

[7.2.2 Safety analysis set 62](#_Toc169014)

[7.2.3 Per-protocol analysis set 62](#_Toc169015)

[7.2.4 Pharmacokinetic analysis set (Cohort A) 62](#_Toc169016)

[7.3 Study Endpoints (Cohort A and B) 62](#_Toc169017)

[7.4 Statistical Methods (Cohort A and B) 63](#_Toc169018)

[7.4.1 General approach 63](#_Toc169019)

[7.4.2 Randomization and blinding 63](#_Toc169020)

[7.4.2.1 Breaking the blind 63](#_Toc169021)

[7.4.3 Handling of dropouts/missing data 63](#_Toc169022)

[7.4.4 Subject disposition 64](#_Toc169023)

[7.4.5 Demographics and baseline characteristics 64](#_Toc169024)

[7.4.6 Safety analyses 64](#_Toc169025)

[7.4.7 Efficacy analyses (Cohort A and B) 64](#_Toc169026)

[7.4.7.1 Primary and key secondary efficacy endpoint analyses 64](#_Toc169027)

[7.4.8 Interim analyses and data monitoring (Cohort A only) 64](#_Toc169028)

[7.4.9 Pharmacokinetic analyses (Cohort A only) 65](#_Toc169029)

[8 SUPPORTING DOCUMENTATION AND OPERATIONAL CONSIDERATIONS (Cohort A and B) _66_](#_Toc169030)

[8.1 Regulatory, Ethical, and Study Oversight Considerations 66](#_Toc169031)

[8.1.1 Subject information and informed consent 66](#_Toc169032)

[8.1.1.1 Written informed consent 66](#_Toc169033)

[8.1.2 Ethical committee and regulatory authority review 66](#_Toc169034)

[8.1.3 Quality assurance and quality control 67](#_Toc169035)

[8.2 Administrative and Legal Obligations 67](#_Toc169036)

[8.2.1 Protocol amendments and study termination 67](#_Toc169037)

[8.2.1.1 Study termination 67](#_Toc169038)

[8.2.2 Study documentation and archive 68](#_Toc169039)

[8.2.2.1 Electronic case report forms 68](#_Toc169040)

[8.2.2.2 Record retention 69](#_Toc169041)

[8.2.3 Study monitoring 69](#_Toc169042)

[8.2.4 Materials control, accountability, and disposition 69](#_Toc169043)

[8.2.5 Confidentiality 69](#_Toc169044)

[8.2.6 Disclosure of data 70](#_Toc169045)

[8.3 Protocol Deviations 70](#_Toc169046)

[8.4 Research Use of Stored Human Samples and Data 71](#_Toc169047)

[8.5 End of Study 71](#_Toc169048)

[9 LIST OF REFERENCES _72_](#_Toc169049)

# LIST OF TABLES

Table 1 Amino Acid Composition Within AXA1125 ......................................................47

Table 2 Excipient Function Within Placebo ....................................................................48

# LIST OF FIGURES

Figure 1 Study Schematic - Cohort A ..............................................................................36

Figure 2 Study Schematic - Cohort B ..............................................................................38

# LIST OF APPENDICES

APPENDIX 1. SCHEDULE OF ASSESSMENTS - COHORT A ...................................................78

APPENDIX 2. SCHEDULE OF ASSESSMENTS - COHORT B ...................................................81

APPENDIX 3. CLINICAL LABORATORY ANALYTES - COHORT A.......................................83

APPENDIX 4. SAFETY CLINICAL LABORATORY ANALYTES – COHORT B .......................85

APPENDIX 5. SUMMARY OF CHANGES ..................................................................................87

# LIST OF ABBREVIATIONS AND DEFINITIONS OF TERMS

**Abbreviation or Term Explanation**

6MWT 6-minute walk test

AA amino acid

ADP adenosine diphosphate

AE adverse event

ALP alkaline phosphatase

ALT alanine aminotransferase

ARDS acute respiratory distress syndrome

AST aspartate aminotransferase

ATP adenosine triphosphate

AUC area under the plasma concentration-time curve

area under the plasma concentration-time curve from time 0 to the last

AUClast

quantifiable concentration

BCAA branched-chain amino acid

BID twice daily

BMI body mass index

BNP B-type natriuretic peptide

CFQ Chalder Fatigue Questionnaire

CKD-EPI Chronic Kidney Disease Epidemiology Collaboration

Cmax maximum plasma concentration

COVID-19 Coronavirus Disease 2019

CRO clinical research organization

CRP C-reactive protein

CSA Clinical Study Agreement

CSR Clinical Study Report

CTCAE Common Terminology Criteria for Adverse Events

DDI drug-drug interaction

DILI drug-induced liver injury DOMS delayed onset muscle soreness eCRF electronic case report form eGFR estimated glomerular filtration rate

| EIU | Exposure In Utero |
| --- | --- |
| EOT | End of Treatment |
| ET | Early Termination |
| ET1 | endothelin 1 |
| FCBP | female(s) of childbearing potential |
| FDA | Food and Drug Administration |
| FGF-21 | fibroblast growth factor-21 |
| FIB-4 | fibrosis-4 index |
| FSH | follicle-stimulating hormone |
| GCP | Good Clinical Practice |
| GGT | gamma-glutamyl transferase |
| GLP | Good Laboratory Practice |
| HbA1c | glycated hemoglobin |
| HBsAg | hepatitis B surface antigen |
| HCG | human chorionic gonadotropin |
| HCV | hepatitis C virus |
| HED | human equivalent dose |
| ^1^H-MRS | proton magnetic resonance spectroscopy |
| HOMA-IR | Homeostatic Model Assessment of Insulin Resistance |
| HIV | human immunodeficiency virus |
| IB | Investigator’s Brochure |
| ICAM-1 | intercellular adhesion molecule-1 |
| ICF | informed consent form |
| ICH | International Council for Harmonisation |
| IEC | Independent Ethics Committee |
| IL-6 | interleukin-6 |
| IRB | Institutional Review Board |
| IRT | Interactive Response Technology |
| ITT | intent-to-treat |
| LIVRQNac | leucine, isoleucine, valine, arginine, glutamine, and N-acetylcysteine |
| MCH | mean corpuscular hemoglobin |
| MCHC | mean corpuscular hemoglobin concentration |
| MCP-1 | monocyte chemoattractant protein-1 |

| MCV | mean corpuscular volume | |
| --- | --- | --- |
| ME/CFS | myalgic encephalomyelitis/chronic fatigue syndrome | |
| MedDRA | Medical Dictionary for Regulatory Activities | |
| mITT | modified intent-to-treat | |
| MPV | mean platelet volume | |
| Mot-C | circulating mitochondrial peptides | |
| MRI-PDFF | magnetic resonance imaging-derived proton density fat fraction | |
| MRS | magnetic resonance spectroscopy | |
| Nac | N-acetyl-L-cysteine | |
| NAFLD | non-alcoholic fatty liver disease | |
| NASH | non-alcoholic steatohepatitis | |
| NOAEL | no observed adverse effect level | |
| PASC | post-acute sequelae of SARS-CoV-2 infection | |
| PCr | phosphocreatine | |
| PD | pharmacodynamic(s) | |
| PEAE | product-emergent adverse event | |
| PI | Principal Investigator | |
| PK | pharmacokinetic(s) | |
| ^31^P-MRS | phosphorus magnetic resonance spectroscopy | |
| PP | per-protocol | |
| Pro-C3 | N-terminal type III collagen pro-peptide | |
| qual | qualitative | |
| QoL | quality of life | |
| RBC | red blood cell | |
| RNA | ribonucleic acid | |
| SAE | serious adverse event | |
| SAP | Statistical Analysis Plan | |
| SARS-CoV-2 | severe acute respiratory syndrome coronavirus 2 | |
| SD | standard deviation | |
| SRM | Study Reference Manual | |
| SOA | Schedule of Assessments | |
| SUSAR | Suspected Unexpected Serious Adverse Reaction | |
| T1/2 | half-life | |
| T2DM | type 2 diabetes mellitus |  |
| TEAE | treatment-emergent adverse event |  |
| TID | 3 times daily |  |
| ULN | upper limit of normal |  |
| USPI | United States Prescribing Information |  |
| VCAM-1 | vascular cell adhesion molecule-1 |  |
| WBC | white blood cell |  |

# PROTOCOL SYNOPSIS

| **Name of Sponsor/Company:** Axcella Health, Inc. |
| --- |
| **Name of Study Drug:** AXA1125 |
| **Title of Study:**  A Randomized, Double-Blind, Placebo-Controlled Pilot Study to Evaluate the Efficacy, Safety, and Tolerability of AXA1125 in Subjects With Fatigue-Predominant Post-Acute Sequelae of  SARS-CoV-2 (PASC) Infection |
| **Brief Title:**  Efficacy, Safety, and Tolerability of AXA1125 in PASC |
| **Study Type and Phase:**  Prospective interventional pilot clinical study |
| **Investigators and Study Sites:**  A single site is expected to enroll approximately 40 subjects (Cohort A) and approximately 10 subjects (Cohort B). |
| **Objectives:**    **Cohort A**  The primary objective is to:   - Assess the impact of AXA1125 on muscle function (metabolism) following exercise The secondary objectives are to: - Assess the relationship between AXA1125 and functional status - Assess the safety and tolerability of AXA1125 The exploratory objectives are to: - Obtain additional insights into the mechanism of action of AXA1125 - Obtain baseline amino acid (AA) profile in subjects with post-acute sequelae of severe acute respiratory syndrome coronavirus 2 infection (PASC)   **Cohort B**   - Assess the relationship between AXA1125 and functional status - Assess the safety and tolerability of AXA1125 |

| **Study Endpoints:**  **Efficacy Endpoints (Cohort A)** The primary efficacy endpoint is:   - The mean change from baseline at Week 4 in the phosphocreatine (PCr) recovery rate following moderate exercise, as assessed by 31P-magnetic resonance spectroscopy (MRS) The secondary efficacy endpoints are: - Absolute and relative change from baseline in PCr recovery rate as assessed by phosphorus magnetic resonance spectroscopy (31P-MRS) at Week 4 - The proportion of subjects with improvement in PCr recovery rate at Week 4 - Absolute and relative change from baseline in serum lactate level after a 6-minute walk test (6MWT) at Week 4 - The proportion of subjects with serum lactate level ≤3 mmol/L after a 6MWT at Week 4 - The proportion of subjects with a decrease in venous serum lactate level from baseline after a 6MWT at Week 4 - Change from baseline in distance traveled during a 6MWT at Week 4 - Change from baseline in subjects’ fatigue score as assessed by Chalder Fatigue   Questionnaire (CFQ)-11 (by Bimodal Scoring) before and after a 6MWT at Week 4and at Day 14 (without 6MWT).   - The proportion of subjects with an improvement in fatigue score as assessed by CFQ-11 before and after a 6MWT at Week 4 The exploratory efficacy endpoints are: - Change from baseline in circulating mitochondrial peptides (eg, Mots-C), metabolomics, proteomics; plasma biomarkers of inflammation, adhesion markers, muscle injury (eg, troponins, creatine kinase, fibroblast growth factor-21) biomarkers, and mitochondrial function/metabolism (~1.5 mL total); nitric oxide biology; immune profiling, and metabolism/phenotypic extracellular acidification rate and oxygen consumption rate at Week 4 - The mean change from baseline in energetically active metabolites measured using proton magnetic resonance spectroscopy (1H-MRS; ie, creatine, intramyocellular lipids, acetyl-carnitine, and carnosine) at Week 4 - Change from baseline in predose plasma concentrations of AAs at Week 4 (see Laboratory Manual for details) - The mean change from baseline in minimal intramuscular pH after exercise, initial PCr recovery rate, adenosine diphosphate concentration at the end of exercise, maximal mitochondrial capacity, and other parameters measured using dynamic 31P-MRS at Week 4 |
| --- |

| **Efficacy Endpoints (Cohort B)**   - Change from baseline in subjects’ fatigue score as assessed by Chalder Fatigue Questionnaire (CFQ)-11 (by Bimodal Scoring) before a 6MWT at Week 4, and at Day 14 (without 6MWT) - The proportion of subjects with an improvement in fatigue score as assessed by CFQ-11 before a 6MWT at Week 4 - Change from baseline in distance traveled during a 6MWT at Week 4 - The proportion of subjects with an improvement in distance traveled as assessed by 6MWT at Week 4   **Safety and Tolerability Endpoints (Cohort A and B)** The safety and tolerability endpoints are:   - Adverse events (AEs) and serious adverse events (SAEs) - Physical examination findings, including vital signs (sitting systolic and diastolic blood pressure, heart rate, respiratory rate, body temperature, resting O_2_ saturation) and body weight - Change in clinical laboratory assessments, including chemistry, hematology, and urinalysis |
| --- |
|  |

| **Study Design: Cohort A**  This is a minimum of one center, randomized, double-blind, placebo-controlled, pilot clinical study. The study will evaluate the efficacy and safety of AXA1125 in subjects with fatigue-predominant PASC (>12 weeks after initial infection).  The total study duration for each subject will be approximately 9 weeks. This study will comprise a Screening Period of up to 4 weeks, a Treatment Period of 4 weeks, and a Follow-up Period of 1 week.  After obtaining informed consent, subjects will be screened, and approximately 40 eligible subjects (approximately 20 subjects per arm) will be randomized in a 1:1 ratio to receive twice daily (BID) oral administration of 33.9 g AXA1125 or a placebo. Doses will be self-administered on Days 1 to 28, inclusive. Subjects will have clinic visits on Day 1 and Day 28, as well as telephone visits on Day 14 and 1 week after completion of study product administration.  The primary efficacy endpoint is the mean change from baseline at Week 4 in the PCr recovery rate following moderate exercise, as assessed by 31P-MRS, which will be evaluated at Screening and End of Treatment or Early Termination. Additional endpoints for assessment of muscle function, safety, tolerability, and exploratory efficacy endpoints will be assessed during the study details of randomization will be provided in the Randomization Plan and Interactive Response Technology (IRT) system. **Cohort B**  An additional cohort (approximately 10 subjects) will be added to the study which will include subjects who were considered screen-failures for Cohort A due to their PCr recovery rate constant (< 50 seconds), provided their CFQ-11 is _≥_8 and they meet all the eligibility criteria  The total study duration for each subject in cohort B will be approximately 6 weeks, which will comprise a Screening Visit (1 week), Baseline Visit, a Treatment Period of 4 weeks, and a Followup Period of 1 week  After obtaining informed consent, subjects will be re-screened and approximately 10 eligible subjects will be randomized in a 1:1 ratio (double-blind, placebo-controlled) to receive either twice daily (BID) oral administration of 33.9 g AXA1125 or a placebo  Doses will be self-administered on Days 1 to 28, inclusive. Subjects will have clinic visits on Days 1, and 28, as well as telephone visits on Day 14, and 1 week after completion of study product administration.  The efficacy endpoints for Cohort B include the change from baseline at Week 4 in subjects’ fatigue score, as assessed by Chalder Fatigue Questionnaire (CFQ)-11 (by Bimodal Scoring) before 6 MWT, which will be evaluated at Screening, Baseline, and End of Treatment or Early Termination. CFQ-11 will also be assessed at Day 14 (without 6MWT). Additional endpoints for assessment of functional status, safety, and tolerability will be collected.  Details of randomization will be provided in the Randomization Plan and Interactive Response Technology (IRT) system. |
| --- |

| **Number of Subjects:**  Approximately 40 subjects will be enrolled in Cohort A and 10 subjects will be enrolled in Cohort B at 1 site in the United Kingdom. |
| --- |
| **Eligibility Criteria:**  Subjects must meet all the inclusion criteria and none of the exclusion criteria to be considered for enrollment into this study. **Inclusion Criteria**  A subject must meet all of the following inclusion criteria to be considered for enrollment into this study:  **Cohort A and B**   1. A subject must be male or female 18 years of age or older, and under 65 years of age. 2. A subject must be able to provide written informed consent and be able to understand and willing to comply with specified requirements as stated in the protocol. 3. A subject must have had clinically suspected COVID-19 and a positive antibody test or a documented SARS-CoV-2 infection (a positive reverse transcription polymerase chain reaction test) at least 12 weeks prior to Screening.   Note: Subjects who had a clinical diagnosis of COVID-19, but could not have a PCr test due to the need to self-isolate are allowed.   1. A subject must have fatigue-predominant PASC defined by:   **Cohort A Only**  • an elevated PCr recovery rate constant following moderate exercise on 31P-MRS (τ_PCr_ ≥50 seconds); and  Note: Documented 31P-MRS within 3 months of Screening and after 3 months of acute Coronavirus Disease 2019 (COVID-19) infection is acceptable. If a historical 31P-MRS has been used to determine eligibility, then 31P-MRS should be repeated within 1 month prior to Day 1 (ie, during the Screening Period) to confirm eligibility and establish baseline.     1. Other than PASC, a subject must be in good health without other significant medical or not well controlled medical or psychiatric conditions. Allowable conditions include mild hypertension, dyslipidemia, pre-diabetes as defined as HbA1c _≤_ 6.0%, controlled by diet and/or asthma (mild and not requiring chronic daily treatment). Subjects who are treated for these conditions must be well controlled on a stable regimen (lifestyle modifications and/or medications), for at least 3 months prior to Screening and anticipate no significant alterations to these regimens for the duration of the study. However, doses of certain medications (eg, statins, antihypertensives) used to treat stable chronic conditions may be modified during the study for safety or tolerability issues, if needed at the discretion of the Principal Investigator (PI).   Note: A history of treated hepatitis C virus (HCV) infection is allowed, provided that a negative hepatitis C viral load has been documented at ≥12 weeks after the cessation of |

| hepatitis C treatment. Historical medical record or testing to ensure a negative viral load may be permitted during the Screening Period. HCV ribonucleic acid will be tested in all subjects, including subjects with a history of treated HCV infection or with positive HCV antibody at Screening, provided that a negative hepatitis C viral load has not been documented at ≥12 weeks after the cessation of hepatitis C treatment.   1. A female subject must meet any one of the following criteria: a. Surgically sterile    1. Postmenopausal with ≥12 months of amenorrhea without an alternate medical cause    2. Follicle-stimulating hormone level consistent with postmenopausal state if amenorrheic for <12 months or if <55 years of age    3. If a subject is of childbearing potential, must have a negative serum pregnancy test during Screening and agree to abstain from sexual activity or agree to use a highly effective form of birth control (failure rate <1% per year) for the duration of the study and for at least 30 days after the last dose of study drug 2. A male subject must meet the following criteria:    1. Male subjects with a partner who is a female of childbearing potential (FCBP), must agree to abstain from sexual activity or agree to use a highly effective form of birth control (failure rate <1% per year) for the duration of the study and for at least 90 days after the last dose of study drug. Note: Female partner use of a highly effective form of birth control (failure rate <1%) is acceptable for male subjects participating in the study.    2. Male subjects capable of fathering a child must agree to refrain from sperm donation for the duration of the study and for at least 90 days after the last dose of study drug. 3. A subject must agree to refrain from physical activity outside of normal activities of daily living before Day 1 and for the duration of the study. 4. A baseline CFQ-11 Total score (by Bimodal Scoring) of _≥8._     **Exclusion Criteria (Cohort A and B)**  A subject will be excluded from the study if **any** of the following criteria are met:   1. Other than PASC, have an explanation for fatigue. This includes but not limited to hypothyroidism; chronic cardiovascular, neurological, endocrinological, or peripheral vascular disease; history of major depression; clinically significant anemia; chronic liver disease; rheumatologic diseases requiring treatment with steroids or immunosuppressants; or other cause of neuromuscular disease such as muscular dystrophy. 2. Other than PASC, a history or presence of an uncontrolled, clinically significant disease including:    - Type 1 or Type 2 diabetes mellitus    - A medical condition that may interfere with absorption of the study drug, including but not limited to resection of any part of the gastrointestinal tract, gastric or intestinal bypass, any bariatric surgery, or inflammatory bowel disease |
| --- |

| 1. Meet any of the following during physical exam assessment:    - Resting O_2_ saturation <95% on room air    - Body mass index of <18.5 or >35    - Clinically significant abnormality on echocardiogram (to rule out abnormal cardiac function or elevated pulmonary artery pressure)    - Is pregnant (if female) or lactating 2. Meet any of the following laboratory parameters:    - Serum B-type natriuretic peptide (NT-pro BNP >400 pg/mL)    - Total bilirubin >1.3 mg/dL (>22.23 umol/L) or direct bilirubin >0.40 mg/dL (>6.84 umol/L) unless with a history of Gilbert’s syndrome    - Aspartate aminotransferase (AST) or alanine aminotransferase (ALT) >3 × upper limit of normal (ULN). AST >126 IU/L ALT >135 IU/L    - Glycated hemoglobin (HbA1c) >6.0%    - Human immunodeficiency virus (HIV)-1 or -2 positive    - Estimated glomerular filtration rate <60 mL/min/1.73 m2 calculated using the Chronic Kidney Disease Epidemiology Collaboration equation    - Positive for hepatitis B surface antigen (HBsAg), HCV antibody, or HIV antibody 3. Have a medical history that includes any of the following:    - Non-invasive or invasive ventilatory support for COVID-19    - Intensive care unit or other high dependency unit admission for COVID-19    - Hospitalization for >1 week for COVID-19 without intubation    - Planned or prior organ transplant 4. Treatment with drugs known to cause myopathy in the last 3 months. This includes glucocorticoids, antimalarials, colchicine, antiretroviral drugs, interferon alpha, penicillamine, immune checkpoint inhibitors, tumor necrosis factor inhibitors and statins, if there is a history of statin-induced muscle pain. Note: The Medical Monitor should be consulted if there are questions regarding concomitant medications. 5. Unwilling or unable to stop the use of AA or protein supplements, carnitine, creatine, or N-acetyl-L-cysteine (Nac) at the time of Screening until the end of study 6. A history of inborn errors of metabolism that may impact AA metabolism, including but not limited to urea cycle disorders; 7. A contraindication for a safe magnetic resonance imaging scan, including implanted magnetic metal 8. Any history of illicit drug or alcohol abuse within 6 months prior to Screening. 9. Used an investigational drug, product, or device within 30 days or 5 half-lives (whichever is longer) before Screening, or is enrolled in another investigational drug, product, or device study within 30 days before Screening; Note: Enrollment in registration or observational studies is permitted. |
| --- |

| 1. A contraindication, sensitivity, or known allergy to any ingredient of AXA1125; or 2. Considered, in the opinion of the PI, to be a poor attendee, or for any reason is not able to comply with the study procedures due to reasons such as planned procedures, travel, etc., that would occur during the course of the study     **Retesting (Cohort A and B)**  A single repeat (retest) per analyte or assessment is allowed during the Screening Period to determine eligibility based on the exclusion criteria and per the PI’s discretion (eg, to recheck laboratory values considered to be out of the typical range for an individual).  **Screen Failures and Rescreening (Cohort A and B)**  Screen failures for reasons other than use of prohibited medications(s) may be discussed with the Medical Monitor to determine if rescreening is appropriate. Subjects may be rescreened once with approval from the Medical Monitor; rescreening will require a new subject number. |
| --- |
| **AXA1125, Dose, and Mode of Administration (Cohort A and B):**  AXA1125 is an orally active mixture of 5 specific AAs (leucine, isoleucine, valine, arginine, and glutamine) and Nac (an AA derivative). The active pharmaceutical ingredients and excipients are compounded to produce a uniform dry powder blend for suspension and is provided as a unit dose sachet containing 11.3 g AAs.  Subjects randomly assigned to the active arm will receive 3 sachets of AXA1125 per each dose (33.9 g) BID.  The AXA1125 sachets will be constituted in ~6 fluid oz (~180 mL) of potable water to form a uniformly dispersed suspension and self-administered orally as an orange-colored, orange-flavored drink, taken BID with or without food. Administration around mealtimes is recommended to promote compliance with the BID regimen and should occur at least 4 hours apart. The full dose needs to be taken within 30 minutes from constitution of the study drug. |
| **Placebo, Dose, and Mode of Administration (Cohort A and B):**  The placebo is formulated as a dry powder that has excipient and calorie content similar to  AXA1125. The placebo closely matches the aroma, appearance, color, and taste profile of  AXA1125. The total number of sachets, packaging, constitution instructions, and administration of placebo mirrors AXA1125 to maintain double blinding during the study.  Subjects randomly assigned to the placebo arm will receive 3 sachets of placebo per dose, BID. The placebo sachets will be constituted in ~6 fluid oz (~180 mL) of potable water to form a uniformly dispersed suspension and self-administered orally as an orange-colored, orange-flavored drink, taken BID with or without food. Administration around mealtimes is recommended to promote compliance with the BID regimen and should occur at least 4 hours apart. The full dose needs to be taken within 30 minutes from constitution of the study drug. |
| **Additional Study-Related Materials:** Not applicable. |
| **Duration of Study Drug Exposure:** |

| **Cohort A:** Each subject will receive the study drug (AXA1125 or placebo) BID for up to 4 weeks. **Cohort B:** Each subject will receive the study drug (AXA1125 or placebo) BID for up to 4 weeks. |
| --- |
| **Statistical Methods:**    **General Approach (Cohort A and B)**  Descriptive statistics for continuous variables will include the number of subjects with data to be summarized, mean, standard deviation (SD), median, and range (minimum, maximum). All categorical/qualitative data will be presented using frequency counts and percentages.  The statistical methods for comparison of each endpoint and complete missing imputation methods will be detailed in the Statistical Analysis Plan, which will be developed and completed prior to database lock.  There will be no multiplicity adjustment and all analyses will be performed at the 2-sided 0.05 level of significance.  **Sample Size Estimation Cohort A**  The sample size calculation is based on the primary efficacy endpoint, the mean change from baseline in the PCr recovery rate. Based on literature review, the SD of both the AXA1125 group and the placebo group is assumed to be 10 seconds. To detect a clinically meaningful difference of 10-second improvement, approximately 32 subjects will provide 80% power at a 2-sided, 5% significance level. Assuming 20% dropout rate, approximately 40 subjects (20 subjects per arm) will be enrolled in this study.  This sample size will also allow for a general assessment of safety and tolerability of AXA1125 in this population. **Cohort B** Approximately 10 subjects (5 subjects per arm), who were considered screen-failures for Cohort A due to their PCr recovery rate constant (< 50seconds) provided their CFQ-11 is ≥ 8 and they meet all the eligibility criteria will be enrolled in this Cohort.  **Randomization (Cohort A and B)**  At Visit 2, using an IRT system, eligible subjects will be randomized to receive 33.9 g AXA1125 BID or placebo BID at a treatment allocation ratio of 1:1.  **Safety Analyses (Cohort A and B)**  The safety data will be presented as individual listings and summary tables, including frequency tables for AEs and frequency and shift tables for laboratory evaluations, vital signs, and physical examinations. Shift tables will be generated for key safety parameters, and graphical presentations will be utilized as needed.  **Efficacy Analyses (Cohort A and B)**  The categorical endpoints will be analyzed using the Chi-square test. Subgroup analyses, such as by gender, may be performed. |

| Continuous endpoints will be analyzed using the analysis of covariance models adjusted for the baseline value. Mixed model repeated measure models will be applied as sensitivity analyses.  **Interim Analyses and Data Monitoring (Cohort A only)**  An interim analysis with potential sample size re-estimation will be performed when at least 10 subjects complete their Week 4 visit. Periodical administrative analyses may be performed to closely monitor the study. |
| --- |
| **Date:** 03 August 2022 |

# 1 INTRODUCTION

## 1.1 Description of the Disease

An estimated 10% of Coronavirus Disease 2019 (COVID-19) survivors continue to experience symptoms several weeks to months after the appearance of initial symptoms, a condition termed post-acute sequelae of severe acute respiratory syndrome coronavirus 2 (SARS-CoV-2) infection (PASC; also known as “long COVID”).

PASC is a chronic, multi-organ disease predominantly characterized by fatigue and muscle weakness (Lopez-Leon 2021). Although many patients recover from COVID-19 within several weeks, a substantial proportion of patients exhibit persistent or new symptoms more than 4 weeks after being diagnosed (NICE Guideline 2021). These patients with persistent post-acute COVID-19 symptoms are often referred to as suffering from PASC. It is estimated that PASC affects 20% to 70% of the survivors of acute infection. Many cross-sectional and cohort studies report that chronic fatigue is the most frequently reported symptom following recovery from acute COVID-19 (Crook 2021). A recent large database of nearly 2 million individuals diagnosed with COVID-19 estimated that 23.2% of patients report at least 1 post-COVID-19 condition, and fatigue is among the 3 most common complaints (FAIR Health 2021), typically reported in more than half of subjects with persistent symptoms. Reports of fatigue are independent of the severity of initial illness (Townsend 2021). There is evidence of substantial negative impact on quality of life (QoL) (Halpin 2020), and given the large number of survivors with PASC, it is reasonable to assume that there will be substantial long-term effects not only on individuals but also on the health care system. In a study that examined 1-year outcomes in hospital survivors with COVID-19, only 76% had returned to a pre-COVID-19 level of employment, with 32% of individuals attributing this to decreased physical function (Huang 2021).

Knowledge of pathogenic mechanisms underlying PASC is still evolving. However, parallels of PASC-fatigue with post-exertional malaise observed in myalgic encephalomyelitis/chronic fatigue syndrome (ME/CFS) may be drawn to indicate mechanistic similarities to redox imbalance (increased nitrosative and oxidative stress), chronic inflammation, reduced adenosine triphosphate (ATP)/energy production due to ineffective utilization of carbon sources, elevated lactate and lactate dehydrogenase, and reduced oxidative phosphorylation and mitochondrial dysfunction, collectively leading to a lowered bioenergetic state termed hypometabolism (Paul 2021, Wong and Weitzer 2021,

Komaroff and Lipkin 2021). Other studies (Fluge 2021) have reported metabolic changes in ME/CFS patients, including altered utilization of substrates for energy metabolism, such as increased use of amino acids (AAs) and fatty acids for tricarboxylic acid fueling, with reduced glucose and pyruvate oxidation. Impaired pyruvate dehydrogenase function due to increased expression of pyruvate dehydrogenase kinases may indicate chronic activation of physiological metabolic programs that normally protect cellular energy (ATP) supply under demanding conditions, such as endurance exercise, hypoxia, or starvation. Metabolic adaptations aiming to maintain and restore energy supply may be caused by an underlying tissue hypoxia on exertion in ME/CFS.

Similar to PASC, ME/CFS is often identified with a known infection or an infectious prodrome and includes common symptoms such as fatigue, neurological/pain, neurocognitive/psychiatric, neuroendocrine, autonomic, and immune symptoms, with both ME/CFS and PASC patients having long symptom durations, reduced daily activity, and profound post-exertional malaise.

Currently, there are no approved treatments for PASC.

## 1.2 Mode of Action of the Drug Product in PASC

Mitochondria play a key role in cellular energetics toward aerobic ATP production through oxidative phosphorylation. Disruption of mitochondrial function has been observed in acute SARS-CoV-2 infection of cells as a consequence of both viral hijacking of cellular machinery as well as the innate antiviral immune response (Prasun 2021, Ramakrishnan 2021). Analysis of plasma from patients with acute COVID-19 shows increased oxidative stress and inflammation (Shi 2021). The mechanistic understanding of post-exertional fatigue in PASC is still evolving; however, the combined learnings from acute COVID-19 and ME/CFS suggest the skeletal muscle weakness underpinning fatigue in PASC could be due to defective cellular bioenergetics with compromised mitochondrial metabolism, redox imbalance, vascular insufficiency, and inflammation.

Literature and data supporting this hypothesis are summarized and discussed below.

## 1.3 Published Studies with AXA1125 or Components of AXA1125

Leucine, Isoleucine, and Valine

Branched-chain amino acids (BCAAs; leucine, isoleucine, and valine) are well known in regulating protein synthesis, cellular metabolism, and signaling (_Holeček_ 2018).

These essential AAs are extensively studied for muscle mass and strength in the context of aging, sarcopenia, musculoskeletal disorders, and exercise. While specific literature demonstrating the role of BCAAs in fatigue-predominant PASC are not readily available, literature focused on BCAAs in context of central and peripheral fatigue from non-COVID-19 indications is abundant and indicative of improvements in post-exertional fatigue (Newsholme and Blomstrand 2006, VanDusseldorp 2018). Individuals who supplemented with BCAAs before a squat exercise experienced reduced delayed onset muscle soreness (DOMS) and muscle fatigue compared to the placebo group (Mittleman 1998). BCAAs have been shown to decrease muscle damage, which may help reduce the length and severity of DOMS. Several studies show that BCAAs decrease protein breakdown during exercise and decrease levels of creatine kinase, which is an indicator of muscle damage (Blomstrand 1997, Gualano 2011, Portier 2008).

The BCAA leucine activates the mammalian target of rapamycin pathway in the body that stimulates muscle protein synthesis, which is the process of making muscle (Riazi 2003, Shimomura 2006). In

1 study, people who consumed a drink with 5.6 g of BCAAs after their resistance workout had a

22% greater increase in muscle protein synthesis compared to those who consumed a placebo drink (Kimball and Jefferson 2006). In 2 studies, participants who supplemented with BCAAs improved their mental focus during exercise, which is thought to result from the fatigue-reducing effect of BCAAs (Gee and Deniel 2016, Howatson 2012). Therefore, supplementing with BCAAs, especially before exercise, may speed up recovery time (Kephart 2016, van Hall 1995). Plasma profiling studies in patients with ME/CFS indicate reduced levels of isoleucine, leucine, aromatics, and other metabolites relevant for tricarboxylic acid cycle (Fluge 2016, Germain 2017). In essence, the studies suggest metabolic dysregulation is due to insufficient ATP production from oxidative phosphorylation with concomitant lactic acid accumulation upon exertion and substantially decreased exercise tolerance, postulated to be originating in the muscle. BCAAs can thus modulate metabolism, reduce lactate, and provide protein synthesis support to aid in post-exercise fatigue recovery in PASC.

Glutamine, N-acetyl cysteine

Glutamine is a conditionally essential AA known to regulate cell growth, bioenergetics, and redox

balance that plays a critical role in maintaining the health of gut mucosal barrier to aid in reducing systemic inflammation. Analysis of plasma from patients with COVID-19 (acute or chronic) shows increased oxidative stress and inflammation (Shi 2021, Xiao 2021). Reduced levels of plasma glutamine were observed in several COVID-19 patients. Additionally, the role of glutamine in reducing post-exertional fatigue is demonstrated through its glycogenic effect and glutathione synthesis support (Coqueiro 2019, Medved 2004). Reduction in glutathione levels exacerbates reactive oxygen species, which impacts immune cell function and contributes to sequelae of COVID-19. Nac, a derivative of cysteine, is shown to improve redox imbalance and counter inflammatory processes resulting from dysregulated immune system observed in COVID-19 (Shi and Puyo 2020). Nac is shown to increase muscle strength or resistance to fatigue induced upon exertion by improving reductive balance through increased bioavailability of cysteine and glutathione. Improvements in oxidative damage, antioxidant enzymes, grip strength, and body mass were noted in 6-month-old, dysferlin-deficient Bla/J mice and wild-type C57BL/6 mice when treated with 1% Nac for 10 weeks (García-Campos 2020). Arginine

Arginine is a metabolic regulator that directly modulates vascular dilation through nitric oxide signaling and indirectly regulates inflammation. Plasma arginine levels are deficient in COVID-19 patients, potentially due to elevated arginase activity (Reizine 2021, Tsuda 2019). The combination of arginine and other essential AAs has been shown to improve mitochondrial energetics in context of liver and other diseases, including COVID-19 (Altay 2021). Additionally, in a crossover study of arginine (3.6 g/day), valine (2.2 g/day), and serine (0.2 g/day) in 39 subjects, improvements in fatigue parameters, visual analog scale, and a rating of perceived exertion, along with reduction in serum ketone bodies relative to placebo, support the role of these AAs in improving post-exertional fatigue (Tsuda 2019). In COVID-19-associated acute respiratory distress syndrome (ARDS), distinct immunosuppression is also reported in severe patients due to lymphopenia, along with T cell suppression mediated by myeloid-derived suppressor cells that correlated with a worse intensive care unit outcome. Prolonged reduction in plasma arginine levels due to enhanced arginase activity were also noted in these patients. Although mechanistically unclear, in vitro treatment of peripheral blood mononuclear cells derived from the COVID-19 ARDS patients with L-Arginine significantly improved T cell proliferation, suggesting immunomodulatory potential of arginine in COVID-19 (Reizine 2021). Emerging understanding of chronic COVID-19 complications suggests treatments directed at addressing multiple complications, such as inflammation, endothelial dysfunction, coagulopathies, and related, are likely to benefit over single-mechanism therapies (Gu 2021). Overall, arginine has the potential to address vascular insufficiency and inflammation, restore redox balance through lowering of nitrosative stress with further support to nitric oxide signaling, relieve immune suppression, and additionally boost mitochondrial energetics.

## 1.4 Nonclinical Experience

### 1.4.1 Pharmacology

The pharmacology of AXA1125, using a related AA composition with the same AA constituents as AXA1125 (leucine, isoleucine, valine, arginine, glutamine, and N acetylcysteine [LIVRQNac]), has been explored nonclinically in both cell and rodent models in non-Good Laboratory Practice (GLP) settings. In the in vitro studies, the constituents of LIVRQNac were added at specified fold concentrations above normal plasma levels, 10× to 30× for LIVRQNac is not endogenous in plasma and was added at 2.5 to 7.5 mM). These concentrations are predicted to reflect AA concentrations in the hepatic inlet after administration of AXA1125.

LIVRQNac treatment positively impacted the representative metabolic, inflammatory, and fibrotic phenotypes in non-alcoholic steatohepatitis (NASH)-relevant primary human cell systems, such as hepatocyte model of lipotoxicity, lipopolysaccharide-stimulated macrophage model of inflammation, and transforming growth factor beta-1-stimulated stellate cell model of fibrosis. Primary human cell in vitro systems demonstrated that while individual AA constituents of AXA1125 can impact specific NASH-relevant phenotypes, the full composition is necessary to broadly impact the full range of metabolic, inflammatory, and fibrotic phenotypes. Similarly, administration of LIVRQNac in a murine model of NASH (STAM™) improved non-alcoholic fatty liver disease (NAFLD) Activity Score and liver fibrosis while also reducing liver cytokine/chemokine levels. The in vitro data are consistent with Axcella’s understanding of the mechanism of action of the constituent AAs in AXA1125 based on data from the literature and, therefore, their contribution in a multitargeted approach to treat the main drivers of NASH pathogenesis (figure for mechanism of action to be added, if needed).

Please refer to the current version of the Investigator’s Brochure (IB) for the available information concerning the nonclinical pharmacology studies of LIVRQNac.

### 1.4.2 Nonclinical safety

The nonclinical safety of individually administered constituent AAs of AXA1125 has been well described in the literature. No observed adverse effect levels (NOAELs) for AAs have been established in nonclinical studies conducted under GLP conditions. The corresponding human equivalent dose (HED) values were calculated using a standard conversion factor (6.2 for rat; 1.1 for mini pig) and a 60 kg individual. At the highest planned dose of 67.8 g/day AXA1125, the doses of constituent AAs (ie, leucine 12 g, isoleucine 6 g, valine 6 g, arginine 18 g, glutamine 24 g, and Nac 1.8 g) were all below the corresponding HED values calculated from published NOAEL values as adjusted for a 60 kg individual. In these nonclinical studies, when AA constituents were administered individually at doses 2 to 4 times higher than the NOAELs, adverse events (AEs) (when present) were elevation in hepatic enzymes, hyperlipidemia, reduction in body weight, and decrease in hematocrit. All of these changes were generally mild, transient, and monitorable. Additional information on the nonclinical safety of AXA1125 can be found in the current version of the IB.

## 1.5 Clinical Experience

### 1.5.1 Clinical safety data based on published literature

The constituent AAs of AXA1125 have been administered to a broad spectrum of patient populations (pediatric to geriatric) with multiple disease states for extended durations (eg, 4 weeks to 3 years) and at doses comparable to or above those proposed for this study. Irrespective of subject age or disease conditions, AAs dosed at or above the proposed levels of Study AXA1125-101 were found to be safe and well tolerated. Additionally, the daily amounts of L-Leucine, L-Isoleucine, L-Valine,

L-Glutamine, and L-Arginine contained in the approved and marketed products (HepatAmine® United States Prescribing Information [USPI], Plenamine™ USPI, ENDARI™ USPI, and R-Gene® 10 USPI) are higher than the amounts of the corresponding AAs in AXA1125 at the top dose of 67.8 g/day AXA1125 proposed in this study.

### 1.5.2 Clinical data for AXA1125

#### 1.5.2.1 Clinical safety data for AXA1125

To date (safety data cut-off date: 26 August 2021), the safety of AXA1125 has been evaluated in

3 completed and 3 ongoing studies, in which a total of 111 subjects received at least 1 dose of

AXA1125 and up to 16 weeks of AXA1125. The evaluated populations included 43 healthy subjects

(Studies AXA1125-001, in which subjects received up to 6 weeks of AXA1125; AXA1125-004; and

AXA1125-005) and 61 subjects with NAFLD with and without type 2 diabetes mellitus (T2DM)

(Studies AXA1125-002 and AXA1125-003) and have included evaluation of up to 45.2 g/day AXA1125 for 16 weeks and 67.8 g/day AXA1125 for 12 weeks. A study (AXA1125-101) in subjects with liver biopsy-confirmed NASH and fibrosis is ongoing; no information on treatment-emergent adverse events (TEAEs) is available.

Other data supporting the initiation of this pilot study include a comprehensive compilation of literature on the safety of the constituent AAs within AXA1125, supporting their safety, tolerability, and effects on the mitochondrial dysfunction in NASH, and are available in the current version of the IB.

In healthy individuals (Studies AXA1125-001, AXA1125-004, and AXA1125-005), mild gastrointestinal symptoms were reported by 3 of the 10 subjects who received AXA1125 22.6 g 3 times daily (TID) for 6 weeks; these were considered related to study drug and led to dose reductions for 2 subjects. All events resolved, and there were no other safety findings. There were no dose-related trends in these product-emergent AEs (PEAEs). Transient increases in creatinine observed in 1 subject over multiple-dose periods in the single dose Study AXA1125-004 have not been observed in other, multiple-dose studies (up to 16 weeks of daily dosing), including in subjects with diabetes who may be more prone to renal injury.

Study AXA1125-002 was a 12-week, open-label, exploratory study of the safety and tolerability of

AXA1125 and its effect on liver structure and function for 12 weeks in subjects with NAFLD and T2DM. Daily administration of AXA1125 22.6 g TID (67.8 g/day) up to 12 weeks in subjects with NAFLD and T2DM was safe and generally well tolerated. Study AXA1125-003 was a 16-week, single-blind, randomized, placebo-controlled food study of the safety and tolerability of AXA1125 relative to placebo in subjects with NAFLD with and without T2DM. Daily administration of AXA1125 22.6 g twice daily (BID) (45.2 g/day) up to 16 weeks was safe and generally well tolerated.

In these subjects with NAFLD with and without T2DM, the majority of PEAEs were mild or moderate

in severity and unrelated to study drug, and few PEAEs were serious or led to study drug discontinuation. Across studies, the most common PEAEs were gastrointestinal including diarrhea, abdominal pain or distension, nausea, or headache. In Study AXA1125-002, no (0%) subjects who received AXA1125 5.65 g TID and 25% subjects who received AXA1125 22.6 g TID experienced diarrhea, although only 4 subjects were included in the former group and so results should be interpreted with caution. Of subjects with gastrointestinal PEAEs, only 2 subjects (1 subject with diarrhea and nausea in Study AXA1125-002 and 1 subject with upper abdominal pain in Study AXA1125-003) discontinued from the study. The remaining events were generally self-resolving; ie, intervention with anti-emetic or antidiarrheal agents was not required for these generally mild to moderate and transient gastrointestinal AEs. There were no reports of suspected AEs

based on interactions with concomitant therapies in either Study AXA1125-002 or Study AXA1125-003.

An analysis of selected gastrointestinal PEAEs in subjects who received AXA1125 in Study AXA1125-003 showed that the median time to onset ranged from 12.5 (for diarrhea and abdominal pain) to 25.5 days (for nausea) after the start of study drug administration, with a median duration ranging from 6.0 days (nausea) to 20.0 days (abdominal pain combined term). No subject in this study required treatment for these gastrointestinal PEAEs. Although no formal cross-study comparison was performed, the frequency of PEAEs in the gastrointestinal system organ class did not appear to be substantially different when comparing the dose of 67.8 g/day AXA1125 for 12 weeks (Study AXA1125-002) or 45.2 g/day AXA1125 for 16 weeks (Study AXA1125-003).

With the exception of a few laboratory and vital sign findings not considered related to study drug, there were no clinically significant abnormalities in physical examination, vital signs, clinical chemistries, or electrocardiogram assessments over up to 16 weeks of study drug administration.

The study data are consistent with the published literature on AXA1125 constituent AAs, which have demonstrated the safety and tolerability of these AAs both individually and in varying combinations at doses similar to or higher than the highest doses tested for AXA1125.

Overall, the safety and tolerability profile with AXA1125 at single doses up to 45.2 g in healthy subjects and at multiple doses of 45.2 g/day for 16 weeks and 67.8 g/day for 12 weeks in NAFLD subjects with and without T2DM is supportive of the investigation of AXA1125 in future clinical studies, including studies in subjects with PASC and fatigue.

Further clinical study details are summarized in the current version of the IB.

#### 1.5.2.2 Clinical pharmacokinetic data for AXA1125

Based on clinical experience from completed (Studies AXA1125-001, AXA1125-002, and AXA1125-003) and ongoing (Studies AXA1125-004 and AXA1125-005) studies, the salient pharmacokinetic (PK) features of AXA1125 include the following:

- Based on preliminary PK data in healthy subjects in ongoing Study AXA1125-004, the area under the plasma concentration-time curve from time 0 to the last quantifiable concentration (AUClast) and maximum plasma concentration (Cmax) for AXA1125-dosed AAs increased with increasing dose (5.7 to 45.2 g) except for glutamine Cmax.
- Dose proportionality was not demonstrated for AUClast for any of the AXA1125-dosed AAs based on statistical analysis; however, only minor deviations from dose proportionality (ie, slope close to 1 for most AXA1125-dosed AAs) were noted for AUClast for all AAs. A 7.9-fold increase in dose (5.7 to 45.2 g) was associated with a 7.95- to 14.24-fold increase in AUClast (ie, greater than proportional to the increase in dose) for all dosed AAs except glutamine. A 7.9-fold increase in dose was associated with a 6.22-fold increase in AUClast (ie, less than proportional to the increase in dose) for glutamine.
- Dose proportionality was not demonstrated for Cmax for any of the AXA1125-dosed AAs. A 7.9-fold increase in dose was associated with a 2.58- to 6.78-fold increase in Cmax (ie, less than proportional to the increase in dose) for all dosed AAs.
- Based on these observations, doses up to 33.9 g BID (67.8 g/day AXA1125) are expected to deliver key AAs in the systemic circulation in the linear area under the plasma concentration-time curve (AUC) range.
- Based on preliminary PK data in healthy subjects in ongoing Study AXA1125-005, systemic exposure (AUClast and Cmax) for most AXA1125-dosed AAs was similar following administration of AXA1125 22.6 g alone or with a low-fat breakfast. Based on these data and physicochemical properties of AXA1125 AAs, similar effects are anticipated at the 33.9 g dose. Therefore, AXA1125 can be administered with or without food.
- Limited PK data in the form of fasted predose plasma concentrations up to 16 weeks are available in subjects with NAFLD. Administration of AXA1125 22.6 g BID (45.2 g/day AXA1125) for 16 weeks or 22.6 g TID (67.8 g/day AXA1125) for 12 weeks did not meaningfully change the mean basal (fasted) AA plasma concentrations of AXA1125-dosed AAs, as compared to Baseline (Day 1) levels (ie, no accumulation was observed).
- Based on absorption, distribution, metabolism, and excretion properties of AAs; clinically relevant drug-drug interactions (DDIs) involving AXA1125 AAs; and co-administered drugs are unlikely at doses up to 33.9 g BID. Specifically, AXA1125 was not an in vitro inducer of CYP1A2, CYP2B6 and CYP3A4 mRNA at the concentrations tested in human hepatocytes and therefore DDI with oral contraceptives is unlikely.
- The kidneys play a pivotal role in the metabolism, elimination, and reabsorption of AAs. Patients with Stage 3 chronic kidney disease (ie, estimated glomerular filtration rate (eGFR)=30 to 59 mL/min/1.73 m2) are advised to consume a low protein (0.55 to 0.90 g/kg/day) diet (National Kidney Foundation Guidance 2019). Since consumption of AXA1125 (67.8 g/day, equivalent to 0.85 g/kg/day of protein) may potentially exceed the recommended protein intake for individuals with Stage 3 chronic kidney disease, a conservative approach will be taken, and an exclusionary cut-off of eGFR <60 mL/min/1.73 m2 is proposed for clinical studies until further data are available. Study AXA1125-002 and Study AXA1125-003 included subjects with mild renal impairment (eGFR ≥60 mL/min/1.73 m^2^). No clinically relevant changes in blood urea nitrogen, serum creatinine, or eGFR were reported in these studies. Effects of renal impairment on the PK of AXA1125-dosed AAs will be evaluated during the clinical development of AXA1125.
- Effects of hepatic impairment will be evaluated during clinical development of AXA1125. Therefore, subjects with cirrhosis or other meaningful disease of the liver will be excluded from clinical studies in subjects with NASH/PASC until these data are available.

#### 1.5.2.3 Clinical efficacy data for AXA1125

1.5.2.3.1 Studies in NASH

Study AXA1125-002 was a 12-week, open-label, exploratory study of the safety and tolerability of

AXA1125 and the effect on liver structure and function for 12 weeks in subjects with NAFLD and

T2DM. Daily administration of AXA1125 22.6 g TID (67.8 g/day) up to 12 weeks in subjects with NAFLD and T2DM was safe and generally well tolerated. The effects of AXA1125 on key pathways implicated in the pathogenesis of NASH were explored in this open-label study. Lipotoxicity and insulin resistance are considered metabolic drivers of NAFLD. Administration of AXA1125 22.6 g TID up to 12 weeks in subjects with NAFLD and T2DM was associated with a decrease in mean liver fat content and from 18.0% at baseline to 13.4% at Week 12. Homeostatic Model Assessment of Insulin Resistance (HOMA-IR) also decreased from 7.4 at baseline to 4.9 at Week 12. In addition, there was a decrease in mean alanine aminotransferase (ALT) levels, as well as other blood biomarkers of inflammation, apoptosis, and fibrosis. The mean ALT was 47.3 U/L at baseline and decreased to 32.1 U/L (-10.2% relative change), and N-terminal type III collagen pro-peptide (Pro-C3), a measure of fibrogenesis, decreased from 21.6 ng/mL at baseline to 16.9 ng/mL (-19% relative change).

Overall, results from this pilot open-label food study showed directional consistency of change across several structural and functional biomarkers associated with liver health after

AXA1125 administration and provided support for a larger, randomized, placebo-controlled study of AXA1125.

Study AXA1125-003 was a 16-week, single-blind, randomized, placebo-controlled food study of the safety and tolerability of AXA1125 relative to placebo in subjects with NAFLD with and without T2DM. Daily administration of AXA1125 22.6 g BID (45.2 g/day) up to 16 weeks was safe and generally well tolerated. A larger mean absolute and relative reduction from baseline in liver fat by magnetic resonance imaging-proton density fat fraction (MRI-PDFF) was observed at Week 16 with AXA1125 compared with placebo. The relative reduction in MRI-PDFF was -5.74% for those subjects who received placebo and -22.8% for those who received AXA1125. At Week 16, a relative

reduction in liver fat by MRI-PDFF by ≥30% from baseline was observed in a numerically higher percentage of subjects who received AXA1125 (10/26 subjects, 38.5%) compared with placebo (1/12 subjects, 8.3%). This is considered to be a clinically relevant threshold as PDFF has been correlated with the NAFLD activity score (Jayakumar 2019, Caussy 2020). Reductions in MRI-PDFF have been associated with histologic improvement, including reductions in inflammation and ballooning, in NASH studies using obeticholic acid (Loomba 2020) and ezetimibe (Patel 2016). Improvements in measurements of insulin resistance, such as HOMA-IR and glycated hemoglobin (HbA1c), were also seen more consistently in the AXA1125 group. HOMA-IR decreased -4.369 in those who received AXA1125 and increased 0.720 in those who received placebo. HbA1c decreased, with an absolute change in HbA1c of -0.30 in the AXA1125 group and -0.17 in the placebo group.

As a measure of hepatic injury, the relative reduction in ALT from baseline was greater in subjects who received AXA1125 compared with placebo at Week 16. The ALT reduction was -14.4 U/L (-21.86% relative change) in the AXA1125 group and -8.9 U/L (-7.2% relative change) in the placebo group. A numerically higher percentage of subjects in the AXA1125 group had an absolute reduction

in ALT of ≥17 U/L from baseline (10/26 subjects [38.5%] in the AXA1125 group compared to 3/12 subjects [25%] in the placebo group]), which is a threshold that has been associated with 2-point improvement in NAFLD activity score on biopsy (Loomba 2019).

Biomarkers of fibrosis were shown to be reduced by the administration of AXA1125. At Week 16, greater absolute and relative reductions in Pro-C3 were noted in the AXA1125 group compared with placebo.

Of note, changes in liver structure, metabolic effects, and fibroinflammatory biomarkers were numerically larger in the subgroup of subjects with T2DM, which is a clinically important subgroup of subjects with NASH. In the T2DM subgroup, there were decreases in HOMA-IR, HbA1c, fasting insulin, and insulin AUC (0-120 minutes) following an oral glucose challenge that were more pronounced in the AXA1125 group than those observed in the placebo group.

1.5.2.3.2 Studies in PASC

No clinical studies of efficacy have been conducted in subjects with PASC. An interim look at the data from Cohort A indicated no association between baseline fatigue score and baseline PCr. Additionally, change in fatigue score did not correlate with change in PCr recovery rate utilizing bimodal scoring at Week 4. However, there was a statistically significant improvement in fatigue using CFQ-11 when assessed prior to 6MWT. Accordingly, CFQ-11 was determined to be an appropriate primary endpoint for the assessment of fatigue in Cohort B which includes patients who were considered screen-failures in Cohort A due to their PCr recovery rate constant (< 50 seconds), provided their CFQ-11 score is _≥8_ and they meet all the eligibility criteria.

## 1.6 Study Rationale

PASC is a chronic, multi-organ disease predominantly characterized by fatigue and muscle weakness (Lopez-Leon 2021) potentially leading to significant impacts on individuals’ QoL, as well as their ability to be productive members of society insofar as it affects ability to work. The fatigue symptoms that patients with PASC experience is similar to chronic fatigue syndrome (Komaroff and Lipkin 2021).

AAs are known to catalyze a broad range of functions, including cellular metabolism, anti-oxidative signaling, differentiation, and cellular homeostasis, among others, across different organ systems and are therefore positioned to impact multiple pathophysiologies in this complex, systemic disease. Supplementation of AAs as single agents or high-order combinations has been reported to improve fatigue and muscle weakness (Wolfe 2017) and is hypothesized to improve PASC-fatigue via modulation of mitochondrial metabolism, redox imbalance, inflammation, and vascular insufficiency. AXA1125 (33.9 g BID), an orally active mixture of AAs, has the potential to alleviate fatigue in subjects with PASC compared to placebo. Results from previous studies have shown changes in bioenergetic state of tissue with administration of AXA1125 (Jason et al 2011).

To assess fatigue, this study utilizes the 6-minute walk test (6MWT) and Chalder Fatigue scale, which have been successfully validated and used in previous studies on chronic fatigue syndrome (Mantha et al 2020).

This is a pilot study, and the primary objective is to assess the efficacy and mechanism of action of AXA1125.

An interim look at the data from Cohort A indicated no association between Baseline fatigue score and Baseline PCr. Additionally, statistically significant improvement in fatigue scores using CFQ-11 prior to 6MWT did not correlate with changes in PCr recovery rate utilizing bimodal scoring at Week 4. Accordingly, CFQ-11 was determined to be appropriate primary end-point for the assessment of fatigue in Cohort B.

Moreover, in order to assess the relationship between AXA1125 and functional status, and to obtain valuable information on understanding the relationship between PCr and CFQ-11 results and

AXA1125 responses, those subjects who were considered screen-failures in Cohort A, due to their PCr recovery rate constant (<50 seconds) provided their CFQ-11 Total score is _≥8_ and they meet all the eligibility criteria for the study, will be included in a Cohort B to receive treatment for 4 weeks.

The efficacy endpoints for Cohort B include the change from Baseline at Week 4 in subjects’ fatigue score as assessed by CFQ-11 before 6MWT, which will be evaluated at Screening, Baseline, and End of Treatment or Early-Term. CFQ-11 will also be assessed at Day 14 (without 6MWT). Additional end-points for assessment of functional status, safety, and tolerability will be collected.

## 1.7 Benefit-Risk Assessment

PASC is a serious disease, potentially leading to significant impacts on individuals’ QoL, as well as their ability to be productive members of society insofar as it affects ability to work.

Improvements in mitochondrial bioenergetics have been observed in in vitro studies. The proposed dose is well supported by published literature of its constituents, including the clinical and nonclinical safety of its constituent AAs, and the clinical safety data available for AXA1125 across 3 completed studies and 3 ongoing studies for which preliminary data are available for 2 of the studies. Historical data have amply demonstrated the safety and tolerability of AAs, including in mixtures containing multiple AAs that have previously been approved as drugs. In cases where AEs were reported in past studies, they tended to be mild and transient gastrointestinal AEs, which were monitorable and generally clinically nonsignificant. AEs observed in short-term studies of AXA1125 have also shown a pattern of mild and transient gastrointestinal AEs. Thus, the in vitro findings and safety data for AXA1125 constituent AAs support a positive benefit-risk assessment for the use of AXA1125 in patients with PASC.

The safety and efficacy of AXA1125 has been evaluated in over 100 subjects as an amino acid food product. Studies in healthy volunteers and subjects with NASH are ongoing. AXA1125 has been found to be safe and well tolerated in doses up to 45.2 g/day AXA1125 for 16 weeks or 67.8 g/day AXA1125 for up to 12 weeks. No serious or related adverse reactions were identified by Axcella. Risks to subjects will be minimized by adherence to the eligibility criteria, close clinical monitoring, and monitoring of AEs.

To date, the cumulative data support the selection of doses of AXA1125 up to 67.8 g/day, which provides an optimal benefit-risk profile for evaluation of efficacy and safety.

Thus, the clinical efficacy and safety data for AXA1125 constituent AAs support a positive benefit-risk assessment for the use of AXA1125 in patients with PASC.

# 2 OBJECTIVES AND ENDPOINTS

## 2.1 Study Objectives – Cohort A

### 2.1.1 Primary objective

The primary objective is to:

• Assess the impact of AXA1125 on muscle function (metabolism) following exercise

### 2.1.2 Secondary objective

The secondary objectives are to:

- Assess the relationship between AXA1125 and functional status
- Assess the safety and tolerability of AXA1125

### 2.1.3 Exploratory objectives

The exploratory objectives are to:

- Obtain additional insights into the mechanism of action of AXA1125
- Obtain baseline AA profile in subjects with PASC

## 2.2 Study Objectives – Cohort B

- Assess the relationship between AXA1125 and functional status
- Assess the safety and tolerability of AXA1125

## 2.3 Study Endpoints

### 2.3.1 Efficacy endpoints – Cohort A

#### 2.3.1.1 Primary efficacy endpoint

The primary efficacy endpoint is:

• The mean change from baseline at Week 4 in the phosphocreatine (PCr) recovery rate following moderate exercise, as assessed by 31P-magnetic resonance spectroscopy (MRS)

#### 2.3.1.2 Secondary efficacy endpoints

The secondary efficacy endpoints are:

- Absolute and relative change from baseline in PCr recovery rate as assessed by phosphorus magnetic resonance spectroscopy (31P-MRS) at Week 4
- The proportion of subjects with improvement in PCr recovery rate at Week 4
- Absolute and relative change from baseline in serum lactate level after a 6MWT at Week 4
- The proportion of subjects with serum lactate level ≤3 mmol/L after a 6MWT at Week 4
- The proportion of subjects with a decrease in venous serum lactate level from baseline after a 6MWT at Week 4
- Change from baseline in distance traveled during a 6MWT at Week 4
- Change from baseline in subjects’ fatigue score as assessed by Chalder Fatigue Questionnaire (CFQ)-11 (by Bimodal Scoring) before and after a 6MWT at Week 4 and at Day 14 (without 6MWT)
- The proportion of subjects with an improvement in fatigue score as assessed by CFQ-11 before and after a 6MWT at Week 4

#### 2.3.1.3 Exploratory efficacy endpoints

The exploratory efficacy endpoints are:

- Change from baseline in circulating mitochondrial peptides (eg, Mots-C), metabolomics, proteomics; plasma biomarkers of inflammation, adhesion markers, muscle injury (eg, troponins, creatine kinase, fibroblast growth factor-21) biomarkers, and mitochondrial function/metabolism (~1.5 mL total); nitric oxide biology; immune profiling, and metabolism/phenotypic extracellular acidification rate and oxygen consumption rate at Week 4
- The mean change from baseline in energetically active metabolites measured using proton magnetic resonance spectroscopy (1H-MRS; ie, creatine, intramyocellular lipids, acetyl-carnitine, and carnosine) at Week 4
- Change from baseline in predose plasma concentrations of AAs at Week 4 (see Laboratory Manual for details)
- The mean change from baseline in minimal intramuscular pH after exercise, initial PCr recovery rate, adenosine diphosphate (ADP) concentration at the end of exercise, maximal mitochondrial capacity, and other parameters measured using dynamic 31P-MRS at Week 4

### 2.3.2 Efficacy endpoints - Cohort B

- Change from baseline in subjects’ fatigue score as assessed by Chalder Fatigue Questionnaire (CFQ)-11 (by Bimodal Scoring) before a 6MWT at Week 4, and at Day 14 (without 6MWT)
- The proportion of subjects with an improvement in fatigue score as assessed by CFQ-11 before a 6MWT at Week 4
- Change from baseline in distance traveled during a 6MWT at Week 4
- The proportion of subjects with an improvement in distance traveled as assessed by 6MWT at Week 4

### 2.3.3 Safety and tolerability endpoints - Cohort A and B

The safety and tolerability endpoints are:

- AEs and serious adverse events (SAEs)
- Physical examination findings, including vital signs (siting systolic and diastolic blood pressure, heart rate, respiratory rate, body temperature, resting O_2_ saturation) and body weight
- Change in clinical laboratory assessments, including chemistry, hematology, and urinalysis

# 3 STUDY DESIGN

## 3.1 Overall Study Design

###### Cohort A

This is a minimum of one center, randomized, double-blind, placebo-controlled, pilot clinical study in the United Kingdom (Figure 1). The study will evaluate the efficacy and safety of AXA1125 in subjects with fatigue-predominant PASC (>12 weeks after initial infection).

The total study duration for each subject will be approximately 9 weeks. This study will comprise a Screening Period of up to 4 weeks, a Treatment Period of up to 4 weeks, and a Follow-up Period of 1 week.

After obtaining informed consent, subjects will be screened, and approximately 40 eligible subjects

(approximately 20 subjects per arm) will be randomized in a 1:1 ratio to receive BID oral administration of 33.9 g AXA1125 or a placebo. Doses will be self-administered on Days 1 to 28, inclusive. Subjects will have clinic visits on Day 1 and Day 28, as well as telephone visits on Day 14 and 1 week after completion of study product administration. Note: Doses should be self-administered at the clinic visit on Day 1 and Day 28 or End of Treatment (EOT)/Early Termination (ET).

The primary efficacy endpoint is the mean change from baseline at Week 4 in the PCr recovery rate following moderate exercise, as assessed by 31P-MRS, which will be evaluated at Screening and EOT/ET (Visit 4). Additional endpoints for assessment of muscle function, safety, tolerability, and exploratory efficacy endpoints will be assessed during the study

Details of randomization will be provided in the Randomization Plan and Interactive Response Technology (IRT) system.

Figure 1 Study Schematic - Cohort A


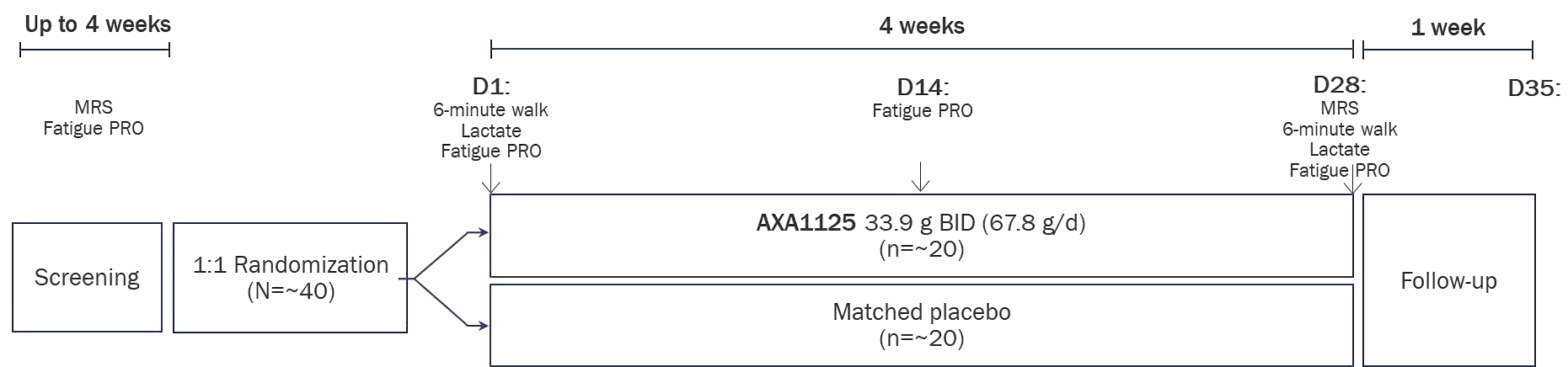


BID=twice daily; D=Day; MRS=magnetic resonance spectroscopy; PRO=patient-reported outcomes.

###### Cohort B

An additional cohort (approximately 10 subjects) will be added to the study which will include subjects who were considered screen-failures for Cohort A due to their PCr recovery rate constant (< 50 seconds) provided their CFQ-11 Total Score is _≥_8 and meet the rest of the eligibility criteria.

The total study duration for each subject in Cohort B will be approximately 6 weeks, which will comprise a Screening Visit (1 week), Baseline Visit, a Treatment Period of 4 weeks, and a Follow-up Period of 1 week.

After obtaining informed consent, subjects will be re-screened and approximately 10 eligible subjects will be randomized in a 1:1 ratio (double-blind, placebo-controlled) to receive either twice daily (BID) oral administration of 33.9 g AXA1125 or a placebo.

Doses will be self-administered on Days 1 to 28, inclusive. Subjects will have clinic visits on Days 1, and 28, as well as telephone visits on Day 14, and 1 week after completion of study product administration.

The efficacy endpoints for Cohort B include the change from baseline at Week 4 in subjects’ fatigue score, as assessed by Chalder Fatigue Questionnaire (CFQ)-11 (by Bimodal Scoring) before 6 MWT, which will be evaluated at Screening, Baseline, and End of Treatment or Early Termination. CFQ-11 will also be assessed at Day 14 (without 6MWT). Additional endpoints for assessment of functional status, safety, and tolerability will be assessed. Details of randomization will be provided in the Randomization Plan and Interactive Response Technology (IRT) system.

Figure 2 Study Schematic - Cohort B


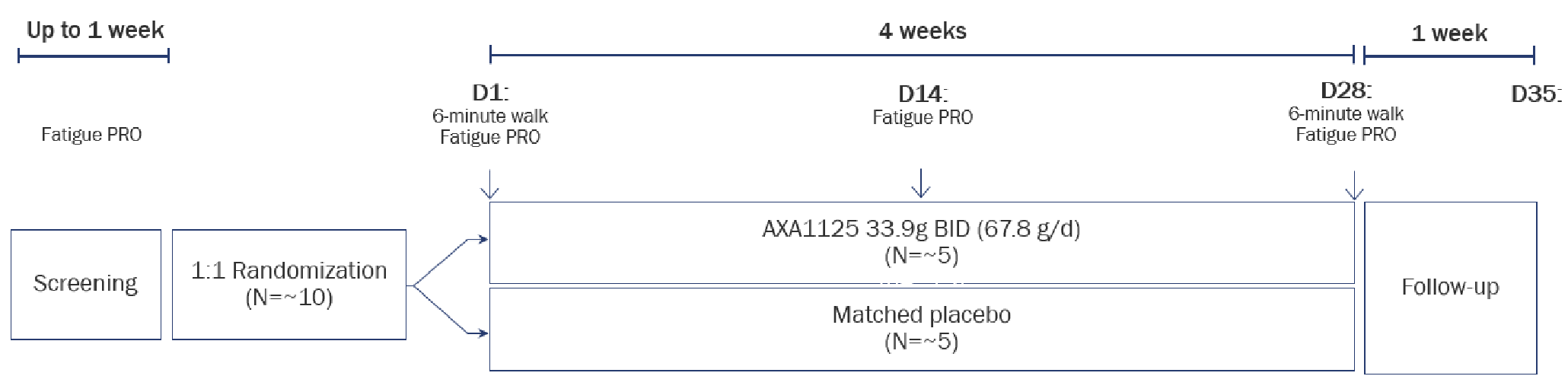


BID=twice daily; D=Day; PRO=patient-reported outcomes.

## 3.2 Scientific Rationale for Study Design

**Cohort A**

This pilot study is designed to assess AXA1125 dosed for up to 4 weeks in subjects with PASC. The primary objective is to assess the impact AXA1125 on muscle function (metabolism) following exercise. Key secondary objectives include assessment of the relationship between AXA1125 and functional status and assessment of the safety and tolerability of AXA1125 in subjects with PASC. It is anticipated that the administration of AXA1125 for up to 4 weeks in subjects with PASC will have an impact on muscle bioenergetics and consequently muscle function. 31P-MRS, 6MWT, and CFQ-11 are utilized as appropriate scales for the assessment of the study endpoints. Accordingly, the mean change from baseline at Week 4 in PCr recovery rate following moderate exercise, as assessed by 31P-MRS, was chosen as the primary endpoint for the study. With relatively rapid time resolution (<8 seconds) and certain assumptions, a range of bioenergetic refluxes will be determined from the metabolic changes. In particular, a functional estimate of mitochondrial oxidative capacity can be calculated from the kinetics of PCr recovery.

Additional biomarkers will be evaluated as exploratory endpoints to provide additional information about the full mechanism of action of AXA1125 and to assist in the design of the future development program.

A sample size of approximately 40 subjects (20 subjects per arm) will be enrolled in the study. In order to detect a meaningful treatment difference of 10 second improvement, approximately 32 subjects will provide 80% power at a 2-sided, 5% significance level, in addition to approximately 20% dropout rate.

This sample size will also allow for a general assessment of safety and tolerability of AXA1125 in this population. **Cohort B**

This Cohort is designed to assess AXA1125 dosed for up to 4 weeks in approximately 10 additional subjects with PASC, including subjects who were considered screen-failures due to their PCr recovery rate constant (< 50 seconds), provided their CFQ-11 score is ≥8 and they meet all the eligibility criteria for the study.

The key objectives for Cohort B include assessment of the relationship between AXA1125 and functional status, and assessment of safety and tolerability of AXA1125 in subjects with PASC.

Additionally, this Cohort is anticipated to provide valuable information on understanding the relationship between Baseline PCr and CFQ-11 results and AXA1125 responses.

CFQ-11, and 6MWT are utilized as appropriate scales for the assessments of this cohort’s endpoints. As an interim look at the data from Cohort A indicated that there was a statistically significant improvement in Fatigue using CFQ-11 when assessed prior to 6MWT.

A sample size of approximately 10 subjects will be enrolled, randomized in a 1:1 ratio to receive either twice daily (BID) oral administration of 33.9 g AXA1125 or a placebo.

This sample size will also allow for a general assessment of safety and tolerability of AXA1125 in this population.

## 3.3 Justification of Dose and Dosing Regimen

Dose selection was based on the following considerations to prior clinical experience with AXA1125, AA modality, formulation feasibility, subject compliance, and PK.

- The nonclinical safety of individually administered constituent AAs of AXA1125 has been well described in the literature. At the highest planned dose of 67.8 g/day AXA1125 (33.9 g BID), the doses of constituent AAs (ie, leucine 12 g, isoleucine 6 g, valine 6 g, arginine 18 g, glutamine 24 g, and Nac 1.8 g) are all below the corresponding HED values, calculated from published NOAEL values as adjusted for a 60-kg individual. In these nonclinical studies, when AA constituents were administered individually at doses 2 to 4 times higher than the NOAELs, AEs (when present) were generally mild, transient, and monitorable (see Section 1.4.2).
- The safety and tolerability of the highest dose of 67.8 g/day is supported by the Studies AXA1125-001 and AXA1125-002. Even at the highest dose of 67.8 g/day, AEs observed to date have been primarily mild to moderate gastrointestinal findings (diarrhea, abdominal pain, or discomfort), which are typically self-limited (see Section 1.5.2.1).
- Based on PK observations, the doses up to 33.9 g BID (67.8 g/day) are expected to deliver key AAs in the systemic circulation in the linear AUC range. Similar to Study AXA1125-003, which showed encouraging biological activity, a BID regimen was chosen for Study AXA1125-101 to ensure optimal subject compliance during chronic dosing.
- AXA1125 can be administered with or without food based on the preliminary PK data.
- Daily administration of AXA1125 at 22.6 g TID (67.8 g/day) up to 12 weeks (Study AXA1125‑002) or 22.6 g BID (45.2 g/day) up to 16 weeks (Study AXA1125‑003) was associated with clinically relevant changes in biomarkers of metabolism and fibroinflammation (eg, MRI-PDFF, Pro-C3, and ALT) in subjects with NASH.
- Pharmaceutical properties were also considered. A single sachet is designed to deliver 11.3 g of AXA1125 AAs. The excipients and their respective concentrations were specifically chosen for AXA1125 and were designed to deliver a uniformly dispersed, orange-colored, orange-flavored liquid suspension upon constitution of up to 3 sachets (33.9 g of AXA1125 AAs) in ~6 fluid oz (~180 mL) of potable water. Specifically, the concentration of lecithin ensures sufficient wetting of the hydrophobic AAs (eg, leucine, isoleucine, and valine) to deliver a uniform dispersion of these ingredients in the 6 fluid oz dose volume. The concentration of xanthan gum has been titrated to the 6 fluid oz dose volume to create the required liquid suspension presentation. Finally, the citric acid and orange-flavor concentrations were chosen to deliver a pleasant orange-colored, orange-flavored drink in the 6 fluid oz dose volume. These excipients in combination with other nonactive ingredients in the drug product enable the delivery of positive organoleptic properties (eg, acceptable mouthfeel and taste) to subjects, potentially aiding in subject dose administration compliance. Therefore, doses >33.9 g (ie, 45.2 g) warrant a higher volume (>6 fluid oz) of potable water for constitution, which could negatively impact subject compliance, palatability, and tolerability. Also, to maintain study blinding, AXA1125 or placebo will be delivered in the same 6 fluid oz dose volume. Using a higher volume for the active arm but not the placebo arm could potentially unblind the study. Similarly, the use of 33.9 g TID also poses blinding and compliance issues.
- Unlike 2 doses studied in NASH, a single dose of 33.9 g BID is proposed for the PASC study. While the ongoing NASH study provides dose-response information on several biomarkers, the use of a single dose (33.9 g BID) in PASC allows for maximizing Nac dose (1.2 g BID) and thereby maximizing intracellular glutathione pools that have been considered crucial for ameliorating COVID-19-related mitochondrial dysfunction. Furthermore, 10 g TID glutamine (30 g/day) supplementation in subjects with COVID-19 has been shown to shorten the length of hospital stay and reduce the need for intensive care unit treatment (Cengiz 2020). The 33.9 g BID AXA1125 dose also allows for maximizing glutamine dose at 12 g BID (24 g/day).

Overall, the available PK and PD data along with considerations related to formulation feasibility, available clinical safety data, biologically relevant concentrations, and literature support the selection of 33.9 g BID (67.8 g/day) as an adequate dose to evaluate the safety and tolerability of AXA1125 in subjects with PASC in Study AXA1125-201.

## 3.4 Study Duration

**Cohort A**

Subjects will participate for up to 9 weeks. The study will comprise a Screening Period of up to 4 weeks, a Treatment Period of 4 weeks, and a Follow-up Period of 1 week. **Cohort B**

The total study duration for each subject in Cohort B will be approximately 6 weeks, which will comprise a Screening Period (1 week), Baseline Visit, a Treatment Period of 4 weeks, and a Followup Period of 1 week.

# 4 STUDY POPULATION AND SELECTION

## 4.1 Inclusion Criteria

A subject must meet all of the following inclusion criteria to be considered for enrollment into this study:

###### Cohort A and B

1. A subject must be male or female 18 years of age or older, and under 65 years of age.
2. A subject must be able to provide written informed consent and be able to understand and willing to comply with specified requirements as stated in the protocol.
3. A subject must have had clinically suspected COVID-19 and a positive antibody test or a documented SARS-CoV-2 infection (a positive reverse transcription polymerase chain reaction test) at least12 weeks prior to Screening.

Note: Subjects who had a clinical diagnosis of COVID-19, but could not have a PCR test due to the need to self-isolate are allowed.

1. A subject must have fatigue-predominant PASC defined by:

###### Cohort A Only

• an elevated PCr recovery rate constant following moderate exercise on 31P-MRS (τ_PCr_ ≥50 seconds); and

Note: Documented 31P-MRS within 3 months of Screening and after 3 months of acute COVID-19 infection is acceptable. If a historical 31P-MRS has been used to determine eligibility, then 31P-MRS should be repeated within 1 month prior to Day 1 (ie, during the Screening Period) to confirm eligibility and establish baseline.

1. Other than PASC, a subject must be in good health without other significant medical or not well controlled medical or psychiatric conditions. Allowable conditions include mild hypertension,

dyslipidemia, pre-diabetes defined as HbA1c ≤ 6.0%, and/or asthma (mild and not requiring chronic daily treatment). Subjects who are treated for these conditions must be well controlled on a stable regimen (lifestyle modifications and/or medications), for at least 3 months prior to Screening and anticipate no significant alterations to these regimens for the duration of the study. However, doses of certain medications (eg, statins, antihypertensives) used to treat stable chronic conditions may be modified during the study for safety or tolerability issues, if needed at the discretion of the Principal Investigator (PI).

Note: A history of treated hepatitis C virus (HCV) infection is allowed, provided that a negative hepatitis C viral load has been documented at ≥12 weeks after the cessation of hepatitis C treatment. Historical medical record or testing to ensure a negative viral load may be permitted during the Screening Period. HCV ribonucleic acid will be tested in all subjects, including subjects with a history of treated HCV infection or with positive HCV antibody at Screening, provided that a negative hepatitis C viral load has not been documented at ≥12 weeks after the cessation of hepatitis C treatment.

1. A female subject must meet any one of the following criteria:
   1. Surgically sterile
   2. Postmenopausal with ≥12 months of amenorrhea without an alternate medical cause
   3. Follicle-stimulating hormone (FSH) level consistent with postmenopausal state if amenorrheic for <12 months or if <55 years of age
   4. If a subject is of childbearing potential, must have a negative serum pregnancy test during Screening and agree to abstain from sexual activity or agree to use a highly effective form of birth control (failure rate <1% per year) for the duration of the study and for at least 30 days after the last dose of study drug
2. A male subject must meet the following criteria:
   1. Male subjects with a partner who is a female of childbearing potential (FCBP), must agree to abstain from sexual activity or agree to use a highly effective form of birth control (failure rate <1% per year) for the duration of the study and for at least 90 days after the last dose of study drug. Note: Female partner use of a highly effective form of birth control (failure rate <1%) is acceptable for male subjects participating in the study.
   2. Male subjects capable of fathering a child must agree to refrain from sperm donation for the duration of the study and for at least 90 days after the last dose of study drug.
3. A subject must agree to refrain from physical activity outside of normal activities of daily living before Day 1 and for the duration of the study
4. A baseline CFQ-11 total fatigue score (by Bimodal Scoring) of ≥8.

## 4.2 Exclusion Criteria (Cohort A and B)

A subject will be excluded from the study if **any** of the following criteria are met:

1. Other than PASC, have an explanation for fatigue. This includes but is not limited to hypothyroidism; chronic cardiovascular, neurological, endocrinological, or peripheral vascular disease; history of major depression; clinically significant anemia; chronic liver disease; rheumatologic diseases requiring treatment with steroids or immunosuppressants; or other cause of neuromuscular disease such as muscular dystrophy.
2. Other than PASC, a history or presence of an uncontrolled, clinically significant disease including:
   - Type 1 or Type 2 diabetes mellitus
   - A medical condition that may interfere with absorption of the study drug, including but not limited to resection of any part of the gastrointestinal tract, gastric or intestinal bypass, any bariatric surgery, or inflammatory bowel disease
3. Meet any of the following during physical exam assessment:
   - Resting O_2_ saturation <95% on room air
   - Body mass index (BMI) of <18.5 or >35
   - Clinically significant abnormality on echocardiogram (to rule out abnormal cardiac function or elevated pulmonary artery pressure)
   - Is pregnant (if female) or lactating
4. Meet any of the following laboratory parameters:
   - Serum B-type natriuretic peptide (NT-proBNP) >400 pg/mL
   - Total bilirubin >1.3 mg/dL (>22.23 umol/L) or direct bilirubin >0.40 mg/dL (>6.84 umol/L) unless with a history of Gilbert’s syndrome
   - Aspartate aminotransferase (AST) or ALT >3 × upper limit of normal (ULN) AST >126 IU/L; ALT >135 IU/L
   - HbA1c >6.0%
   - Human immunodeficiency virus (HIV)-1 or -2 positive
   - eGFR <60 mL/min/1.73 m2 calculated using the Chronic Kidney Disease Epidemiology Collaboration equation
   - Positive for hepatitis B surface antigen (hBsAg), HCV antibody, or HIV antibody
5. Have a medical history that includes any of the following:
   - Non-invasive or invasive ventilatory support for COVID-19
   - Intensive care unit or other high dependency unit admission for COVID-19
   - Hospitalization for >1 week for COVID-19 without intubation
   - Planned or prior organ transplant
6. Treatment with drugs known to cause myopathy in the last 3 months. This includes glucocorticoids, antimalarials, colchicine, antiretroviral drugs, interferon alpha, penicillamine, immune checkpoint inhibitors, tumor necrosis factor inhibitors and statins, if there is a history of statin-induced muscle pain.

Note: The Medical Monitor should be consulted if there are questions regarding concomitant medications.

1. Unwilling or unable to stop the use of AA or protein supplements, carnitine, creatine, or Nac at the time of Screening until the end of study
2. A history of inborn errors of metabolism that may impact AA metabolism, including but not limited to urea cycle disorders
3. A contraindication for a safe magnetic resonance imaging scan, including implanted magnetic metal
4. Any history of illicit drug or alcohol abuse within 6 months prior to Screening.
5. Used an investigational drug, product, or device within 30 days or 5 half-lives (whichever is longer) before Screening, or is enrolled in another investigational drug, product, or device study within 30 days before Screening; Note: Enrollment in registration or observational studies is permitted.
6. A contraindication, sensitivity, or known allergy to any ingredient of AXA1125; or
7. Considered, in the opinion of the PI, to be a poor attendee, or for any reason is not able to comply with the study procedures due to reasons such as planned procedures, travel, etc, that would occur during the course of the study

## 4.3 Screen Failures and Rescreening (Cohort A and B)

Screen failures are subjects who do not fulfill the eligibility criteria for the study and therefore must not be assigned treatment or randomization. These subjects should have a reason for study withdrawal recorded as “eligibility criteria not fulfilled” (subject does not meet the required inclusion or meets any of the exclusion criteria). This reason for study withdrawal is only for screen failures. Subjects who fail screening will have the following information collected: demography, eligibility criteria including reason for screen failure, and AEs.

Screen failures for reasons other than use of prohibited medications(s) may be discussed with the Medical Monitor to determine if rescreening is appropriate. Subjects may be rescreened once with approval from the Medical Monitor; rescreening will require a new subject number.

Subjects who were considered screen-failures due to their PCr recovery rate constant (< 50 seconds) provided their CFQ-11 score is ≥ 8 and meet the rest of the eligibility criteria will be rescreened and included in Cohort B.

## 4.4 Subject Enrollment (Cohort A and B)

A subject will be considered for enrollment when the subject provides written informed consent, or reconsent as applicable for local policies and regulations, and when the subject has met all inclusion criteria and none of the exclusion criteria. Further information about the requirements for written informed consent is provided in Section 8.1.1.1.

A subject will be considered enrolled when he or she has met all eligibility criteria, has been selected to participate in the study, and has agreed to participate and has been randomized.

Approximately 40 subjects (Cohort A), and approximately10 subjects (Cohort B) are to be enrolled in this study.

## 4.5 Subject Withdrawal (Cohort A and B)

While subjects are not obligated to state the reason for withdrawal, the reasons for withdrawal or failure to provide a reason must be documented by the PI on the relevant electronic case report form (eCRF). Every effort should be made by the PI to follow subjects who withdraw from the study for at least 30 days.

An enrolled subject who withdraws consent or is withdrawn from the study per the PI’s discretion before completing all study activities per protocol will be considered an early termination.

### 4.5.1 Withdrawal from study participation

Participation in this study is voluntary, and a subject may withdraw consent and discontinue participation in this study at any time at his/her own request without prejudice or consequence. The PI may also, at his/her discretion, discontinue the study treatment and/or withdraw the subject from the study at any time (eg, safety, behavioral, or administrative reasons). If a subject withdraws from the study, they will be instructed to return all unused study drug to the study site or central location for study drug accountability. Samples not taken are to be destroyed, and the PI must document this in the site study records.

A subject may withdraw from study participation (study drug and study assessments) or be withdrawn from study participation by the PI for reasons including but not limited to any of the following:

- Subject’s stable chronic medical condition(s) deteriorates and/or clinically significant adjustments/titrations in their stably maintained medical regimen are made during the study;
- Subject independently withdraws consent from the study;
- Subject has 1 or more medical reasons considered clinically significant by the subject, PI, and/or Sponsor, which may include an intercurrent illness, condition, AE, and/or reasons unrelated to the study;
- Protection of the subject’s health or the integrity of the study;
- Subject failure to comply with study requirements (eg, failure to properly consume the study drug [<80% compliance] failure to comply with other protocol-specific assessments); or
- Subject is lost to follow-up (Section 4.5.4).

Early discontinuation of study drug alone is not a criterion for withdrawal of consent for participation in the study.

### 4.5.2 Withdrawal from study drug only

A subject may withdraw from study drug and remain in the study by continuing all study assessments for reasons, including but not limited to any of the following:

- If a subject experiences an SAE judged by the PI to be possibly, probably, or definitely related to the study drug, the subject may be discontinued from the study drug and will not restart treatment but may remain on study for continued follow-up until resolution of the SAE; the subject will continue his/her consent to participate in the study for follow-up only (eg, for continued collection of AE information);
- Development of an AE(s). AEs leading to discontinuation of the study drug should be followed until the AE is resolved;
- Inability to self-administer study drug orally. The subject will be discontinued from the study drug but may remain on study for continued follow-up;
- Protection of the subject’s health or the integrity of the study;
- Grade 3 or higher and related to study drug or Grade 4 or higher regardless of causality as defined by the Common Terminology Criteria for Adverse Events (CTCAE) version 5.0 (see Section 6.7.3)

### 4.5.3 Handling of discontinuation of study drug and study withdrawals

If the PI discontinues a subject from study drug due to development of an AE, every effort should be made to complete the ET procedures as outlined in the Schedule of Assessments (SOA;

APPENDIX 1; APPENDIX 2) as soon as possible after the last dose of study drug and to monitor the subject for resolution of the AE or for the development of new AEs for 4 weeks after the last dose of study drug.

If a subject withdraws consent or is terminated for any other reason, every effort should be made to complete the ET assessments as outlined in the SOA (APPENDIX 1; APPENDIX 2).

The reason for subject withdrawal must be documented in the eCRF.

Subjects who discontinue or withdraw from the study will not be replaced.

### 4.5.4 Lost to follow-up

A subject is considered lost to follow-up when he/she repeatedly fails to return to the site for scheduled visits and the site is unable to contact the subject.

If a subject fails to return to the site, the study site personnel must:

- Attempt to contact the subject and reschedule the missed visit as soon as possible.
- Counsel the subject on the importance of maintaining the assigned visit schedule.
- Ascertain whether the subject wishes to and should continue in the study.

Before a subject is considered lost to follow-up, the PI or designee must make 3 attempts to regain contact with the subject. These contact attempts should be documented in the subject’s source documents.

Should the subject continue to be unreachable, only then will he/she be considered to have withdrawn from the study with the primary reason of “Lost to follow-up”.

# 5 STUDY INTERVENTIONS (Cohort A and B)

## 5.1 Study Drug

### 5.1.1 AXA1125

AXA1125 is an orally active mixture of 5 specific AAs (leucine, isoleucine, valine, arginine, and glutamine) and Nac (an AA derivative). The AA composition within AXA1125 is described in Table 1.

###### Table 1 Amino Acid Composition Within AXA1125

| **Ingredient** | **Ingredient Function** | **Unit Dosage Dry Weight (g)** |
| --- | --- | --- |
| L-Leucine | Active | 2.000 |
| L-Isoleucine | Active | 1.000 |
| L-Valine | Active | 1.000 |
| L-Arginine HCl^1^ | Active | 3.628 |
| L-Glutamine | Active | 4.000 |
| N-acetyl-L-cysteine | Active | 0.300 |

HCl=hydrochloride

Note: Total may be >100% due to rounding off.

^1^ Arginine is sourced as arginine monohydrochloride; 3.628 g of L-Arginine HCl equals 3.001 g of L-Arginine.

The excipients within AXA1125 include citric acid, soybean lecithin, xanthan gum 180, sucralose, orange flavor, custard flavor, FD&C yellow no. 6, low-substituted hydroxypropyl cellulose NBD-022, colloidal silicon dioxide, magnesium stearate, and AAs without hydrochloride. The excipients and their respective concentrations were specifically chosen for AXA1125 and are designed to deliver a uniformly dispersed, orange-colored, and orange-flavored liquid suspension upon constitution with ~6 fluid oz of potable water.

The active pharmaceutical ingredients and excipients are compounded to produce a uniform dry powder blend for suspension and are provided as a unit dose sachet containing 11.3 g AAs. The container closure system for AXA1125 is a 5-layer

(paper/polyester/polyethylene/foil/polyethylene), heat-sealed, unit-dose, foil sachet. One end of the heat-sealed sachet is notched to provide an easy-tear opening for subjects.

Detailed information regarding AXA1125 formulation, constitution instructions, appearance, and storage requirements are described in the Pharmacy Manual.

### 5.1.2 Placebo

The placebo is formulated as a dry powder that has excipient and calorie content similar to AXA1125 (see Table 2). Excipient content that is dissimilar to AXA1125 is as follows: soybean lecithin has been removed because it is not required to ensure sufficient wetting of the excipients in the placebo formulation, sodium citrate dihydrate has been added and the citric acid amount reduced in order to match the pH of AXA1125, and maltodextrin and sunflower oil powder have been added as fillers. Each placebo and AXA1125 sachet contain approximately 76 kcal and provide approximately 456 kcal/day to subjects regardless of the treatment arm.

The placebo will be supplied to closely match the aroma, appearance, color, and taste profile of AXA1125 as powder in sachets. The total number of sachets, packaging, constitution, and administration of placebo mirrors AXA1125 to maintain double blinding during the study.

Calories are primarily delivered by a combination of maltodextrin (~50.4 kcal per sachet, or up to 302 kcal per day based on 3.78 kcal/g) and sunflower oil powder (~21.5 kcal per sachet, or up to 129 kcal per day based on 7.38 kcal/g). In Study AXA1125-003, the additional calories delivered to the subjects receiving placebo with similar composition did not result in an increase in weight, HbA1c, fasting glucose, or the AUC of glucose in an oral glucose tolerance test from baseline to Week 16 (see the current version of the IB); assessments of weight and glucose homeostasis are included in this study.

###### Table 2 Excipient Function Within Placebo

| **Ingredient** | **Ingredient Function** |
| --- | --- |
| Maltodextrin, NF | Filler |
| Sunflower oil powder | Filler |
| Citric acid, anhydrous | pH adjuster |
| Sodium citrate, dihydrate,^1^ NF | Buffer, pH adjuster |
| Xanthan gum 180 | Thickening agent |
| Sucralose, powder | Sweetener |
| Orange flavor, natural and WONF | Flavor |
| Custard flavor, natural and artificial | Flavor |
| FD&C yellow no. 6 | Color |
| Low-substituted hydroxypropyl cellulose NBD-022 | Disintegrant |
| Colloidal silicon dioxide | Disintegrant |
| Magnesium stearate | Lubricant |

FD&C = food, drug, and cosmetic; NBD = New Binder Disintegrant; NF = National Formulary; WONF = with other natural flavors.

^1^ The total citric acid amount is distributed between citric acid and sodium citrate to ensure target pH. Total daily amount of citric acid matches the total daily amount in AXA1125.

Detailed information regarding the placebo formulation, constitution instructions, appearance, and storage requirements are described in the Pharmacy Manual.

### 5.1.3 Study drug storage

Study drug should be stored at a controlled room temperature, preferably between 15°C to 25°C (59°F to 77°F) at the study site. Subjects will be instructed to maintain the study drug in its cartons and/or kits at ambient temperature in a cool and dry environment until consumption.

### 5.1.4 Dosage, dose titration, and administration

At each dose, the 3 sachets will be constituted in ~6 fluid oz (~180 mL) of potable water to form a uniformly dispersed suspension. The suspension is to be self-administered orally as an orange-colored, orange-flavored drink, with or without food. Administration around mealtimes is recommended to promote compliance with the BID regimen and should occur at least 4 hours apart. The full dose needs to be taken within 30 minutes from the constitution of the study drug.

Constitution instructions are provided in the Pharmacy Manual.

### 5.1.5 Missed doses

If a subject misses a dose, then dosing should resume as soon as practical, so that the 2 doses are taken at least 4 hours apart. Study drug can be administered with or without food. Administration around mealtimes is recommended to promote compliance with the BID regimen.

### 5.1.6 Blinding

The subject, investigators, study site personnel involved in direct care of the subject will remain blinded. Personnel involved in the laboratory and image analysis will also remain blinded to the treatment assignment.

The Sponsor study team members will remain blinded to the treatment assignments during the study. A Sponsor biostatistician will be unblinded to perform the interim analysis and data monitoring.

AXA1125 and placebo will be identical in terms of the physical primary and secondary packaging. The primary and secondary packaging labeling will also be coded to prevent unblinding. The container closure system for the placebo will be the same heat-sealed foil sachet used for AXA1125. Additionally, the study drug will be packaged with blinded identifiers and secondary packaging (cartons) will be weight distributed to maintain the study blind.

The total number of sachets (3 sachets per dose) will be matched across all arms. The placebo closely matches the aroma, appearance, color, and taste of AXA1125 and will be constituted in the same ~6 fluid oz (~180 mL) volume of potable water.

Further details are provided in the Pharmacy Manual.

### 5.1.7 Accountability

The PI, pharmacist, and/or designee must maintain records of the study drug’s delivery to the study site, the inventory at the study site, the use by each subject, and the return to the Sponsor, clinical research organization (CRO), warehouse/drug distribution center, or alternative disposal of unused study drug. Subjects will be instructed to return all unused study drug to the study site or central location for study drug accountability reviews at the visits specified in the SOA (APPENDIX 1; APPENDIX 2). As applicable, the Sponsor, CRO, or warehouse/drug distribution center will maintain records of study drug disposal.

The records will include dates, quantities, batch/serial numbers, expiry (‘use-by’) dates, and unique code numbers assigned to the study drug and subjects. The PI, pharmacist, and/or designee will maintain records that adequately document that the subjects were provided the doses specified by the protocol and reconcile all study drug received from the Sponsor or delegate. At the time of return to the Sponsor/CRO, the PI, pharmacist, and/or designee must verify via documentation that unused or partially used study drug supplies have been returned by the subject and that no remaining study drug is in the study site’s possession.

Further details are provided in the Pharmacy Manual.

## 5.2 Medications, Supplements, Treatments, and Procedures

### 5.2.1 Concomitant medications and/or supplements

All concomitant medications and/or supplements taken within 4 weeks of Screening will be recorded on the eCRF. Medications and supplements to be reported in the eCRF include all prescription medications, over-the-counter medications, and other nonprescription medications or supplements taken by a subject. For this study, a prescription medication is defined as a medication that can be prescribed only by a properly authorized/licensed clinician.

Other than medications or supplements related to the subject’s standard of care therapy, there are no study-specific concomitant medications or supplements required by this study.

A stable regimen for lifestyle modifications and medication use is allowed as described in Section 5.2.2.

Any medication or vaccine (including over-the-counter or prescription medicines, vitamins, and/or herbal supplements) that the subject received within 4 weeks of Screening or receives during the study must be recorded along with the following:

- Reason for use;
- Dates of administration, including start and end dates; and • Dosage information, including dose, route, and frequency.

If clinically significant changes in any concomitant medications occur or if prohibited medication usage is required during the study, the PI should discuss with the Medical Monitor and Sponsor whether the subjects should continue in the study or be withdrawn.

### 5.2.2 Allowed medications, treatments, and/or procedures

Receipt of the following medications, treatment, and/or procedures are allowed during a subject’s participation in the study:

• A stable regimen (lifestyle modifications and/or medications) per the PI’s judgement, for at least 3 months prior to and during Screening, is allowed for the treatment of conditions that are well controlled, including mild hypertension, dyslipidemia, asthma (mild or not requiring chronic daily treatment), and/or hypothyroidism.

Note: Doses of certain medications (eg, statins, antihypertensives) used to treat stable chronic conditions may be modified during the study for safety or tolerability issues, if needed at the discretion of the PI.

### 5.2.3 Prohibited medications, treatments, and/or procedures

In addition to the medications, supplements and/or activities that are listed in the exclusion criteria (Section 4.2), the following are prohibited while a subject is participating in this study:

- Any new medications, doses, or regimens for the treatment of uncontrolled conditions, including mild hypertension, dyslipidemia, and/or asthma (mild or not requiring chronic daily treatment);

Note: Doses of certain medications (eg, statins, antihypertensives) used to treat stable chronic conditions may be modified during the study for safety or tolerability issues, if needed, at the discretion of the PI.

- Drugs that are known to cause myopathy, as in Exclusion Criterion 6;
- AA or protein supplements, carnitine, creatine, or Nac;
- Use of an investigational drug, product, or device, or enrollment in another study of an investigational drug, product, or device;
- Surgical procedure which may interfere with absorption of the study drug, including but not limited to resection of any part of the gastrointestinal tract, gastric or intestinal bypass, any bariatric surgery, or inflammatory bowel disease; or
- Organ transplant.

If there are questions regarding concomitant medications, the Medical Monitor should be consulted.

Should circumstances require the use of prohibited medication(s), medical treatment(s), or procedure(s) during the study, the PI should discuss with the Medical Monitor and Sponsor and assess whether to discontinue the subject from study drug or from the study in order for the subject to receive any necessary and appropriate medical care.

Subjects who have medical treatment(s) or procedure(s), which would otherwise prohibit entry into the study planned during the duration of the study will be deemed ineligible at the Screening Visit.

## 5.3 Prevention of Pregnancy

Subjects who are FCBP or males with a partner who is a FCBP must use a highly effective form of birth control from the day of the first dose of study drug, throughout the study, and for 30 days (females) or 90 days (males) after the last dose of study drug.

Definition of Female of Childbearing Potential and of Fertile Men

A female is considered of childbearing potential (ie, fertile) following menarche and until becoming post-menopausal unless permanently sterile. Permanent sterilization methods include hysterectomy, bilateral salpingectomy, and bilateral oophorectomy.

A postmenopausal state is defined as no menses for 12 months without an alternative medical cause. A high FSH level in the postmenopausal range may be used to confirm a postmenopausal state in women not using hormonal contraception or hormonal replacement therapy. However, in the absence of 12 months of amenorrhea, a single FSH measurement is insufficient.

A man is considered fertile after puberty unless permanently sterile by bilateral orchidectomy Birth Control Methods Which May be Considered as Highly Effective

A highly effective method is one that can achieve a failure rate of <1% per year when used consistently and correctly. The acceptable birth control methods for female subjects are:

- Combined (estrogen and progesterone- [progestogen-] containing) hormonal contraception associated with inhibition of ovulation, administered by oral, intravaginal, or transdermal routes;
- Progesterone- (progestogen-) only hormonal contraception associated with inhibition of ovulation, administered by oral, injectable, or implantable routes;
- Intrauterine device;
- Intrauterine hormone-releasing system;
- Bilateral tube occlusion;
- Vasectomized partner (provided the partner is the sole sexual partner of the FCBP subject and that the vasectomized partner has received medical assessment of the surgical success); or
- Complete sexual abstinence (ie, refraining from heterosexual intercourse; the reliability of sexual abstinence should be evaluated in relation to the duration of this study and the preferred and usual lifestyle of the subject).
- Note: True abstinence, when in line with the preferred and usual lifestyle of the patient, is considered a highly effective method only if defined as refraining from heterosexual intercourse during the entire period of risk associated with the study drug treatment. Periodic abstinence (eg, calendar, ovulation, symptothermal, or post-ovulation methods) and withdrawal are not acceptable methods of contraception.

The acceptable birth control methods for male subjects are:

- Barrier contraception (eg, latex condom with spermicide, diaphragm with intravaginal spermicide, or cervical cap with spermicide);

Note: Female partner use of a highly effective form of birth control (failure rate <1% per year) is acceptable for male subjects participating in the study.

- Vasectomy with documented azoospermia; or
- Complete sexual abstinence (ie, refraining from heterosexual intercourse; the reliability of sexual abstinence should be evaluated in relation to the duration of this study and the preferred and usual lifestyle of the subject).

Note: True abstinence, when in line with the preferred and usual lifestyle of the patient, is considered a highly effective method only if defined as refraining from heterosexual intercourse during the entire period of risk associated with the study drug treatment. Periodic abstinence (eg, calendar, ovulation, symptothermal, or post-ovulation methods) and withdrawal are not acceptable methods of contraception.

In addition to using a highly effective form of birth control, males will not donate sperm from the day of the first dose of study drug to 90 days after the last dose of study drug.

## 5.4 Dietary Restrictions and Lifestyle Modifications

Throughout the study, subjects will consume the diet associated with their standard of care, maintaining a diet stable in calories and protein intake from Screening through the final study visit.

## 5.5 Other Restrictions

Smoking should be avoided.

Subjects are required to fast (water permitted) for a minimum of 10 hours prior to study drug dosing at Baseline (Visit 2) and EOT/ET (Visit 4) when blood samples will be assessed for biomarkers (APPENDIX 1; APPENDIX 2).

Subjects are required to fast (water permitted) for a minimum of 6 hours prior to the MRS (Cohort A only).

# 6 STUDY ASSESSMENTS AND PROCEDURES (Cohort A and B)

## 6.1 Study Visits

The procedures and assessments for all visits, including Screening, will follow the SOA (APPENDIX 1; APPENDIX 2). Detailed instructions for the procedures and assessments will be provided in the Laboratory Manual.

The study drug should be self-administered at the clinic visit on Day 1 and Day 28 or EOT/ET.

Any impact on the outcomes of this study, including any protocol deviations that result from COVID-19 pandemic or other pandemics or natural disasters, will be discussed in the CSR.

Please note: if allowable measures are included in the protocol, eg, late visits, telephone visits, then it does not need to be a protocol deviation.

## 6.2 Demographics

Age, sex, race, and ethnicity will be recorded for each subject at the Screening Visit. Date of birth will not be recorded.

## 6.3 Medical, Surgical, and Medication Histories

At the Screening Visit, subjects will be interviewed regarding their medical and surgical history and will be recorded in the eCRF. These histories will include past/resolved conditions and active conditions. In addition, the date of last menstrual period will be recorded for female subjects. A medication history will be obtained as part of the medical history. Subjects will be interviewed to provide a list of all prescription medications, over-the-counter medications, and/or dietary supplements (eg, vitamins, herbs, and other dietary supplements) used in the last 4 weeks before the Screening Visit.

## 6.4 Body Measurements

Body measurements to be collected include height and weight, reported in units of kilograms and centimeters, respectively. Height will be measured with the subject’s shoes off. Weight will be measured with the subject’s shoes off and after the bladder has been emptied. Once the subject’s height and weight are entered into the eCRF, the system will compute the BMI.

## 6.5 Laboratory Sample Collections

Blood and urine samples will be collected at the visits indicated in the SOA (APPENDIX 1; APPENDIX 2) for the following assessments:

- Blood collection for local laboratory assessments of safety and tolerability
- Blood and plasma collections for central laboratory assessment of biomarker and other efficacy tests (Cohort A Only)
- Urine collection for the local laboratory assessments of safety and screening

The complete list of analytes to be tested are provided in APPENDIX 3 (Cohort A) and APPENDIX 4 (Cohort B). Detailed instructions for the preparation, handling, and storage of biological samples are provided in the Laboratory Manual.

## 6.6 Safety and Tolerability Assessments

The onset and increase of severity of AEs will be assessed on an ongoing basis. In addition, the safety and tolerability will be monitored by physical examination findings, including body weight; vital signs; and clinical laboratory assessments (chemistry, hematology, and urinalysis).

### 6.6.1 Physical examination

A full physical examination, including a review of all body systems excluding genital examinations, will be performed during Screening to ensure eligibility. Thereafter, a targeted symptom-driven examination can occur based on the occurrence of symptoms (eg, lungs, heart, or based on occurrence of symptoms or AEs).

Physical examination findings will be documented in the subject’s source documents. Any physical examination finding that represents worsening from a baseline condition or clinically significant, as considered by the PI, will be recorded as an AE.

### 6.6.2 Vital signs

Vital signs (sitting systolic and diastolic blood pressure, heart rate, respiratory rate, body temperature, and resting O_2_ saturation) will be measured at each visit except during a telephone visit(s) and recorded in the source documents and eCRF.

The subject should be seated and rested comfortably for at least 5 minutes before obtaining vital signs. Blood pressure may be repeated once to confirm measurement.

Vital signs must be taken before any blood samples are collected at time points where blood is also collected. If possible, vital signs should also be taken before the physical examination is performed, at time points where physical examination is also performed.

### 6.6.3 Echocardiogram

An echocardiogram will be performed at Screening as a criteria for entry into the study. It should rule out significant tricuspid regurgitation, evidence of pulmonary hypertension, or any significant decrease in left ventricular ejection fraction or left ventricle wall abnormality consistent with congestive heart failure.

Note: An echocardiogram is not required to be performed at Screening if documentation of a normal or not clinically significant echocardiogram was performed within 3 months of Screening and after at least 3 months of acute COVID-19 infection. This test is optional depending on the Serum NT-pro BNP test results.

### 6.6.4 Safety laboratory tests

#### 6.6.4.1 Clinical laboratory assessments

Blood and urine samples will be collected for safety and tolerability assessments and will be analyzed at the local laboratory. Procedures for collecting, processing, and handling these samples will be provided in the Laboratory Manual.

The routine clinical laboratory assessments for safety and tolerability include blood sampling for chemistry, hematology, and urine at visits specified in the SOA (APPENDIX 1; APPENDIX 2). The complete list of analytes is provided in APPENDIX 3 (Cohort A) and APPENDIX 4 (Cohort B).

The PI must review the laboratory report, document this review, and record any clinically relevant changes occurring during the study in the AE section of the eCRF. The laboratory reports must be filed with the source documents. Clinically significant abnormal laboratory findings are those that are not associated with the underlying disease, unless judged by the PI to be more severe than expected for the subject’s condition.

If such values do not return to normal, stabilize, or are no longer clinically significant within a period judged reasonable by the PI, the etiology should be identified and the Sponsor should be notified.

A single repeat (retest) per analyte or assessment is allowed during the Screening Period to determine eligibility based on the exclusion criteria and per the PI’s discretion (eg, to recheck laboratory values considered to be out of the typical range for an individual).

All protocol-required laboratory assessments, as defined in APPENDIX 3 (Cohort A) and

APPENDIX 4 (Cohort B), must be conducted in accordance with the Laboratory Manual and the SOA (APPENDIX 1; APPENDIX 2).

#### 6.6.4.2 Urine screen for drugs of abuse

Urine will be collected at Screening and Baseline for drugs of abuse listed in APPENDIX 3 (Cohort A) and APPENDIX 4 (Cohort B).

#### 6.6.4.3 Alcohol breath test

An alcohol breath test will be performed at Screening and Baseline, as specified in the SOA (APPENDIX 1) Cohort A and APPENDIX 2 (Cohort B).

#### 6.6.4.4 Pregnancy tests

Blood will be collected at Screening for a serum pregnancy test to be performed by the local clinical laboratory. Urine will be collected at Baseline and the EOT/ET Visit (Visit 4) for a urine pregnancy test to be performed by the local clinical laboratory.

## 6.7 Collection and Reporting of Adverse Events

AEs including SAEs will be monitored by nonleading questions posed to subjects at each study visit. AEs will be graded by seriousness, severity, and relationship to the study drug for the duration of the study. The PI or delegate will routinely review laboratory results for any clinically significant abnormal results that may represent potential AEs and document any findings in the source documents.

In the event that any subject experience an AE that is considered by the PI to be possibly, probably, or definitely related to study drug and that is severe or medically significant, that subject should be discontinued from further study drug administration and monitored for resolution of the event. Study drug should be discontinued regardless of other factors such as threat to life or admission to hospital.

In the event that 3 subjects experience the same AE meeting the criteria described above or when 2 subjects experience the same AE that is life-threatening or requires immediate intervention to prevent death and that is attributed to study drug, enrollment in the study should be temporarily halted to allow further evaluation.

### 6.7.1 Adverse event and treatment-emergent adverse event

As defined by the International Council for Harmonisation (ICH), an AE is any untoward medical occurrence in a patient or clinical investigation subject who consumes a study drug, which does not necessarily have a causal relationship with the study drug. An AE can, therefore, be any unfavorable and unintended sign (including an abnormal laboratory finding), symptom, or disease temporally associated with the use of a medicinal (investigational or test) product, whether or not considered related to the study drug.

A TEAE is defined as any AE that begins or worsens in severity after the first dose of study drug is taken.

If an abnormal laboratory result or vital sign is recorded and deemed a clinically significant change compared to the baseline value(s), the PI will also report these findings as an AE.

AEs leading to discontinuation or reduction in intake of study drug will be reported as AEs of interest within the standard AE reporting timeframe.

### 6.7.2 Serious adverse event

An AE is considered “serious” if, in the view of either the PI or the Sponsor, it results in any of the following outcomes:

- Death
- A life-threatening condition
- Inpatient hospitalization or prolongation of existing hospitalization
- A persistent or significant incapacity or substantial disruption of the ability to conduct normal life functions
- A congenital anomaly/birth defect

Important medical events that may not result in death, be life-threatening, or require hospitalization may be considered serious when, based upon appropriate medical judgement, they may jeopardize the subject and may require medical or surgical intervention to prevent one of the outcomes listed in this definition.

### 6.7.3 Classification of adverse events

#### 6.7.3.1 Severity

The severity of AEs should be graded using the CTCAE version 5.0 (CTCAE).

For AE terms that are not listed in the CTCAE, the following grading system should be used:

- CTCAE Grade 1: Mild; asymptomatic or mild symptoms; clinical or diagnostic observations only; intervention not indicated;
- CTCAE Grade 2: Moderate; minimal, local, or non-invasive intervention indicated; limiting age-appropriate instrumental activities of daily living;
- CTCAE Grade 3: Severe or medically significant but not immediately life-threatening; hospitalization or prolongation of hospitalization indicated; disabling; limiting self-care activities of daily living;
- CTCAE Grade 4: Life-threatening consequences; urgent intervention indicated; and
- CTCAE Grade 5: Death related to AE.

#### 6.7.3.2 Relationship

The relationship of an AE to the administration of the study drug is to be assessed according to the following definitions:

No (unrelated, not related, or unlikely to be related)–- The time course between the administration of study drug and the occurrence or worsening of the AE rules out a causal relationship and another cause (concomitant drugs, therapies, complications, etc) is suspected.

Yes (possibly, probably, or definitely related)–- The time course between the administration of study drug and the occurrence or worsening of the AE is consistent with a causal relationship and no other cause (concomitant drugs, therapies, complications, etc) can be identified. The definition implies a reasonable possibility of a causal relationship between the event and the study drug. This means that there are facts (evidence) or arguments to suggest a causal relationship.

The following factors should also be considered:

- The temporal sequence from study drug administration-

The event should occur after the study drug is given. The length of time from the study drug exposure to the event should be evaluated in the clinical context of the event.

- Underlying, concomitant, intercurrent diseases-

Each report should be evaluated in the context of the natural history and course of the disease being treated and any other disease the subject may have.

- Concomitant drug-

The other drugs the subject is taking or the treatment the subject receives should be examined to determine whether any of them might be recognized to cause the event in question.

- Known response pattern for this class of study drug-

Clinical and/or preclinical data may indicate whether a particular response is likely to be a class effect.

- Exposure to physical and/or mental stresses-

The exposure to stress might induce adverse changes in the recipient and provide a logical and better explanation for the event.

- The pharmacology and PK of the study drug-

The known pharmacologic properties (absorption, distribution, metabolism, and excretion) of the study drug should be considered.

#### 6.7.3.3 Expectedness

Expectedness will be determined by the Sponsor or designee. An AE will be considered unexpected if the event is not listed in the Reference Safety Information section of the current version of the IB.

### 6.7.4 Reporting of adverse event and serious adverse event

#### 6.7.4.1 Adverse event

AEs, which include clinical laboratory test results, will be monitored and documented from the time of informed consent until following the last administration of study drug through the Follow-up Period (Day 35). Subjects should be instructed to report any AE that they experience to the PI, whether or not they think the event is due to study drug. The PIs should make an assessment for each AE at each visit and record the event on the appropriate AE eCRF. Each AE should be followed until it resolves or until the end of the Follow-up Period.

Wherever possible, a specific disease or syndrome rather than individual-associated signs and symptoms should be identified by the PI and recorded on the eCRF. However, if an observed or reported sign or symptom is not considered a component of a specific disease or syndrome by the PI, it should be recorded as a separate AE on the eCRF. Additionally, the condition that led to a medical or surgical procedure (eg, surgery, endoscopy, tooth extraction, or transfusion) should be recorded as an AE, not the procedure itself.

Any medical condition already present at the Screening Visit should be recorded as medical history and not be reported as an AE unless the medical condition or signs or symptoms present at Baseline changes in severity, frequency, or seriousness at any time during the study. In this case, it should be reported as an AE.

Clinically significant abnormal laboratory or other examination (eg, echocardiogram) findings that are detected during the study or are present at the Screening Visit and significantly worsen during the study should be reported as AEs, as described below. The PI will exercise their medical and scientific judgement in deciding whether an abnormal laboratory finding or other abnormal assessment is clinically significant. Clinically significant abnormal laboratory values occurring during the clinical study will be followed until follow-up tests return to normal, stabilize, or are no longer clinically significant. Abnormal test results that are determined to be an error should not be reported as an AE. Laboratory abnormalities or other abnormal clinical findings (eg, echocardiogram abnormalities) should be reported as an AE if any of the following are applicable:

- If an intervention is required as a result of the abnormality;
- If an action taken with the study drug is required as a result of the abnormality; or
- Based on the clinical judgement of the PI.

#### 6.7.4.2 Serious adverse event

Collection of SAEs will only be up to 7 days following the last dose, however, follow-up on ongoing SAEs will continue until resolution or 4 weeks following the administration of the last dose of the study drug. After the 7 days reporting window, any SAE that the PI considers related to the study drug must be reported to Medpace Clinical Safety and the Sponsor/designee.

To report the SAE, complete the SAE form electronically in the eCRF. When the form is completed, Medpace Safety personnel will be notified electronically and will retrieve the form. If the event meets serious criteria and it is not possible to access the eCRF, send an email to Medpace Safety at **medpace-safetynotification@medpace.com** or call the Medpace SAE hotline (phone number listed below) and fax/email the completed paper SAE form to Medpace (contact information listed below) within 24 hours of awareness. When the eCRF becomes available, the SAE information must be entered within 24 hours of the system becoming available.

The PI must continue to follow up the subject until the SAE has subsided or until the condition becomes chronic in nature or stabilizes (in the case of persistent impairment) or the subject dies.

Within 24 hours of receipt of follow-up information, the PI must update the SAE form electronically in the eCRF and submit any supporting documentation (eg, subject discharge summary or autopsy reports) to Medpace Clinical Safety via fax or email. If it is not possible to access the eCRF, refer to the procedures outlined above for initial reporting of SAEs.

Safety contact information Medpace Clinical Safety

Medpace SAE hotline – USA:

Telephone: +1-800-730-5779, dial “3” or +1-513-579-9911, dial “3”

Fax: +1-866-336-5320 or +1-1-513-570-5196

Email: medpace-safetynotification@medpace.com

Medpace SAE hotline – Europe:

Telephone: +49 89 89 55 718 44

Fax: +49 89 89 55 718 104

Email: medpace-safetynotification@medpace.com.

### 6.7.5 Pregnancy reporting

If a subject becomes pregnant during the study or within the Follow-up Period defined in the protocol, the PI is to stop dosing with study drug immediately and the subject should be withdrawn from the study. ET procedures should be implemented at that time.

A pregnancy is not considered to be an AE or SAE; however, it must be reported to Medpace Clinical Safety within 24 hours of knowledge of the event. Medpace Clinical Safety will then provide the PI/study site the Exposure In Utero (EIU) form for completion. The PI/study site must complete the EIU form and fax/email it back to Medpace Clinical Safety.

If the female partner of a male subject becomes pregnant while the subject is receiving study drug or within the Follow-up Period defined in the protocol, the PI should notify Medpace Clinical Safety as described above.

The pregnancy should be followed until the outcome of the pregnancy, whenever possible. Once the outcome of the pregnancy is known, the follow-up EIU form should be completed and faxed/emailed to Medpace Clinical Safety. If the outcome of the pregnancy meets the criteria for immediate classification as an SAE (ie, postpartum complication, spontaneous abortion, stillbirth, neonatal death, or congenital anomaly), the PI should follow the procedures for reporting an SAE.

### 6.7.6 Medical events of interest

No medical events of interest have been identified.

## 6.8 Efficacy Assessments

### 6.8.1 Phosphorus Magnetic Resonance Spectroscopy and Proton Magnetic Resonance

##### Spectroscopy (Cohort A Only)

31P-MRS and 1H-MRS will be performed prior to and following moderate exercise at Screening (Visit 1) and EOT/ET (Visit 4). The 31P-MRS and 1H-MRS acquisition protocol and guide is provided in the Study Reference Manual.

Note: A plasma analysis by 31P-MRS and 1H-MRS is not required to be performed at Screening if documentation of a plasma analysis by 31P-MRS and 1H-MRS was performed within 3 months of Screening and after 3 months of acute COVID-19 infection. If a historical 31P-MRS and 1H-MRS has been used to determine eligibility, then 31P-MRS and 1H-MRS should be repeated at Baseline to confirm eligibility and establish baseline.

31P-MRS is used to estimate the concentration of high-energy phosphate compounds; thus, the bioenergetic state of a tissue can be characterized in vivo. Accordingly, 31P-MRS can offer an alternative to histopathology for assessing the progression of the disease. The classic dynamic MRS experiment is to monitor the bioenergetic response of 31P metabolites to a sustained muscle contraction or exercise. To maintain the intracellular ATP concentration during this increased energy demand, PCr is depleted, inorganic phosphate (Pi) accumulates, and intracellular pH may also be modulated as a result of anaerobic glycolysis. Although the intracellular concentration of free (unbound) ADP is too low to be observed directly with 31P-MRS, it can be calculated indirectly, assuming equilibrium at creatine kinase. Upon cessation of exercise, each metabolite recovers to baseline levels. With relatively rapid (<8 seconds) time resolution and certain assumptions, a range of bioenergetic fluxes can be determined from the metabolic changes. In particular, a functional estimate of mitochondrial oxidative capacity can be calculated from the kinetics of PCr recovery rate. A recent study demonstrated the correlation between PCr, muscle strength, and walking speed in elderly subjects (Zane 2017).

MRS studies have demonstrated that muscle oxidative capacity is increased by endurance training, consistent with mitochondrial biogenesis and enhanced aerobic performance, and that the metabolic perturbations associated with exercise are attenuated. Bioenergetic deficits may predict clinical severity and are accompanied by decreased creatine content, as determined by 1H-MRS. Lipotoxicity influences mitochondrial function, and the amount of intramyocellular lipids increases in proportion to decreasing mitochondrial function. In a similar way, acetylcarnitine provides insights into fatty acid oxidation chains as a buffer for the pyruvate dehydrogenase flux. In addition, mitochondrial function is hampered at low intramuscular pH, and while the pH buffering capacity of skeletal muscle is very complex with several mechanisms involved, carnosine concentration (one of the metabolites responsible for pH buffering) has been shown to correlate with mitochondrial function. All of these metabolites can be non-invasively measured using 1H-MRS.

For additional information, refer to the Laboratory Manual.

### 6.8.2 Chalder Fatigue Questionnaire (CFQ-11 PRO for fatigue) (Cohort A and B)

This self-administered questionnaire will be provided to the subject at Screening (Visit 1), Visit 2, Visit 3, and EOT/ET (Visit 4). At Visit 3 (the telephone visit), it will be read to the subject or provided by secure electronic fashion. At all visits other than Screening, it will be performed before and after a 6MWT evaluation (Cohort A) and only before 6MWT evaluation (Cohort B).

### 6.8.3 6-Minute Walk Test (Cohort A and B)

The 6MWT assess the distance walked over 6 minutes as a submaximal test of aerobic capacity and endurance. The subject may take as many standing rests as they like, but the administrator should keep going and record the number of rests. If a subject requires assistance, only the minimum amount of assistance required for a subject to complete the task should be provided. The level of assistance if documented, however, should reflect the greatest amount of assistance provided. The administrator should walk behind the subject instead of beside or in front of the subject. This test should be performed at (or within 7 days of) Day 1 and EOT/ET (Visit 4). The subject may have clinic visit assessments performed over 2 days. The subject will be provided an overnight stay at a nearby hotel.

Detailed description of the 6MWT is provided in Study Reference Manual.

### 6.8.4 Serum Lactate Test (Cohort A Only)

A serum lactate level that measures the amount of lactic acid in the body will be obtained at Day 1 and EOT/ET (Visit 4).

Also in association with moderate exercise, capillary blood lactate level is to be measured by earlobe puncture just before starting, immediately after and 5, 10, 20, and 30 minutes after the 6MWT. Detailed instructions will be provided in the Laboratory Manual.

## 6.9 Pharmacokinetic Assessments

Not applicable.

## 6.10 Exploratory Assessments (Cohort A Only)

Blood samples for exploratory biomarker assessment will be obtained according to schedule at Day 1 and EOT/ET (Visit 4) and sent to the central laboratory for analysis. The analytes are indicated in

APPENDIX 3.

For ALL subjects in the study, predose blood samples will be collected at Day 1 and at EOT/ET (Visit 4). Blood samples for AA concentration are to be collected no more than 30 minutes before administration of study drug and after a fast (minimum duration) of 10 hours although water is permitted. The actual date and time (24-hour clock time) of each sample will be recorded in the eCRF. Plasma concentration of AAs will be analyzed by validated liquid chromatography with tandem mass spectrometry method. Details of the blood collection, processing, and handling samples and the analytical methods will be provided in the Laboratory Manual.

# 7 STATISTICAL CONSIDERATIONS

## 7.1 Sample Size Estimation (Cohort A and B)

**Cohort A**

The sample size calculation is based on the primary efficacy endpoint, the mean change from baseline at Week 4 in the PCr recovery rate. Based on literature review, the standard deviation (SD) of both the AXA1125 and the placebo group is assumed to be 10 seconds. To detect a clinically meaningful difference of 10-second improvement, approximately 32 subjects will provide 80% power at a 2-sided, 5% significance level. Assuming 20% dropout rate, approximately 40 subjects (20 subjects per arm) will be enrolled in this study.

This sample size will also allow for a general assessment of safety and tolerability of AXA1125 in this population. **Cohort B** Approximately 10 subjects (5 subjects per arm), who were considered screen-failures for Cohort A due to their PCr recovery rate constant (< 50seconds) provided their CFQ-11 is ≥ 8 and they meet all the eligibility criteria will be enrolled in this Cohort.

## 7.2 Populations for Analysis (Cohort A and B)

### 7.2.1 Intent-to-treat analysis set

The intent-to-treat (ITT) population will consist of all subjects randomized who received at least 1 dose of the study drug. Subjects will be assessed according to their randomized treatment regardless of the treatment they received. The primary population for efficacy analyses is the ITT analysis set.

### 7.2.2 Safety analysis set

The Safety Analysis Set will include all subjects who receive at least 1 dose of study drug. Subjects in the Safety Analysis Set will be analyzed by the treatment received on Day 1. The primary population for safety analyses is the Safety Analysis Set.

### 7.2.3 Per-protocol analysis set

The per-protocol (PP) analysis set will include all subjects in the ITT population without any major protocol deviations. All analyses performed in the PP analyses set will be considered as sensitivity analyses.

### 7.2.4 Pharmacokinetic analysis set (Cohort A)

The PK Analysis Set will comprise all subjects in the Safety Analysis Set who have evaluable PK concentration data for at least 1 AA.

## 7.3 Study Endpoints (Cohort A and B)

The study endpoints are presented in Section 2.3.

## 7.4 Statistical Methods (Cohort A and B)

### 7.4.1 General approach

Descriptive statistics for continuous variables will include the number of subjects with data to be summarized, mean, SD, median, and range (minimum, maximum).

All categorical/qualitative data will be presented using frequency counts and percentages.

The statistical methods for comparison of each endpoint and complete missing imputation methods will be detailed in the Statistical Analysis Plan (SAP), which will be developed and completed prior to database lock.

There will be no multiplicity adjustment and all analyses will be performed at the 2-sided 0.05 level of significance.

### 7.4.2 Randomization and blinding

At Visit 2, eligible subjects will be randomized through an IRT system into 1 of 2 groups (33.9 g BID AXA1125 or placebo BID) at a treatment allocation ratio of 1:1.

This is a double-blind study design. The subject, investigators, study site personnel involved in direct care of the subject will be blinded to treatment assignment. Personnel involved in the laboratory and image analysis will also remain blinded to the treatment assignment. The Sponsor study team members will remain blinded to the treatment assignments during the study. A Sponsor biostatistician will be unblinded to perform the interim analysis and data monitoring. See Section 5.1.6 for details on blinding.

#### 7.4.2.1 Breaking the blind

The PI is responsible for the medical care of subjects during the study. In an emergency, when knowledge of the subject’s treatment assignment is essential for the clinical management or welfare of the subject, the PI can unblind the treatment information for the subject through the IRT system. The unblinding should only occur for the subject in question if it is critical for treatment decision making by the PI for the well-being of the subject.

Prior to unblinding the subject’s treatment assignment, the PI should assess the relationship of an AE to the study drug (yes or no). In the vast majority of cases, AEs may be properly managed without the need for unblinding. If unblinding is warranted, the PI must then follow the appropriate procedures to unblind an individual subject’s treatment assignment. Generally, blinding should only be broken for events that are considered to be serious, unexpected, and causally related to the study drug, or as requested by the regulatory authority.

If the study blind is broken, the PI must detail the date and reason for unblinding in the subject’s records. The PI must also notify the Sponsor and if applicable, the regulatory authority, that the study blind has been broken. If the blind is broken due to an AE, the AE form must be completed and reported to the Sponsor.

### 7.4.3 Handling of dropouts/missing data

For binary endpoints, both worst-case and observed-case methods will be applied as the missing imputation method. Worst-case method is defined as subjects with missing data due to any reason will be considered as not meeting the endpoint. Observed-case imputation method is defined as subjects with missing data will not be included in the analysis. For continuous endpoints, both single and multiple missing imputations will be applied.

To address each of potential intercurrent events, the strategies will be detailed in the SAP, if appropriate.

### 7.4.4 Subject disposition

The number of subjects in each study population will be provided. The number and percentage of subjects who prematurely discontinue study drug along with disposition (completed study or early withdrawal, with breakdown for reasons for withdrawal) will be summarized. The denominator will be the number of subjects in the ITT population.

### 7.4.5 Demographics and baseline characteristics

Subject demographics and baseline characteristics, including age, sex, race, ethnicity, weight, height, and BMI will be summarized using descriptive statistics. Continuous data will be summarized with mean, SD, median, minimum, and maximum values. Categorical data will be summarized with frequency counts and percentages.

“Baseline” is defined as the most recent assessment prior to randomization.

Medical history and concomitant medications will be summarized.

### 7.4.6 Safety analyses

The safety data will be presented as individual listings and summary tables, including frequency tables for AEs and frequency and shift tables for laboratory evaluations, vital signs, and physical examinations. Shift tables will be generated for key safety parameters and graphical presentations will be utilized as needed.

AEs will be coded using the Medical Dictionary for Regulatory Activities (MedDRA). Summary tables for AEs will include all TEAEs, related TEAEs, SAEs, TEAEs by severity, and TEAEs leading to study drug discontinuation. Summaries will be presented by MedDRA system organ class and preferred term using frequency counts and percentages. All safety analyses will be performed in the Safety Analysis Set.

### 7.4.7 Efficacy analyses (Cohort A and B)

#### 7.4.7.1 Primary and key secondary efficacy endpoint analyses

The categorical endpoints will be analyzed using the Chi-square test. Subgroup analyses, such as by gender, may be performed.

Continuous endpoints will be analyzed using the analysis of covariance models adjusted for the baseline value. Mixed model repeated measures models will be applied as sensitivity analyses.

### 7.4.8 Interim analyses and data monitoring (Cohort A only)

An interim analysis with potential sample size re-estimation will be performed when at least 10 subjects complete their Week 4 visit. Periodical administrative analyses may be performed to closely monitor the study.

### 7.4.9 Pharmacokinetic analyses (Cohort A only)

For all subjects in the study (Cohort A only), concentration data listings and descriptive statistics (arithmetic and geometric means, SD, coefficient of variation, median, minimum, and maximum) for fasted AAs (postdose plasma concentrations of AXA1125 dosed and non-dosed AAs) will be provided at all planned predose sampling time points. Concentrations determined for samples from ET visits, if any, and from the final clinic visit will be included in listings but not in descriptive statistics. For sampling time points, see the SOA (APPENDIX 1; APPENDIX 2).

In addition, the plasma concentration data from this study may be combined with data from other studies to perform a population PK analysis and exposure-response analyses, which will follow the principles outlined in the United States Food and Drug Administration (FDA) Guidance for Industry 2019 (FDA Guidance 2019) and may be reported separately.

Results from metabolite profiling analysis of leftover predose and postdose plasma PK samples will be reported separately, if applicable.

# 8 SUPPORTING DOCUMENTATION AND OPERATIONAL CONSIDERATIONS (Cohort A and B)

## 8.1 Regulatory, Ethical, and Study Oversight Considerations

This study will be initiated only after all required legal documentation has been reviewed and approved by the respective Institutional Review Board (IRB)/Independent Ethics Committee (IEC) and regulatory authorities according to national and international regulations. The same applies for the implementation of changes introduced by amendments.

This study will be conducted in full conformity with the ICH E6 (R2) Good Clinical Practice (GCP) guideline and applicable local regulations governing clinical research.

The rights of the PI and of the Sponsor with regard to publication of the study results are described in the PI contract. As a rule, no study results should be published before the CSR is finalized.

### 8.1.1 Subject information and informed consent

The informed consent form (ICF) and other forms used to document informed consent and/or permission must be agreed to by the Sponsor and approved by the IRB/IEC or have a favorable opinion from the IEC. A copy of the IRB/IEC document approving the protocol, any protocol amendments, the ICF, and permission forms must be supplied to the Sponsor before starting the study (that is, before performing any study procedure, including screening tests).

After being given an explanation of the study and before participating in any study procedures, each subject must provide voluntary and written informed consent in compliance with ICH/GCP and the regulatory and legal requirements of the participating country.

The signed documents will be placed in the PI’s study files.

#### 8.1.1.1 Written informed consent

Written informed consent will be obtained from the subject before the subject participates in any study-related procedure. To provide consent for study participation, the subject will read, assent to an understanding of, and sign an instrument of informed consent or other locally applicable regulations and authorization form after having had an opportunity to discuss the forms with the PI before signing. The subject will be made aware that the subject may withdraw from the study at any time and will receive a copy of the signed ICF.

### 8.1.2 Ethical committee and regulatory authority review

The protocol, ICF, recruitment materials, and all materials which will be provided to subjects will be submitted to an IRB/IEC for their review and approval/favorable opinion. Approval of the protocol and all consent/permission forms must be obtained before any subject is enrolled. Any amendment to the protocol must be approved by (or a favorable opinion received from) the IRB/IEC before the changes to the study are implemented.

All changes to the ICF must be approved by (or a favorable opinion received from) the IRB/IEC before the revised version is used.

A protocol change intended to eliminate an apparent immediate hazard to subject safety may be implemented immediately provided that the Sponsor and the IRB/IEC are immediately notified.

Where applicable based on local regulations, study documents (eg, protocol and ICF) will also be submitted to and approved by the regulatory authority before the document is utilized for study conduct.

### 8.1.3 Quality assurance and quality control

Quality control procedures will be implemented beginning with the data entry system. Data quality control checks that will be run on the database will be generated. Any missing data or data anomalies will be communicated to the study site(s) for clarification/resolution. Additional details may be found in the study-specific Data Management Plan and in the list of logic checks implemented within the electronic database system.

Following written standard operating procedures, the monitors will verify that the clinical study is conducted and that data are generated, documented (recorded), and reported in compliance with the protocol, including any amendments, GCP, and any applicable regulatory requirements.

The Sponsor or designee will monitor the conduct of study by regular site monitoring visits and in-house data quality review. The frequency of site monitoring will be determined by assessing all characteristics of the study, including its nature, objective, methodology, and the degree of any deviations of the intervention from normal clinical practice.

The PI/institution will permit study-related monitoring, audits, IRB/IEC review, and regulatory inspections. Direct access must be provided to the eCRF and all source documents/data, including progress notes and copies of laboratory and medical test results, which must be available at all times for review by the study monitor, auditor, and regulatory inspector (eg, FDA). The study monitor and auditor may review all eCRFs and ICFs. The accuracy of the data will be verified by direct comparison with source documents.

Quality assurance covers availability for inspections. Records must be available for direct inspection, verification, and copying, as required by applicable laws and regulations and by officials of the regulatory health authorities. The PI will comply with applicable privacy and security laws for use and disclosure of information.

## 8.2 Administrative and Legal Obligations

### 8.2.1 Protocol amendments and study termination

Any amendments to the study protocol will be communicated to the PIs by Medpace or the Sponsor. All protocol amendments will undergo the same review and approval process as the original protocol. A protocol amendment may be implemented after it has been approved by the IRB/IEC unless immediate implementation of the change is necessary for subject safety. In this case, the situation must be documented and reported to the IRB/IEC within 5 working days.

#### 8.2.1.1 Study termination

Intake of study drug may be halted if the Sponsor/Medical Monitor determines there is any unexpected, significant, or unacceptable risk to subjects upon ongoing review of aggregate safety data; the Sponsor will inform the PI within 24 hours after this determination.

This study may be suspended or prematurely terminated by the Sponsor if there is sufficient reasonable cause. Written notification, documenting the reason for study suspension or termination, will be provided by the Sponsor to the PI. If the study is prematurely terminated or suspended, the PI will promptly inform the IRB/IEC and will provide the reason(s) for the termination or suspension. Circumstances that may warrant termination or suspension of the conduct of the study include, but are not limited to the following:

- Determination of unexpected, significant, or unacceptable risk to subjects, as determined by the ongoing review of aggregate safety data by the Sponsor/Medical Monitor;
- Insufficient compliance with protocol requirements by study sites;
- Consistently insufficient, incomplete, and/or unevaluable data submission by the study sites;
- 3 subjects develop the same CTCAE Grade 3 AE determined as related to study drug;
- 2 subjects develop any CTCAE Grade 4 AE determined as related to study drug; OR • 1 subject develops a CTCAE Grade 5 AE regardless of its relationship to the study drug.

In the event of a potentially related Grade 5 event or Suspected Unexpected Serious Adverse Reaction (SUSAR), available information will be reviewed which includes the potential of unblinding the subject, and will communicate this information to the unblinded Chief Medical Officer for determination on any potential modification of study procedures to insure subjects’ safety, which may or may not include termination of the study. Review of the potentially related Grade 5 events or SUSARs will occur within 48 hours of the Sponsor making the determination that the event may be related to study drug.

Reporting requirements are outlined in the study Safety Monitoring Plan, including the timelines for completion by study sites and submission to the appropriate regulatory agencies.

If the study is suspended temporarily, the study may resume once concerns about safety and tolerability, protocol compliance, and/or data quality are addressed and satisfy the Sponsor, regulatory authorities, and/or IRB/IEC (including approval of a substantial amendment, if required).

### 8.2.2 Study documentation and archive

The study site will maintain appropriate medical and research records for this study, in compliance with ICH E6 (R2) and regulatory and institutional requirements for the protection of confidentiality of subjects. The PI must make study data accessible to the Sponsor, to other authorized representatives of the Sponsor, and to the appropriate regulatory authority inspectors.

Source data are all information, original records of clinical findings, observations, or other activities in a clinical study necessary for the reconstruction and evaluation of the study.

Examples of these original documents and data records include, but are not limited to, hospital records, clinical and office charts, laboratory notes, memoranda, subjects’ questionnaires and diaries, pharmacy dispensing records, copies or transcriptions certified after verification as being accurate and complete, X-rays, and subject files and records kept at the pharmacy and at the laboratories involved in the clinical study.

#### 8.2.2.1 Electronic case report forms

Data collection is the responsibility of the clinical study staff at the study site under the supervision of the study site PI. The PI is responsible for ensuring the accuracy, completeness, legibility, and timeliness of the source data and the data reported.

Clinical data will be entered directly into the eCRFs from the source documents. Data reported in the eCRF derived from source documents should be consistent with the source documents, or the discrepancies should be explained and captured in a progress note and maintained in the subject’s official electronic study record.

Clinical data (including AEs, concomitant medications, and expected adverse reactions data) and clinical laboratory data will be entered into eCRF that is compliant with the Part 11 (United States) and European Union Annex 11 requirements for validation of computer systems. The data system includes password protection and internal quality checks, such as automatic range checks, to identify data that appear inconsistent, incomplete, or inaccurate.

#### 8.2.2.2 Record retention

As required by law, study documents should be retained for a minimum of 2 years after the last approval of a marketing application in an ICH region and until there are no pending or contemplated marketing applications in an ICH region or until at least 2 years have elapsed since the formal discontinuation of clinical development of the study drug. Since the study drug being utilized in this protocol may or may not be a part of a marketing application, study documents shall be retained as described in the Clinical Study Agreement (CSA). These documents should be retained for a longer period, however, if required by local regulations. No records will be destroyed without the written consent of the Sponsor, consistent with the terms agreed in the CSA. It is the responsibility of the Sponsor to inform the PI when these documents no longer need to be retained.

### 8.2.3 Study monitoring

Study site monitoring will be conducted to ensure that the rights and well-being of human subjects are protected; that the reported study data are accurate, complete, and verifiable; and that the conduct of the study complies with the currently approved protocol/amendment(s), with GCPs, and with applicable regulatory requirements.

Details of study site monitoring are documented in the Monitoring Plan. The Monitoring Plan describes in detail how monitoring will be conducted, who will conduct the monitoring, at what frequency monitoring will be done, at what level of detail monitoring will be performed, and the distribution of monitoring reports.

Independent audits may be conducted to ensure monitoring practices are performed consistently across all participating study sites and that monitors are following the Monitoring Plan.

### 8.2.4 Materials control, accountability, and disposition

The study site will retain all used and unused study drug for the monitor to perform accountability. At the termination of the study or at the request of the Sponsor, the PI or designee will destroy or return unused study drug and all partially dispensed or empty sachets. Such activities pertaining to the destruction or return of unused study drug will be documented per Sponsor request.

### 8.2.5 Confidentiality

The PI will comply with all applicable federal, state, and local laws and regulations relating to the privacy of subjects’ health information. The PI shall ensure that subjects authorize the use and disclosure of protected health information in accordance with the local privacy regulations and in a form acceptable to the Sponsor. Prior to any testing of this protocol, including screening tests and assessments, candidates must also provide all authorizations required by local law. The study protocol, documentation, data, and all other information generated will be held in strict confidence. With the exception of emergency or specialist care, no information concerning the study, or the data, will be released to any unauthorized third party without prior written approval of the Sponsor.

The study monitor, other authorized representatives of the Sponsor, and/or representatives of the IRB/IEC or regulatory authorities may inspect all documents and records required to be maintained by the PI, including but not limited to medical records (office, clinic, or hospital) and pharmacy records for the subjects in this study.

Each subject’s contact information will be securely stored at each study site for internal use during the study. At the end of the study, all records will continue to be kept in a secure location until records no longer need to be retained per the terms in the CSA and applicable regulatory requirements.

During the study, each subject’s race, ethnicity, and age will be collected (unless the collection is not permitted by applicable law or not approved by the governing ethics committee). Since it is not known whether the effects of the study drug are influenced by race or ethnicity, this information will be of value in the analysis of safety. The age information is necessary to confirm study eligibility, to facilitate clinical assessments, to employ age-dependent laboratory reference intervals, and to conduct the planned statistical analysis.

Subjects will not be identified by name in the eCRF or in any study report, and these reports will be used for research purposes only. The Sponsor, its partners and designees, ethics committees, and various government health agencies may inspect the records of this study. Every effort will be made to keep the subject’s personal medical data confidential.

### 8.2.6 Disclosure of data

Details and terms on publication and data sharing will be specified in the CSA between the Sponsor and study site.

## 8.3 Protocol Deviations

A protocol deviation is noncompliance with the requirements of the clinical study protocol, GCP, Study Reference Manual, or other study agreements. The noncompliance may be either on the part of the subject, the PI, or the study site staff. As a result of deviations, corrective actions may be developed and implemented promptly.

These practices are consistent with the following sections in the ICH E6 (R2) guideline:

- Section 4.5 Compliance with Protocol, Subsections 4.5.1, 4.5.2, 4.5.3, and 4.5.4; • Section 5.1 Quality Assurance and Quality Control, Subsection 5.1.1; and
- Section 5.20 Noncompliance, Subsections 5.20.1 and 5.20.2.

Protocol deviations relating to individual subjects must be addressed in the subject’s source documents and on the appropriate eCRF and reported to the Sponsor. Deviations that are not subject specific (eg, unauthorized use of a study drug outside of the study) will be reported to the Sponsor and a copy of the report will be filed in the study-specific trial master file. Protocol deviations must be reported to the study site’s IRB/IEC per their guidelines. The PI and study site staff members are responsible for knowing and adhering to their IRB/IEC requirements.

## 8.4 Research Use of Stored Human Samples and Data

Research use of stored human samples and data will comply with applicable local laws, regulations, and guidelines.

Samples and data collected under this protocol will be used to study the objectives described in Section 2.1. No genetic testing of human samples will be performed.

Access to stored samples and data will be limited. Samples and data will be stored using codes assigned through the study. Data will be kept in password-protected computers, access- and role-restricted databases, and sample tracking systems. Only the study site’s delegated study personnel and Sponsor’s study team members will have access to the stored samples and data.

Samples and data collected in this study may be used for the Sponsor’s future research and development purposes. The Sponsor’s researchers and its designees may also be provided with a code-link that will allow linking the biological samples with the phenotypic data from each subject, maintaining the de-identification of each subject’s identity. When the study is completed, access to study data and/or samples will be obtained through the Sponsor.

Results from analysis of the data and samples will be stored by the Sponsor. After the study is completed, the de-identified, archived data and samples will be stored by the Sponsor under the supervision of the Chief Medical Officer or designee. Permission to store data and samples by the Sponsor will be included in the informed consent. De-identified data may be published or shared with third parties.

## 8.5 End of Study

For the purposes of this protocol, “end of study” is defined as the completion of the last subject visit in both Cohort A and Cohort B.

# 9 LIST OF REFERENCES

###### Altay 2021

Altay O, Arif M, Li X, et al. Combined metabolic activators accelerates recovery in mild-to-moderate COVID-19. medRxiv. Posted 20 February 2021 (preprint); doi.org/10.1101/2020.10.02.20202614.

###### Blomstrand 1997

Blomstrand E, Hassmén P, Ek S, et al. Influence of ingesting a solution of branched-chain amino acids on perceived exertion during exercise. *Acta Physiol Scand.* 1997;159(1):41-49. **Bröer 2008**

Bröer S. Amino acid transport across mammalian intestinal and renal epithelia. *Physiol Rev*. 2008;88(1):249-286.

###### Bröer and Fairweather 2018

Bröer S, Fairweather SJ. Amino acid transport across the mammalian intestine. *Compr Physiol*. 2018;9(1):343-373. **Caussy 2020**

Caussy C, Brissot J, Singh S, et al. Prospective, same day, direct comparison of controlled attenuation parameter with the M vs the XL probe in patients with nonalcoholic fatty liver disease, using magnetic resonance imaging-proton density fat fraction as the standard. *Clin Gastroenterol Hepatol*. 2020;18(8):1842-1850.e6.

###### Cengiz 2020

Cengiz M, Uysal BB, Ikitimur H, et al. Effect of oral l-Glutamine supplementation on COVID-19 treatment. *Clin Nutr Exp*. 2020;33:24-31.

###### Coqueiro 2019

Coqueiro AY, Rogero MM, Tirapegui J. Glutamine as an anti-fatigue amino acid in sports nutrition. *Nutrients.* 2019;11(4):863.

###### Crook 2021

Crook H, Raza S, Nowell J, et al. Long COVID-mechanisms, risk factors, and management. *BMJ*. 2021;374:n1648.

###### CTCAE

Common Terminology Criteria for Adverse Events (CTCAE). 2017. Version 5.0.

https://ctep.cancer.gov/protocoldevelopment/electronic_applications/docs/ctcae_v5_quick_reference_ 5x7.pdf. Accessed 10 August 2021.

###### FAIR Health 2021

FAIR Health. A detailed study of patients with long-haul COVID: an analysis of private healthcare claims. Jun 2021[. https://www.fairhealth.org/publications/whitepapers](https://www.fairhealth.org/publications/whitepapers); Accessed August 2021. **FDA Guidance 2019**

Food and Drug Administration. Guidance for Industry: Population Pharmacokinetics. July 2019. https://www.fda.gov/media/128793/download. Accessed September 2021.

###### Fluge 2016

Fluge Ø, Mella O, Bruland O, et al. Metabolic profiling indicates impaired pyruvate dehydrogenase function in myalgic encephalopathy/chronic fatigue syndrome. *JCI Insight*. 2016;1(21):e89376.

###### Fluge 2021

Fluge Ø, Tronstad KJ, Mella O. Pathomechanisms and possible interventions in myalgic encephalomyelitis/chronic fatigue syndrome (ME/CFS). *J Clin Invest*. 2021;131(14):e150377.

###### García-Campos 2020

García-Campos P, Báez-Matus X, Jara-Gutiérrez C, et al. N-acetylcysteine reduces skeletal muscles oxidative stress and improves grip strength in dysferlin-deficient Bla/J mice. *Int J Mol Sci*. 2020;21(12):4293.

###### Gee and Deniel 2016

Gee T, Deniel S. Branched-chain amino acid supplementation attenuates a decrease in power-producing ability following acute strength training. *J Sports Med Phys Fitness.* 2016;56(12):1511-1517.

###### Germain 2017

Germain A, Ruppert D, Levine SM, et al. Metabolic profiling of a myalgic encephalomyelitis/chronic fatigue syndrome discovery cohort reveals disturbances in fatty acid and lipid metabolism. *Mol Biosyst*. 2017;13(2):371-379.

###### Gu 2021

Gu SX, Tyagi T, Jain K, et al. Thrombocytopathy and endotheliopathy: crucial contributors to COVID-19 thromboinflammation. *Nat Rev Cardiol.* 2021;18(3):194-209. **Gualano 2011**

Gualano AB, Bozza T, Lopes De Campos P, et al. Branched-chain amino acids supplementation enhances exercise capacity and lipid oxidation during endurance exercise after muscle glycogen depletion. *J Sports Med Phys Fitness*. 2011;51(1):82 88.

###### Halpin 2020

Halpin SJ, McIvor C, Whyatt G, et al. Postdischarge symptoms and rehabilitation needs in survivors of COVID-19 infection: a cross-sectional evaluation. *J Med Virol*. 2020. **Howatson 2012**

Howatson G, Hoad M, Goodall S, et al. Exercise-induced muscle damage is reduced in resistance-trained males by branched chain amino acids: a randomized, double-blind, placebo controlled study. *J Int Soc Sports Nutr*. 2012;9:20.

**Holeček 2018**

Holeček M. Branched-chain amino acids in health and disease: metabolism, alterations in blood plasma, and as supplements. *Nutr Metab*. 2018;15:33.

###### Huang 2021

Huang L, Yao Q, Gu X, et al. 1-year outcomes in hospital survivors with COVID-19: a longitudinal cohort study. *Lancet*. 202 1;398(10302 ):747-758.

###### Jason et al 2011

Jason LA, Evans M, Brown M, et al. Fatigue scales and chronic fatigue syndrome: issues of sensitivity and specificity. *Disabil Stud Q.* 2011;31(1):1375.

###### Jayakumar 2019

Jayakumar S, Middleton MS, Lawitz EJ, et al. Longitudinal correlations between MRE, MRI PDFF, and liver histology in patients with non-alcoholic steatohepatitis: Analysis of data from a phase II trial of selonsertib. *J Hepatol*. 2019;70(1):133-141.

###### Kephart 2016

Kephart WC, Mumford PW, McCloskey AE, et al. Post-exercise branched chain amino acid supplementation does not affect recovery markers following three consecutive high intensity resistance training bouts compared to carbohydrate supplementation. *J Int Soc Sports Nutr*. 2016;13:30.

###### Kimball and Jefferson 2006

Kimball SR, Jefferson LS. Signaling pathways and molecular mechanisms through which branched-chain amino acids mediate translational control of protein synthesis. *J Nutr*.

2006;136(1 Suppl):227S-231S. **Komaroff and Lipkin 2021**

Komaroff AL, Lipkin WI. Insights from myalgic encephalomyelitis/chronic fatigue syndrome may help unravel the pathogenesis of postacute COVID-19 syndrome. *Trends Mol Med.* 2021;27(9):895-906.

###### Loomba 2019

Loomba R, Sanyal AJ, Kowdley KV, et al. Factors associated with histologic response in adult patients with nonalcoholic steatohepatitis. *Gastroenterology*. 2019;156(1):88-95.e5. **Loomba 2020**

Loomba R, Neuschwander-Tetri BA, Sanyal A, et al. Multicenter validation of association between decline in MRI-PDFF and histologic response in NASH. *Hepatology*. 2020;72(4):1219-1229.

###### Lopez-Leon 2021

Lopez-Leon S, Wegman-Ostrosky T, Perelman C, et al. More than 50 long-term effects of COVID-19: a systematic review and meta-analysis. *medRxiv*. (preprint) 2021;2021.01.27.21250617. doi:10.1101/2021.01.27.21250617.

###### Mantha et al 2020

Mantha S, Tripuraneni SL, Roizen MF, et al. Proposed modifications in the 6-minute walk test for potential application in patients with mild COVID-19: a step to optimize triage guidelines. *Anesth Analg.* 2020;131(2):398-402.

###### Medved 2004

Medved I, Brown MJ, Bjorksten AR, et al. N-acetylcysteine enhances muscle cysteine and glutathione availability and attenutates fatigue during prolonged exercise in endurance-trained individuals. *J Appl Physiol (1985)*. 2004;97(4):1477-1485.

###### Mittleman 1998

Mittleman KD, Ricci MR, Bailey SP. Branched-chain amino acids prolong exercise during heat stress in men and women. *Med Sci Sports Exerc*. 1998;30(1):83-91.

###### National Kidney Foundation Guidance 2019

National Kidney Foundation. Clinical practice guideline for nutrition in chronic kidney disease: 2019 update. https://www.kidney.org/sites/default/files/Nutrition_GL%2BSubmission_101719_Public_Review_Co py.pdf. Accessed August 2021.

###### Newsholme and Blomstrand 2006

Newsholme EA, Blomstrand E. Branched-chain amino acids and central fatigue. *J Nutr*. 2006;136(1 Suppl):274S-276S.

###### NICE Guideline 2021

National Institute for Health and Care Excellence. COVID-19 rapid guideline: managing COVID-19. September 2, 2021[. https://www.nice.org.uk/guidance/ng1](https://www.nice.org.uk/guidance/ng188)91; Accessed September 2021.

###### Patel 2016

Patel J, Bettencourt R, Cui J, et al. Association of noninvasive quantitative decline in liver fat content on MRI with histologic response in nonalcoholic steatohepatitis. *Therap Adv Gastroenterol*. 2016;9(5):692-701.

###### Paul 2021

Paul BD, Lemle MD, Komaroff AL, et al. Redox imbalance links COVID-19 and myalgic encephalomyelitis/chronic fatigue syndrome. *PNAS*. 2021;118(34):e2024358118. **Portier 2008**

Portier H, Chatard JC, Filaire E, et al. Effects of branched-chain amino acids supplementation on physiological and psychological performance during an offshore sailing race. *Eur J Appl Physiol*. 2008;104(5):787-794.

###### Prasun 2021

Prasun P. COVID-19: A mitocondrial perspective. *DNA Cell Biol*. 2021;40(6):713-719. **Ramakrishnan 2021**

Ramakrishnan RK, Kashour T, Hamid Q, et al. Unraveling the mystery surrounding post-acute sequelae of COVID-19. *Front Immunol*. 202 1;30(12 ):686029. **Reizine 2021**

Reizine F, Lesouhaitier M, Gregoire M, et al. SARS-CoV-2-induced ARDS associates with MDSC expansion, lymphocyte dysfunction, and arginine shortage. *J Clin Immunol*. 2021;2:1-11.

###### Riazi 2003

Riazi R, Wykes LJ, Ball RO, et al. The total branched-chain amino acid requirement in young healthy adult men determined by indicator amino acid oxidation by use of L-[1-13C]phenylalanine. *J Nutr*. 200 3;133(5 ):1383-1389.

###### Shi 2021

Shi D, Yan R, Lv L, et al. The serum metabolome of COVID-19 patients is distinctive and predictive. *Metabolism*. 2021;118:154739.

###### Shi and Puyo 2020

Shi Z, Puyo CA. N-acetylcysteine to combat COVID-19: an evidence review. *Ther Clin Risk Manag*. 2020;16:1047-1055.

###### Shimomura 2006

Shimomura Y, Yamamoto Y, Bajotto G, et al. Nutraceutical effects of branched-chain amino acids on skeletal muscle. *J Nutr*. 2006;136(2):529S-532S.

###### Townsend 2021

Townsend L, Dowds J, ’'Brien K, et al. Persistent poor health after COVID-19 is not associated with respiratory complications or initial disease severity. *Ann Am Thorac Soc*. 2021;18(6):997-1003.

###### Tsuda 2019

Tsuda Y, Yamaguchi M, Noma T, et al. Combined effect of arginine, valine, and serine on exercise-induced fatigue in healthy volunteers: a randomized, double-blinded, placebo-controlled crossover study. *Nutrients.* 2019;11(4):862. **VanDusseldorp 2018**

VanDusseldorp TA, Escobar KA, Johnson KE, et al. Effect of branched-chain amino acid supplementation on recovery following acute eccentric exercise. *Nutrients*. 2018;10(10):1389.

###### van Hall 1995

van Hall G, Raaymakers JS, Saris WH, et al. Ingestion of branched-chain amino acids and tryptophan during sustained exercise in man: failure to affect performance. *J Physiol.* 1995;486(Pt 3):789-794.

###### Vehar et al 2021

Vehar S, Boushra M, Ntiamoah P, et al. Post-acute sequela of SARS-CoV-2 infection: Caring for the “long-haulers.” Clev Clin J Med. 2021 May 3;88(5):267-272. **Wolfe 2017**

Wolfe RR. Branched-chain amino acids and muscle protein synthesis in humans: myth or reality? *J Int Soc Sports Nutr.* 2017;14:30.

###### Wong and Weitzer 2021

Wong TL, Weitzer DJ. Long COVID and myalgic encephalomyelitis/chronic fatigue syndrome (ME/CFS)-a systemic review and comparison of clinical presentation and symptomatology. *Medicina (Kaunas)*. 2021;57(5):418. doi:10.3390/medicina57050418. **Xiao 2021**

Xiao N, Nie M, Pang H, et al. Integrated cytokine and metabolite analysis reveals immunometabolic reprogramming in COVID-19 patients with therapeutic implications. *Nat Commun*. 2021;12:168.

###### Zane 2017

Zane AC, Reiter DA, Shardell M, et al. Muscle strength mediates the relationship between mitochondrial energetics and walking performance. *Aging Cell*. 2017;16(3):461-468. doi:

10.1111/acel.12568. Epub 2017 Feb 9.

**APPENDIX 1. SCHEDULE OF ASSESSMENTS - COHORT A**

|  | **Screenin**  **g** | **Treatment Period** | |  |  | **Follo w-up** |  |  |
| --- | --- | --- | --- | --- | --- | --- | --- | --- |
|  | **Visit 1** | **Visit 2 Baseline** | **Visit 3 (via Telephone)** | **Visit 4**  **EOT or ET** |  | **Visit 5**  **(via T elepho ne)** |  |  |
| **Day** | -28 to -1 | 1 | 14 | 28 | 35 | |  | For detail |
| **Week** | -4 to -1 | 0 | 2 | 4 | 5 | |  |  |
| **Window** |  |  | ±3 | ±3 | ±3 | |  |  |
| Informed consent | X |  |  |  |  | |  | 8.1.1 |
| Demographics | X |  |  |  |  | |  | 6.2 |
| Medical, surgical, and medication history | X |  |  |  |  | |  | 6.3 |
| Height, weight, and BMI | X |  |  |  |  | |  | 6.4 |
| Vital signs (BP, HR, RR, body temperature, and resting O_2_^a^) | X | X |  |  | X | |  | 6.6.2 |
| Echocardiogram^b^ | X |  |  |  |  | |  | 6.6.3 |
| Prior and/or concomitant medication | X | X | X |  | X | |  | 5.2 |
| Physical examination^c^ | X | X |  |  | X | |  | 6.6.1 |
| Magnetic resonance spectroscopy^d^ (^31^P-MRS and ^1^H-MRS) | X |  |  |  | X | |  | 6.8.1 |
| Blood and urine collection for local laboratory assessments^e^ | X | X |  |  | X | |  | 6.5;  APPENDIX 3 |
| Serum NT-pro BNP^f^ | X |  |  |  |  | |  | 6.10;  APPENDIX 3 |
| Alcohol breath test | X | X |  |  |  | |  | 6.6.4.3 |
| Blood/plasma collection for central laboratory assessments^g,h^ |  | X |  |  | X | |  | 6.5; 6.10;  APPENDIX 3 |
| Pregnancy testing (females with reproductive potential)^i^ | X | X |  |  | X | |  | 6.6.4.4 |
|  | **Screenin**  **g** | **Treatment Period** | | | **Follo w-up** | | |  |
|  | **Visit 1** | **Visit 2 Baseline** | **Visit 3 (via Telephone)** | **Visit 4**  **EOT or ET** | **Visit 5**  **(via T**  **elepho ne)** | | |  |
| **Day** | -28 to -1 | 1 | 14 | 28 | 35 | | |  |
| **Week** | -4 to -1 | 0 | 2 | 4 | 5 | | | For detail |
| **Window** |  |  | ±3 | ±3 | ±3 | | |  |
| 6-Minute Walk Test^j^ |  | X |  |  | X | | | 6.8.3,  SRM |
| Serum lactate^k^ |  | X |  |  | X | | | 6.8.4 |
| CFQ-11 PRO for fatigue^l^ | X | X | X |  | X | | | 6.8.2 |
| Treatment assignment |  | X |  |  |  | | | 5.1 |
| Study drug dispensing |  | X |  |  |  | | | 5.1.4 |
| Study drug self-administration^m^ |  | ← – – – – – – – – – – – – X – – – – – – – – – – – – _→_ | | |  | | | 5.1.3, 5.1.4 |
| Study drug compliance review |  |  | X n | X^n^ | X | | | 5.1.4 |
| AE assessment | X | X | X | X | X | | | 6.7 |

6MWT=6-minute walk test; AA=amino acid; AE=adverse event; BMI=body mass index; BNP=B-type natriuretic peptide; BP=blood pressure; CFQ=Chalder Fatigue Questionnaire; CHF=congestive heart failure; ECAR=extracellular acidification rate; EOT=End of Treatment; ET=early termination; FGF-21=fibroblast growth factor-21; ^1^H-MRS=proton magnetic resonance spectroscopy; HR=heart rate; LV=left ventricle; LVEF=left ventricular ejection fraction; MRS=magnetic resonance spectroscopy; NO=nitric oxide; OCR=oxygen consumption rate; PE=physical examination; PK=pharmacokinetics; ^31^P-MRS=phosphorus magnetic resonance spectroscopy; PRO=patient-reported outcomes; RR=respiration rate; SRM=Study Reference Manual. ^a^ To be performed prior to blood collection. Resting O2 saturation to rule out significant pulmonary disease as a cause of fatigue (must be ≥95% on room air)

^b^ To rule out significant tricuspid regurgitation, evidence of pulmonary hypertension, or any significant decrease in LVEF or LV wall abnormality consistent with CHF, unless documentation of a normal cardiac echocardiogram within 3 months of Screening and after at least 3 months of acute COVID-19 infection. Note: An echocardiogram is not required to be performed at Screening if documentation of a normal or not clinically significant echocardiogram was performed within 3 months of Screening and after at least 3 months of acute COVID-19 infection. ^c^ A full physical examination is performed at Screening to ensure eligibility. At Visits 2 and 4, a targeted symptom-driven examination can occur (eg, lungs, heart, or based on occurrence of symptoms or AEs).

1. Documented ^31^P-MRS within 3 months of Screening and after 3 months acute COVID-19 infection is acceptable. If a historical ^31^P-MRS has been used to determine eligibility, then ^31^P-MRS should be repeated within 1 month prior to Day 1 (ie, during the Screening Period) to confirm eligibility and establish baseline. Subjects are required to fast (water permitted) for a minimum of 6 hours prior to the MRS.
2. Complete blood count, chemistry, and routine urine analysis ^f^ To rule out significant CHF as a cause of fatigue (must be <400 pg/mL)
3. Change in circulating mitochondrial peptides (eg, Mots-C), metabolomics, proteomics, inflammation biomarkers, adhesion markers, muscle injury biomarkers (eg, troponins, creatine kinase, FGF-21), and mitochondrial function/metabolism markers in plasma (~1.5 mL total); NO biology; immune profiling, and metabolism/phenotypic ECAR/OCR; plasma AA profiling; and metabolomics. Plasma AA (PK samples) are to be collected no more than 30 minutes before administration of study drug and after a fast (minimum duration) of 10 hours.
4. Plasma metabolomic assays will be collected prior to and following moderate exercise.
5. A serum pregnancy test will be performed at Screening. A urine pregnancy test will be performed at Baseline and EOT/ET (Visit 4). ^j^ 6MWT should be performed at (or within 7 days of) Baseline and EOT/ET (Visit 4). The subject may have clinic visit assessments performed over 2 days. The subject will be provided an overnight stay at a nearby hotel.

^k^ A capillary blood lactate test by earlobe puncture is measured just before starting, immediately after and 5, 10, 20, and 30 minutes after the 6MWT. ^l^ At all visits other than Screening (Visit 1), it is to be given both before and after 6MWT. ^m^ The study drug should be self-administered at the clinic visit on Day 1 and Day 28 or EOT/ET. ^n^ At Visits 3 and 4, a compliance review will be performed.

**APPENDIX 2. SCHEDULE OF ASSESSMENTS - COHORT B**

|  | **Screenin**  **g** | **Treatment Period** | | | **Follow-up** |  |
| --- | --- | --- | --- | --- | --- | --- |
|  | **Visit 1** | **Visit 2**  **Baselin**  **e** | **Visit 3**  **(via Telephon**  **e)** | **Visit 4**  **EOT** | **Visit 5**  **(via Telephon**  **e)** | see Section |
| **Day** | -7 to -1 | 1 | 14 | 28 | 35 |  |
| **Week** | -1 | 0 | 2 | 4 | 5 |  |
| **Window** |  |  | ±3 | ±3 | ±3 |  |
| Informed consent | X |  |  |  |  | 8.1.1 |
| Demographics | X |  |  |  |  | 6.2 |
| Medical, surgical, and medication history | X |  |  |  |  | 6.3 |
| Height, weight, and BMI | X |  |  |  |  | 6.4 |
| Vital signs (BP, HR, RR, body temperature, and resting O_2_^a^) | X | X |  | X |  | 6.6.2 |
| Echocardiogram^b^ | X |  |  |  |  | 6.6.3 |
| Prior and/or concomitant medication | X | X | X | X | X | 5.2 |
| Physical examination^c^ | X | X |  | X |  | 6.6.1 |
| Blood and urine collection for local laboratory assessments^d^ | X |  |  | X |  | 6.5;  APPENDIX 4 |
| Serum NT-pro BNP^e^ | X |  |  |  |  | 6.10;  APPENDIX 4 |
| Alcohol breath test | X | X |  |  |  | 6.6.4.3 |
| Pregnancy testing (females with reproductive potential)^f^ | X | X |  | X |  | 6.6.4.4 |
| 6-Minute Walk Test^g^ |  | X |  | X |  | 6.8.3,  SRM |
| CFQ-11 PRO for fatigue^h^ | X | X | X | X |  | 6.8.2 |


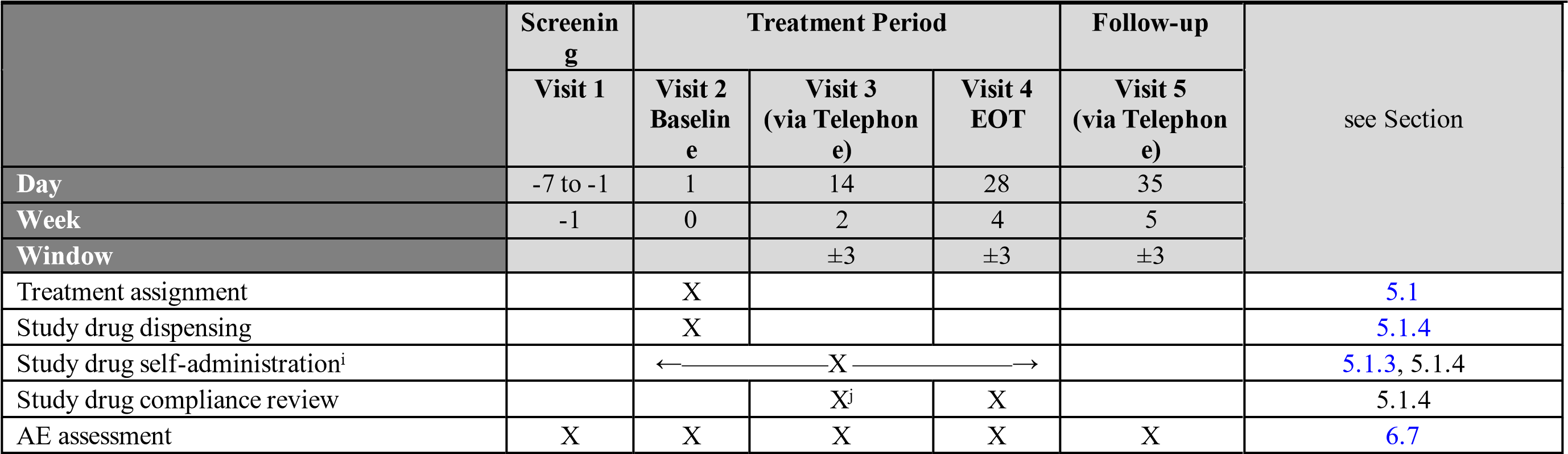


6MWT=6-minute walk test; AE=adverse event; BMI=body mass index; BNP=B-type natriuretic peptide; BP=blood pressure; CFQ=Chalder Fatigue Questionnaire; CHF=congestive heart failure; ECAR=extracellular acidification rate; EOT=End of Treatment; ET=early termination; HR=heart rate; LV= left ventricle; LVEF: left ventricle ejection fraction; OCR=oxygen consumption rate; PE=physical examination; PK=pharmacokinetics; PRO=patient-reported outcomes; RR=respiration rate; SRM=Study Reference Manual.

1. To be performed prior to blood collection. Resting O2 saturation to rule out significant pulmonary disease as a cause of fatigue (must be ≥95% on room air)
2. To rule out significant tricuspid regurgitation, evidence of pulmonary hypertension, or any significant decrease in LVEF or LV wall abnormality consistent with CHF, unless documentation of a normal cardiac echocardiogram within 3 months of Screening and after at least 3 months of acute COVID-19 infection. Note: An echocardiogram is not required to be performed at Screening if documentation of a normal or not clinically significant echocardiogram was performed within 3 months of Screening and after at least 3 months of acute COVID-19 infection. Optional depending on the results of Serum NT-pro BNP test.
3. A full physical examination is performed at Screening to ensure eligibility. At Visits 2 and 4, a targeted symptom-driven examination can occur (eg, lungs, heart, or based on occurrence of symptoms or AEs).
4. Complete blood count, chemistry, and routine urine analysis ^e^ To rule out significant CHF as a cause of fatigue (must be <400 pg/mL) ^f^ A serum pregnancy test will be performed at Screening. A urine pregnancy test will be performed at Baseline and EOT/ET (Visit 4). ^g^ 6MWT should be performed at Baseline and EOT/ET (Visit 4). The subject may have clinic visit assessments performed over 2 days. The subject will be provided an overnight stay at a nearby hotel.
5. At all visits other than Screening (Visit 1), it is to be given before 6MWT.
6. The study drug should be self-administered at the clinic visit on Day 1 and Day 28 (optional) or EOT/ET. ^j^ At Visits 3 and 4, a compliance review will be performed.

AXA1125 Protocol AXA1125-201

Version 4.0 03 August 2022

**APPENDIX 3. CLINICAL LABORATORY ANALYTES - COHORT A**

###### Screening Laboratories (Performed at the Local Laboratory)

| **Analytes** | **Urine Screen for Drugs of Abuse** |
| --- | --- |
| HBsAg (plasma)  HCV antibody (plasma)  HCV RNA [1] (plasma)  HIV antibody (plasma)  SARS-CoV-2 anti-N IgG (plasma) SARS-CoV-2 anti S IgG (plasma)  Serum hCG [2] FSH [3]  HbA1c | Amphetamines  Benzodiazepines  Cannabinoids  Cocaine  Methadone  Opioids |

FCBP=female(s) of childbearing potential; FSH=follicle-stimulating hormone; HBsAg=hepatitis B surface antigen; hCG=human chorionic gonadotropin; HCV=hepatitis C virus; HIV=human immunodeficiency virus; RNA=ribonucleic acid; SARS-CoV-2=severe acute respiratory syndrome coronavirus 2.

^^[[1]](#footnote-1)^^ HCV RNA will be tested in subjects with history of treated HCV infection or with positive HCV antibody during Screening provided that a negative hepatitis C viral load has not been documented at _≥_12 weeks after the cessation of hepatitis C treatment

1. Serum pregnancy test for FCBP only
2. For women <55 years of age or if amenorrheic for <12 months

###### Safety Laboratories (Performed at the Local Laboratory)

| **Hematology (Whole Blood)** | **Chemistry (Serum)** | **Urinalysis** |
| --- | --- | --- |
| Hemoglobin Hematocrit  RBC count  Platelet count  WBC count Neutrophils  Eosinophils  Monocytes Basophils  Lymphocytes  MCV  MCH MCHC MPV | Albumin  AST, ALT, ALP, GGT  Bilirubin (direct, total)  Blood urea nitrogen  Ca, Na, K, Cl, P, Mg Creatinine eGFR [1]  Lactate dehydrogenase bicarbonate Total protein Uric acid  Serum Lactate | pH  Glucose (qual)  Protein (qual)  Blood (qual)  Ketones  Microscopy (if dipstick positive)    **Other urine test** Urine hCG [2] |

###### Additional Laboratories (Performed at the Central Laboratory)

Total CO_2_

Nitrite Oxide Biology - NO

AAs

Note: Total CO_2,_ NO, AAs, will not be collected at Screening (Visit 1).

AA=amino acid; ALP=alkaline phosphatase; ALT=alanine aminotransferase; AST=aspartate aminotransferase; Ca=calcium; CKD-EPI=Chronic Kidney Disease Epidemiology Collaboration; Cl=chloride; CO_2_=carbon dioxide; eGFR=estimated glomerular filtration rate; FCBP=female(s) of childbearing potential; GGT=gamma-glutamyl transferase; hCG=human chorionic gonadotropin; K=potassium; MCH=mean corpuscular hemoglobin; MCHC=mean corpuscular hemoglobin concentration; MCV=mean corpuscular volume; Mg=magnesium; MPV=mean platelet volume; Na=sodium; NO=nitric oxide; P=phosphorus; qual=qualitative; RBC=red blood cell; WBC=white blood cell.

| AXA1125 |  | Protocol AXA1125-201 |
| --- | --- | --- |
|  |  | Version 4.0 03 August 2022 |

###### Plasma Biomarkers (Performed at the Central Laboratory)

| **Adhesion** | **Inflammation** | **Others (Local Laboratory)** |
| --- | --- | --- |
| ET1  E-selectin  VCAM-1  ICAM-1 | CRP (serum)  IL-6  MCP-1    **Others**    **Mitochondrial peptides**  Mots-C    **Muscle injury**  Troponins  Creatine kinase (serum)  FGF-21 | **Cardiac function**  Serum NT-pro BNP (serum) |

BNP=B-type natriuretic peptide; CRP=C-reactive protein; ET1=endothelin 1; FGF-21=fibroblast growth factor-21; ICAM-1=intercellular adhesion molecule-1; IL-6=interleukin-6; MCP-1=monocyte chemoattractant protein-1; VCAM-1=vascular cell adhesion molecule-1.

Axcella Health, Inc. Page 84 of 107

Confidential

**APPENDIX 4. SAFETY CLINICAL LABORATORY ANALYTES – COHORT B**

###### Screening Laboratories (Performed at the Local Laboratory)

| **Analytes** | **Urine Screen for Drugs of Abuse** |
| --- | --- |
| HBsAg (plasma)  HCV antibody (plasma)  HCV RNA [1] (plasma)  HIV antibody (plasma)  SARS-CoV-2 anti-N IgG (plasma)  SARS-CoV-2 anti S IgG (plasma) Serum hCG [2] FSH [3]  HbA1cHbA1c | Amphetamines  Benzodiazepines  Cannabinoids  Cocaine  Methadone  Opioids |

FCBP=female(s) of childbearing potential; FSH=follicle-stimulating hormone; HBsAg=hepatitis B surface antigen; hCG=human chorionic gonadotropin; HCV=hepatitis C virus; HIV=human immunodeficiency virus; RNA=ribonucleic acid; SARS-CoV-2=severe acute respiratory syndrome coronavirus 2.

1. HCV RNA will be tested in subjects with history of treated HCV infection or with positive HCV antibody during Screening provided that a negative hepatitis C viral load has not been documented at _≥_12 weeks after the cessation of hepatitis C treatment
2. Serum pregnancy test for FCBP only
3. For women <55 years of age or if amenorrheic for <12 months

AXA1125 Protocol AXA1125-201

Version 4.0 03 August 2022

###### Safety Laboratories (Performed at the Local Laboratory)

| **Hematology (Whole Blood)** | **Chemistry (Serum)** | **Urinalysis** |
| --- | --- | --- |
| Hemoglobin  Hematocrit RBC count  Platelet count  WBC count Neutrophils  Eosinophils Monocytes Basophils  Lymphocytes  MCV  MCH MCHC MPV | Albumin  AST, ALT, ALP, GGT  Bilirubin (direct, total)  Blood urea nitrogen  Ca, Na, K, Cl, P, Mg Creatinine eGFR [1]  Lactate dehydrogenase bicarbonate Total protein  Uric acid      **Cardiac Function**  Serum NT-pro BNP (serum) | pH  Glucose (qual) Protein (qual)  Blood (qual)  Ketones  Microscopy (if dipstick positive)    **Other urine test** Urine hCG [2] |

1 Calculated using the CKD-EPI equation 2 Urine pregnancy test is for FCBP only

ALP=alkaline phosphatase; ALT=alanine aminotransferase; AST=aspartate aminotransferase; BNP=B-type natriuretic peptide; Ca=calcium; CKD-EPI=Chronic Kidney Disease Epidemiology Collaboration; Cl=chloride; CO_2_=carbon dioxide; eGFR=estimated glomerular filtration rate; FCBP=female(s) of childbearing potential; GGT=gamma-glutamyl transferase; hCG=human chorionic gonadotropin; K=potassium; MCH=mean corpuscular hemoglobin; MCHC=mean corpuscular hemoglobin concentration; MCV=mean corpuscular volume; Mg=magnesium; MPV=mean platelet volume; Na=sodium; P=phosphorus; qual=qualitative; RBC=red blood cell; WBC=white blood cell.

Axcella Health, Inc.

Confidential

**APPENDIX 5. SUMMARY OF CHANGES**

###### Summary of Changes from Protocol V3.0 to V4.0

| **Section Number and Title** | **Original Text from v3.0 21December2021** | **Changed To in v4.0**  **01August2022** | **Rationale** |
| --- | --- | --- | --- |
| **Protocol Synopsis**    Investigator and  Study Sites | Original Text:  A minimum of one center is expected to enroll approximately 40 subjects. | Updated to read: A single site is expected to enroll approximately 40 subjects (Cohort A) and approximately 10 subjects (Cohort B). | The language in the text was changed to reflect 10  additional  participants will enroll in the  Cohort B study  extension. |
| **Protocol Synopsis**  Objectives    Section 2.2 | Original Text:    N/A | Added to read:  **Cohort B**  Assess the relationship between AXA1125 and functional status  Assess the safety and tolerability of  AXA1125 | The language in the text was changed to  differentiate  between the  study objectives  for Cohort A and  Cohort B study extension. |

AXA1125 Protocol AXA1125-201 Version 4.0 03 August 2022

| **Protocol Synopsis**    Section 2.3.2 | Original Text:  N/A | Added to read:  **2.3.2 Efficacy Endpoints -Cohort B**   - Change from baseline in subjects’ fatigue score as assessed by Chalder Fatigue Questionnaire (CFQ)-11 (by   Bimodal Scoring) before a  6MWT at Week 4, and at Day 14  (without 6MWT)   - The proportion of subjects with an improvement in fatigue score   as assessed by CFQ-11 before a  6MWT at Week 4   - Change from baseline in distance traveled during a 6MWT at Week 4 - The proportion of subjects with an improvement in distance traveled as assessed by 6MWT at Week 4 | The language was added to outline the efficacy endpoints for Cohort B study extension. |
| --- | --- | --- | --- |

Axcella Health, Inc.

Confidential

| **Protocol Synopsis**  Study Design      Section 3.1  Overall Study  Design | Original Text:    N/A | Added to read:  **Cohort B**  An additional cohort (approximately 10 subjects) will be added to the study which will include subjects who were considered screen-failures for Cohort A due to their PCr recovery rate constant (< 50 seconds),  provided their CFQ-11 is ≥8 and they meet  all the eligibility criteria  The total study duration for each subject in cohort B will be approximately 6 weeks, which will comprise a Screening Visit (1 week), Baseline Visit, a Treatment Period of 4 weeks, and a Follow-up Period of 1 week  After obtaining informed consent, subjects will be re-screened and approximately 10 eligible subjects will be randomized in a 1:1 ratio (double-blind, placebo-controlled) to receive either twice daily (BID) oral administration of 33.9 g AXA1125 or a placebo  Doses will be self-administered on Days 1 to 28, inclusive. Subjects will have clinic visits on Days 1, and 28, as well as telephone visits on Day 14, and 1 week after completion of study product administration.  The efficacy endpoints for Cohort B include the change from baseline at Week 4 in subjects’ fatigue score, as assessed by | The study design labels were added to differentiate between Cohort A and Cohort B and language is provided for an overview of the study design for Cohort B. |
| --- | --- | --- | --- |

|  |  | Chalder Fatigue Questionnaire (CFQ)-11 (by Bimodal Scoring) before 6 MWT, which will be evaluated at Screening, Baseline, and End of Treatment or Early Termination. CFQ-11 will also be assessed at Day 14 (without 6MWT). Additional endpoints for assessment of functional status, safety, and tolerability will be collected. Details of randomization will be provided in the Randomization Plan and Interactive Response Technology (IRT) system. |  |
| --- | --- | --- | --- |
| **Protocol Synopsis**    Number of Subjects | Original Text:    Approximately 40 subjects will be enrolled at 1 site in the United Kingdom. | Updated to read:  Approximately 40 subjects will be enrolled in Cohort A and 10 subjects will be enrolled in Cohort B at 1 site in the United Kingdom. | The language was updated to define the number of subjects targeted for Cohort B. |
| **Protocol Synopsis**    Inclusion Criteria | Original Text:    0. 4. A subject must have fatiguepredominant PASC defined by:  an elevated PCr recovery rate constant following moderate exercise on ^31^P-MRS (τ_PCr_  ≥50 seconds); and  Note: Documented 31P-MRS within 3 months of Screening | Updated to read:  4. A subject must have fatiguepredominant PASC defined by: **Cohort A Only**  an elevated PCr recovery rate constant following moderate exercise on ^31^P-MRS (τ_PCr_ ≥50 seconds); and Note: Documented 31P-MRS within 3 months of Screening and after 3 months of acute | The inclusion criteria was updated to differentiate between the entry criteria for  Cohort A and Cohort B. The criteria for the 31P-MRS (τPCr  ≥50 seconds) |

|  | and after 3 months of acute Coronavirus Disease 2019 (COVID-19) infection is acceptable. If a historical 31PMRS has been used to determine eligibility, then 31P-MRS should be repeated within 1 month prior to Day 1 (ie, during the Screening Period) to confirm eligibility and establish baseline. a baseline CFQ-11 physical fatigue score (by Bimodal Scoring) of ≥8. | Coronavirus Disease 2019 (COVID-19) infection is  acceptable. If a historical 31PMRS has been used to determine eligibility, then 31P-MRS should be repeated within 1 month prior to Day 1 (ie, during the Screening Period) to confirm eligibility and establish baseline. | was removed from Cohort B. |
| --- | --- | --- | --- |
| **Protocol Synopsis**    Duration of Study  Drug Exposure | Original Text:    Each subject will receive the study drug (AXA1125 or placebo) BID for up to 4 weeks. | Changed Text:    **Cohort A:** Each subject will receive the study drug (AXA1125 or placebo) BID for up to 4 weeks.  **Cohort B:** Each subject will receive the study drug (AXA1125 or placebo) BID for up to 4 weeks. | The language was added to clarify the duration of study drug exposure for Cohort A and Cohort B. |
| **Protocol Synopsis**    Statistical Methods | Original Text:  NA | Updated to read:    **Cohort B** | The interim analysis and data monitoring section is |

|  |  | Approximately 10 subjects (5 subjects per arm), who were considered screen-failures for Cohort A due to their PCr recovery rate constant (< 50seconds) provided their  CFQ-11 is ≥ 8 and they meet all the eligibility criteria will be enrolled in this Cohort.    **Interim Analyses and Data Monitoring**  **(Cohort A only)**  An interim analysis with potential sample size re-estimation will be performed when at least 10 subjects complete their Week 4 visit. Periodical administrative analyses may be performed to closely monitor the study. | applicable for Cohort A only.    This section clarifies that for Cohort B, 10 subjects who were previously screen failures due to their their PCr recovery rate constant (< 50seconds) provided their CFQ-11 is ≥ 8 will be analyzed. |
| --- | --- | --- | --- |
| Section 1.5.2.3.2 Studies in PASC | Original text:  No clinical studies of efficacy have been conducted in subjects with PASC. | Updated to read:  No clinical studies of efficacy have been conducted in subjects with PASC. An interim look at the data from Cohort A indicated no association between baseline fatigue score and baseline PCr.  Additionally, change in fatigue score did not correlate with change in PCr recovery rate utilizing bimodal scoring at Week 4. However, there was a statistically significant improvement in fatigue using CFQ-11 when assessed prior to 6MWT.  Accordingly, CFQ-11 was determined to be | The language was updated to include summary of the interim analysis findings from Cohort A. |

|  |  | an appropriate primary endpoint for the assessment of fatigue in Cohort B which includes patients who were considered screen-failures in Cohort A due to their PCr recovery rate constant (< 50 seconds), provided their CFQ-11 score is ≥8 and they meet all the eligibility criteria. |  |
| --- | --- | --- | --- |
| Section 1.6  Study Rationale | Original text:    NA | Updated to include:    An interim look at the data from Cohort A indicated no association between Baseline fatigue score and Baseline PCr. Additionally, statistically significant improvement in fatigue scores using CFQ11 prior to 6MWT did not correlate with changes in PCr recovery rate utilizing bimodal scoring at Week 4. Accordingly, CFQ-11 was determined to be appropriate primary end-point for the assessment of fatigue in Cohort B.  Moreover, in order to assess the relationship between AXA1125 and functional status, and to obtain valuable information on understanding the relationship between PCr and CFQ-11 results and AXA1125 responses, those subjects who were considered screen-failures in Cohort A, due to their PCr recovery rate constant (<50 seconds) provided their CFQ-11 Total score is ≥8 and they meet all the eligibility criteria for the study, will be included in a Cohort B | The language was updated to include the study rationale for adding the Cohort B extension. |

|  |  | to receive treatment for 4 weeks.  The efficacy endpoints for Cohort B include the change from Baseline at Week 4 in subjects’ fatigue score as assessed by CFQ11 before 6MWT, which will be evaluated at Screening, Baseline, and End of  Treatment or Early-Term. CFQ-11 will also be assessed at Day 14 (without 6MWT). Additional end-points for assessment of functional status, safety, and tolerability will be collected. |  |
| --- | --- | --- | --- |
| Section 3.1  Overall Study  Design    Study Schematic    Figure 2 | Original Text:  See above summary for Protocol Synopsis Study Design. | Updated to:    Study Schematic for Cohort B was added | The study schematic for Cohort B was added. |
| Section 4.4  Subject Enrollment | Original Text:    NA | Updated Text:    Approximately 40 subjects (Cohort A), and approximately10 subjects (Cohort B) are to be enrolled in this study. | The targeted number of subjects for Cohort B is defined. |
| Section 5  Study Intervention | Original Text:      Subjects are required to fast (water permitted) for a minimum of 6 hours prior to the MRS. | Updated Text:    Subjects are required to fast (water permitted) for a minimum of 6 hours prior to the MRS (Cohort A only). | The section clarifies that other restrictions are applicable to Cohort A only. |

| Section 5.5  Other Restrictions |  |  |  |
| --- | --- | --- | --- |
| Section 6.5  Laboratory Sample  Collections | Original Text:    The complete list of analytes to be tested are provided in APPENDIX 2. Detailed instructions for the preparation, handling, and storage of biological samples are provided in the Laboratory Manual. | Updated Text:    The complete list of analytes to be tested are provided in APPENDIX 3 (Cohort A) and APPENDIX 4 (Cohort B). Detailed instructions for the preparation, handling, and storage of biological samples are provided in the Laboratory Manual | The section clarifies that the blood and plasma collections for central laboratory are not collected in Cohort B.    The language  clarifies that the complete list of analytes to be tested for Cohort A can be found in Appendix 2 and for Cohort B Appendix 3. |
| Section 6.6.3  Echocardiogram    Appendix 2  Echocardiogramb | Original Text:  NA | Updated Text:  This test is optional depending on the Serum NT-pro BNP test results. | This section clarifies that for Cohort B the echocardiogram is optional depending on the Serum NTpro BNP test results. |
| Section 6.6.4.2 | Original Text: | Updated Text: | The language |

| Urine screen for  drugs of abuse          Section 6.6.4.3  Alcohol breath test | Urine will be collected at Screening and  Baseline for drugs of abuse listed in  APPENDIX 2.      An alcohol breath test will be performed at  Screening and Baseline, as specified in the SOA (APPENDIX 1). | Urine will be collected at Screening and  Baseline for drugs of abuse listed in APPENDIX 3 (Cohort A) and APPENDIX 4 (Cohort B).    An alcohol breath test will be performed at  Screening and Baseline, as specified in the SOA (APPENDIX 1) Cohort A and APPENDIX 2 (Cohort B). | was updated to clarify the urine drug screen for drugs of abuse and alcohol breath test are included in Appendix 2 for  Cohort A and Appendix 3 for Cohort B. |
| --- | --- | --- | --- |
| Section 6.8.2  Chalder Fatigue  Questionnaire (CFQ-11 PRO for fatigue) | Original Text:    Chalder Fatigue Questionnaire (CFQ-11 PRO for fatigue)  This self-administered questionnaire will be provided to the subject at Screening  (Visit 1), Visit 2, Visit 3, and EOT/ET (Visit 4). At Visit 3 (the telephone visit), it will be read to the subject or provided by secure electronic fashion. At all visits other than Screening, it will be performed before and after a 6MWT evaluation. | Updated Text:  Chalder Fatigue Questionnaire (CFQ-11 PRO for fatigue) (Cohort A and B)  This self-administered questionnaire will be provided to the subject at Screening (Visit 1), Visit 2, Visit 3, and EOT/ET (Visit 4). At Visit 3 (the telephone visit), it will be read to the subject or provided by secure electronic fashion. At all visits other than Screening, it will be performed before and after a 6MWT evaluation (Cohort A) and only before 6MWT evaluation (Cohort B). | The section clarifies that the Chalder Fatigue Questionnaire is collected for Cohort A and B.    The timing of collection for the collection of the  CFQ-11 during Cohort B is only  before the  6MWT  evaluation. |
| Section 7  Statistical  Considerations    Sample Size  Estimation | Original Text:  NA | Updated Text:    Cohort B  Approximately 10 subjects (5 subjects per arm), who were considered screen-failures for Cohort A due to their PCr recovery | The language was updated to clarify the sample size estimation for Cohort B. |
|  |  | rate constant (< 50seconds) provided their  CFQ-11 is ≥ 8 and they meet all the eligibility criteria will be enrolled in this Cohort. |  |
| Section 8.5 End of Study | Original Text:  For the purposes of this protocol, “end of study” is defined as the completion of the last subject visit. | Updated Text:  For the purposes of this protocol, “end of study” is defined as the completion of the last subject visit in both Cohort A and Cohort B. | The language was clarified to redefine the definition of the end of the study. |

###### Summary of Changes from Protocol V2.0 to V3.0

| **Section**  **Number and**  **Title** | **Original Text from v2.0 20October2021** | **Changed To in v3.0 10December2021** | **Rationale** |
| --- | --- | --- | --- |
| Section 4.1  Inclusion  Criteria | Inclusion Criterion #3, Original Text:  A subject must have had clinically suspected COVID-19 and a positive antibody test or a documented SARSCoV-2 infection (a positive reverse transcription polymerase chain reaction test) at least12 weeks prior to Screening. | Updated to read:  A subject must have had clinically suspected COVID-19 and a positive antibody test or a documented SARSCoV-2 infection (a positive reverse transcription polymerase chain reaction test) at least12 weeks prior to Screening. Note: Subjects who had a clinical diagnosis of COVID-19, but could not have a PCR test due to the need to selfisolate are allowed. | A note added to allow those subjects who had a clinical diagnosis of  COVID-19, but could not have a PCR test due to self-isolate to be included in the study |

| Section 4.1  Inclusion  Criteria | Inclusion Criterion #4, Original Text:  **1.** A subject must have fatiguepredominant PASC defined by:   - an elevated PCr recovery rate constant following moderate exercise on 31P-MRS (τ_PCr_ >50 seconds); and   Note: Documented 31P-MRS within 3 months of Screening and after 3 months of acute COVID-19 infection is acceptable. If a historical 31P-MRS has been used to determine eligibility, then 31PMRS should be repeated within 1 month prior to Day 1 (ie, during the Screening Period) to confirm eligibility and establish baseline.   - a baseline CFQ-11 physical fatigue score (by Bimodal   Scoring) of ≥8. | Updated to read:  A subject must have fatigue-predominant PASC defined by:   - an elevated PCr recovery rate constant following moderate exercise on 31P-MRS (τ_PCr_ ≥50 seconds); and   Note: Documented 31P-MRS within 3 months of Screening and after 3 months of acute COVID-19 infection is acceptable. If a historical 31P-MRS has been used to determine eligibility, then 31PMRS should be repeated within 1 month prior to Day 1 (ie, during the Screening Period) to confirm eligibility and establish baseline.   - a baseline CFQ-11 physical fatigue score (by Bimodal   Scoring) of ≥8. | The cut-off for the PCr recovery rate constant following moderate exercise on P-MRS was changed from (>50 seconds) to (≥50 seconds) to allow for more patients to be enrolled in the study. |
| --- | --- | --- | --- |
| Section 4.2  Exclusion  Criteria | Exclusion Criterion #4, Original Text:  Serum B-type natriuretic peptide (BNP)  >100 pg/mL | Updated to read:  Serum N terminal B-type natriuretic peptide  ( NT pro BNP) >400 pg/mL | The clinical range for BNP is being updated to match the appropriate range of the new assay (NT) pro BNP. No change in |

|  |  |  | the entry criteria for the study. |
| --- | --- | --- | --- |
| Section  6.7.4.2 Serious adverse event | Original Text:  All SAEs occurring from the time of informed consent until 4 weeks following the last administration of the study drug must be reported to Medpace Clinical Safety within 24 hours of the knowledge of the occurrence. After the 4-week reporting window, any SAE that the PI considers related to the study drug must be reported to Medpace Clinical Safety and the Sponsor/designee.  To report the SAE, complete the SAE form electronically in the eCRF. When the form is completed, Medpace Safety personnel will be notified electronically and will retrieve the form. If the event meets serious criteria and it is not possible to access the eCRF, send an email to Medpace Safety at **medpace-safetynotification@medpace.com** or call the Medpace SAE hotline (phone number listed below) and fax/email the completed paper SAE form to Medpace (contact information listed below) within 24 hours of awareness. When the eCRF becomes available, the SAE information must be entered within 24 hours of the system becoming available.  The PI must continue to follow up the subject until the SAE has subsided or until the condition becomes chronic in nature or | Updated to read:  Collection of SAEs will only be up to 7 days following the last dose, however, follow-up on ongoing SAEs will continue until resolution or 4 weeks following the administration of the last dose of the study drug. After the 7 days reporting window, any SAE that the PI considers related to the study drug must be reported to Medpace Clinical Safety and the Sponsor/designee.  To report the SAE, complete the SAE form electronically in the eCRF. When the form is completed, Medpace Safety personnel will be notified electronically and will retrieve the form. If the event meets serious criteria and it is not possible to access the eCRF, send an email to Medpace Safety at **medpace-safetynotification@medpace.com** or call the Medpace SAE hotline (phone number listed below) and fax/email the completed paper SAE form to Medpace (contact information listed below) within 24 hours of awareness. When the eCRF becomes available, the SAE information must be entered within 24 hours of the system becoming available.  The PI must continue to follow up the subject until the SAE has subsided or until the condition becomes chronic in nature or | The language in the text is updated to reflect the collection of SAEs will only be up to 7 days, instead of 4 weeks, following the last dose of the study drug as there is no scheduled visit 4 weeks after the last dose of the study drug. |

|  | stabilizes (in the case of persistent impairment) or the subject dies.  Within 24 hours of receipt of follow-up information, the PI must update the SAE form electronically in the eCRF and submit any supporting documentation (eg, subject discharge summary or autopsy reports) to Medpace Clinical Safety via fax or email. If it is not possible to access the eCRF, refer to the procedures outlined above for initial reporting of SAEs. | stabilizes (in the case of persistent impairment) or the subject dies.  Within 24 hours of receipt of follow-up information, the PI must update the SAE form electronically in the eCRF and submit any supporting documentation (eg, subject discharge summary or autopsy reports) to Medpace Clinical Safety via fax or email. If it is not possible to access the eCRF, refer to the procedures outlined above for initial reporting of SAEs. |  |
| --- | --- | --- | --- |
| Section 6.8.1 P-MRS | Original text:  31P-MRS and 1H-MRS acquisition protocol and guide is provided in Appendix 4 | Updated to read:  31P-MRS and 1H-MRS acquisition protocol and guide is provided in the Study reference Manual | The language was updated to reflect moving the P-MRS and H-MRS guide to the Study  Reference Manual removing from the Protocol Appendix |
| Section 6.8.3 6-Minute walk Test | Original text:  Detailed description of the 6-MWT is provided in Appendix 3. | Updated to read:  Detailed description of the 6-MWT is provided in the Study Reference Manual. | The language was updated to reflect moving the 6-MWT  description to the  Study Reference Manual and removing from the Protocol Appendix |

| Section 7.4.8 | Original Text:    The sponsor plans to perform a non-binding interim analysis when the first 10 subjects reach their Week 4 visit and when all the assessment results from that visit are available. Sample size re-estimation may be performed based on conditional power or predictive power derived from this interim analysis. | | |  | Updated to read:  An interim analysis with potential sample size re-estimation will be performed when a minimum of 10 subjects complete their Week 4 visit. Periodical administrative analyses may be performed to closely monitor the study. | | |  | The language in the text was changed to reflect more flexibility that the interim analysis will be performed when a minimum of 10 subjects complete their Week 4 visit. |
| --- | --- | --- | --- | --- | --- | --- | --- | --- | --- |
| Appendix 1  Schedule of  Assessment Footnotes f and k | Original text:  f. To rule out significant CHF as a cause of fatigue (must be < 100 pg/mL)    k. A capillary blood lactate test by earlobe puncture is measured just before starting, immediately after and 5, 10, 15, 20, and 30 minutes after the 6MWT. | | |  | Updated to read:  f. To rule out significant CHF as a cause of fatigue (must be < 400 pg/mL)    k. A capillary blood lactate test by earlobe puncture is measured just before starting, immediately after and 5, 10, 20, and 30 minutes after the 6MWT. | | |  | The cut-off for BNP was updated to match the new test NT-pro BNP.    The time points for capillary blood lactate were updated to match the same time points utilized in section 6.8.4 |
| Appendix 2  Screening  Laboratory  Analytes | Original text  **Screening Laboratories (Performed at the Local Laboratory)** | | |  | Updated to read  **Screening Laboratories (Performed at the Local Laboratory)** | | |  | The table was updated to reflect the correct name of the SARS-CoV-2 anti N IgG and SARS-CoV-2 anti S IgG performed at the local lab. |
|  |  | **Analytes** | **Urine Screen for Drugs of Abuse** |  |  | **Analytes** | **Urine Screen for Drugs of Abuse** |  |  |
|  |  | HBsAg (plasma)  HCV antibody  (plasma)  HCV RNA [1] (plasma) HIV antibody  (plasma) | Amphetamines  Benzodiazepines  Cannabinoids Cocaine  Methadone  Opioids |  |  | HBsAg (plasma)  HCV antibody  (plasma)  HCV RNA [1] (plasma) HIV antibody  (plasma) | Amphetamines  Benzodiazepines  Cannabinoids Cocaine  Methadone  Opioids |  |  |

|  |  | SARS-CoV-2 RNA  (plasma)  Serum hCG [2]  FSH [3] |  |  |  | SARS-CoV-2 anti-N  IgG (plasma)  SARS-CoV-2 anti-S  IgG (plasma) Serum hCG [2] FSH [3] |  |  |  |
| --- | --- | --- | --- | --- | --- | --- | --- | --- | --- |
| Appendix 2  Clinical  Laboratory  Analytes | Original Text:  **Safety Laboratories (Performed at the Local Laboratory)**  **Hematology Chemistry Urinalysis (Whole Blood) (Serum)**  Hemoglobin Albumin pH  Hematocrit AST, ALT, Glucose  RBC count ALP, GGT (qual)  Platelet count Bilirubin Protein (qual)  WBC count (direct, total) Blood (qual) Neutrophils Blood urea Ketones  Eosinophils nitrogen Microscopy  Monocytes Ca, Na, K, Cl, (if dipstick  Basophils P, Mg positive)  Lymphocytes Creatinine  MCV eGFR [1] **Other urine**  MCH Lactate **test**  MCHC dehydrogenase Urine hCG  MPV Total CO_2_ [2]  (central lab) bicarbonate Total protein Uric acid NO (central lab) AAs (central lab) | | | | Updated to:  **Safety Laboratories (Performed at the Local Laboratory)**  **Hematology Chemistry Urinalysis (Whole Blood) (Serum)**  Hemoglobin Albumin pH  Hematocrit AST, ALT, Glucose  RBC count ALP, GGT (qual)  Platelet count Bilirubin Protein (qual)  WBC count (direct, total) Blood (qual) Neutrophils Blood urea Ketones  Eosinophils nitrogen Microscopy  Monocytes Ca, Na, K, Cl, (if dipstick  Basophils P, Mg positive)  Lymphocytes Creatinine  MCV eGFR [1] **Other urine**  MCH Lactate **test**  MCHC dehydrogenase Urine hCG  MPV bicarbonate [2]  Total protein  Uric acid            **Safety Laboratories (Performed at the**  **Central Laboratory)** | | | | Table was adjusted  to reflect: Total CO_2_ , NO, and AAs were removed from safety labs and listed separately to be performed at Central lab and not to be collected at the screening visit (Visit 1).    Table of Plasma Biomarkers was updated to reflect that Mitochondrial peptides and MotsC will be performed at Central lab. The cardiac function test was also updated from Serum BNP to  Serum NT-proBNP. |

|  | **Plasma Biomarkers (Performed at the**   \| **Adhesion** \| **Inflammation** \| **Others (Local Laboratory)** \| \| --- \| --- \| --- \| \| ET1  E-selectin  VCAM-1  ICAM-1 \| CRP (serum)  IL-6  MCP-1 \| **Mitochondrial peptides**  Mots-C    **Cardiac function** Serum BNP  (serum)    **Muscle injury**  Troponins Creatine kinase (serum)  FGF-21 \|   **Central Laboratory)** | Total CO_2_  Nitrite Oxide Biology - NO  AAs    Note: Total CO_2,_ NO, AAs, will not be collected at Screening (Visit 1).                **Plasma Biomarkers (Performed at the**   \| **Adhesion** \| **Inflammation** \| **Others (Local Laboratory)** \| \| --- \| --- \| --- \| \| ET1  E-selectin  VCAM-1 ICAM-1 \| CRP (serum)  IL-6  MCP-1    **Others**    **Mitochondrial peptides**  Mots-C \| **Cardiac function** Serum NT-pro  BNP (serum)    **Muscle injury**  Troponins Creatine kinase (serum)  FGF-21 \|   **Central Laboratory)** |  |
| --- | --- | --- | --- | --- | --- | --- | --- | --- | --- | --- | --- | --- | --- | --- | --- |

###### Summary of Changes from Protocol V1.0 to V2.0

| **Section Number**  **and Title** | **Original Text from v1.0 24September2021** | **Changed To in v2.0**  **20October2021** | **Rationale** |
| --- | --- | --- | --- |
| Section 4.1 Inclusion Criteria | Inclusion Criterion #5, Original Text: Other than PASC, a subject must be in good health with no other major uncontrolled conditions. Allowable conditions include mild hypertension, dyslipidemia, and/or asthma (mild and not requiring chronic daily treatment). Subjects who are treated for these conditions must be well controlled on a stable regimen (lifestyle modifications and/or medications), for at least 3 months prior to Screening and anticipate no significant alterations to these regimens for the duration of the study. However, doses of certain medications (eg, statins, antihypertensives) used to treat stable chronic conditions may be modified during the study for safety or tolerability issues, if needed at the discretion of the Principal Investigator (PI).  Note: A history of treated hepatitis C virus (HCV) infection | Updated to read:  Other than PASC, a subject must be in good health without other significant medical or not well controlled medical or psychiatric conditions. Allowable conditions include mild hypertension, dyslipidemia, pre-diabetes as defined as HbA1c ≤ 6.0% controlled by diet, and/or asthma (mild and not requiring chronic daily treatment). Subjects who are treated for these conditions must be well controlled on a stable regimen (lifestyle modifications and/or medications), for at least 3 months prior to Screening and anticipate no significant alterations to these regimens for the duration of the study. However, doses of certain medications (eg, statins, antihypertensives) used to treat stable chronic conditions may be modified during the study for safety or tolerability issues, if needed at the discretion of the | Language updated to clarify the  intent of the inclusion criteria based on feedback from the MHRA during CTA review. |

|  | is allowed, provided that a negative hepatitis C viral load has been documented at ≥12 weeks after the cessation of hepatitis C treatment. Historical medical record or testing to ensure a negative viral load may be permitted during the Screening Period. HCV ribonucleic acid will be tested in all subjects, including subjects with a history of treated HCV infection or with positive HCV antibody at Screening, provided that a negative hepatitis C viral load has  not been documented at ≥12 weeks after the cessation of hepatitis C treatment. | Principal Investigator (PI).  Note: A history of treated hepatitis  C virus (HCV) infection is allowed, provided that a negative hepatitis C viral load has been documented at ≥12 weeks after the cessation of hepatitis C treatment. Historical medical record or testing to ensure a negative viral load may be permitted during the Screening Period. HCV ribonucleic acid will be tested in all subjects, including subjects with a history of treated HCV infection or with positive HCV antibody at Screening, provided that a negative hepatitis  C viral load has not been  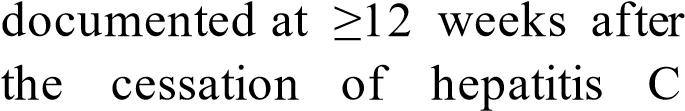treatment. |  |
| --- | --- | --- | --- |
| Section 4.2  Exclusion Criteria | Exclusion Criterion #4, Original Text: Meet any of the following laboratory parameters:   - Serum B-type natriuretic peptide   >100 pg/mL   - Total bilirubin >1.3 mg/dL or direct bilirubin >0.40 mg/dL   unless with a history of Gilbert’s  syndrome | Updated to read:  Meet any of the following laboratory parameters:   - Serum B-type natriuretic peptide   >100 pg/mL   - Total bilirubin >1.3 mg/dL or direct bilirubin >0.40 mg/dL   unless with a history of Gilbert’s  syndrome | Updated thresholds for AST/ALT and HbA1c based on feedback from the MHRA during CTA review. |
|  | - Aspartate aminotransferase (AST) or alanine aminotransferase (ALT) >5 × upper limit of normal   (ULN)   - Glycated hemoglobin (HbA1c)   >6.5%   - Human immunodeficiency virus   (HIV)-1 or -2 positive   - Estimated glomerular filtration rate <60 mL/min/1.73 m2 calculated using the Chronic Kidney Disease Epidemiology Collaboration equation - Positive for hepatitis B surface antigen (HBsAg), HCV antibody, or HIV antibody | - Aspartate aminotransferase (AST) or alanine aminotransferase (ALT)   >3 × upper limit of normal (ULN)   - Glycated hemoglobin (HbA1c)   >6.0   - Human immunodeficiency virus   (HIV)-1 or -2 positive   - Estimated glomerular filtration rate <60 mL/min/1.73 m2 calculated using the Chronic Kidney Disease Epidemiology Collaboration equation - Positive for hepatitis B surface antigen (HBsAg), HCV antibody, or HIV antibody |  |
| Appendix 1 Schedule of assessments | --- | Added collection of adverse events (AEs) at the screening visit. | Revised AE collection based on feedback from the MHRA during CTA review. |

1. Calculated using the CKD-EPI equation ^2^ Urine pregnancy test is for FCBP only.

   Axcella Health, Inc. Page 83 of 107

   Confidential [↑](#footnote-ref-1)
