# Supplementary material for: Efficacy and tolerability of an endogenous metabolic modulator (AXA1125) in fatigue-predominant long COVID: a single-centre, double-blind, randomised controlled phase 2a pilot study
Source: eClinicalMedicine. 2023 Apr 14;59:101946. doi: 10.1016/j.eclinm.2023.101946 (PMC10102537; doi:10.1016/j.eclinm.2023.101946)
Supplement: Statistical analysis plan [file mmc3.pdf]

Protocol AXA1125-201

Randomized, Double-Blind, Placebo-Controlled Pilot Study to Evaluate the Efficacy, Safety, and Tolerability of AXA1125 in Subjects With Fatigue-Predominant Post-Acute Sequelae of SARS-CoV-2 (PASC) Infection

**Protocol Number:**<br>**(Version Date)**	3.0<br>21DEC2021
**Name of Test Drug:**	AXA1125
**Phase:**	Pilot Study
**Methodology:**	Randomized, Double-Blind, Placebo-Controlled
**Sponsor:**	Axcella Health, Inc.<br>840 Memorial Drive<br>Cambridge, MA 02139<br>United States<br>Tel: +1-857-320-2200
**Sponsor Representative:**	Jeff Zhao<br>Sr. Director, Biometrics
**Document Date:**	01-JUN-2022
**Document Version:**	1.0

Axcella Health Inc./ Cytel Confidential: This is a controlled document and shall not be changed, saved, copied or reproduced by any unauthorized person.

SIGNATURE PAGE

**Protocol Title:**	A Randomized, Double-Blind, Placebo-Controlled Pilot Study to Evaluate the Efficacy, Safety, and Tolerability of AXA1125 in Subjects With Fatigue-Predominant Post-Acute Sequelae of SARS-CoV-2 (PASC) Infection
**Sponsor:**	Axcella Health, Inc.<br>840 Memorial Drive<br>Cambridge, MA 02139<br>United States<br>Tel: +1-857-320-2200
**Protocol Number:**	AXA1125-201
**Document Date/Version:**	01 JUN 2022 / v1.0

Cytel, Inc. Author:
Lin Yang
Cytel, Inc.
675 Massachusetts Avenue
Cambridge, MA 02139

Signature: Lyang

Electronically signed by: Lin Yang
Reason: Approve
Date: Jun 1, 2022 11:08 EDT

Email: lin.yang@cytel.com

Sponsor Approval

By signing this document, I acknowledge that I have read the document and approve of the planned statistical analyses described herein. I agree that the planned statistical analyses are appropriate for this study, are in accordance with the study objectives, and are consistent with the statistical methodology described in the protocol, clinical development plan, and all applicable regulatory guidance's and guidelines.

I have discussed any questions I have regarding the contents of this document with the biostatistical author.

I also understand that any subsequent changes to the planned statistical analyses, as described herein, may have a regulatory impact and/or result in timeline adjustments. All changes to the planned analyses will be described in the clinical study report (CSR).

Sponsor Signatory:
Jeff Zhao
Axcella Health, Inc.
840 Memorial Drive
Cambridge, MA 02139
United States

Signature: Jeff Zhao

Electronically signed by: Jeff Zhao
Reason: Approve
Date: Jun 2, 2022 14:02 EDT

Email: jzhao@axcellatx.com

Axcella Health Inc./ Cytel Confidential: This is a controlled document and shall not be changed, saved, copied or reproduced by any unauthorized person.

MODIFICATION HISTORY

Current Version	Date	Amended by	Summary of Changes from previous version	Reason
1.0	01-JUN-2022	NA	Original document	NA

Axcella Health Inc./ Cytel Confidential: This is a controlled document and shall not be changed, saved, copied or reproduced by any unauthorized person.

Table of Contents

1 INTRODUCTION AND OBJECTIVES OF ANALYSIS 9
1.1 INTRODUCTION 9
1.2 OBJECTIVES 9
1.2.1 Primary objective 9
1.2.2 Secondary objectives 9
1.2.3 Exploratory objectives 9
2 STUDY DESIGN 10
2.1 INTRODUCTION 10
2.2 RANDOMIZATION METHODOLOGY 10
2.3 STOPPING RULES 10
2.4 BLINDING 11
2.5 INTERIM ANALYSES 11
3 STUDY ENDPOINTS 12
3.1 EFFICACY VARIABLES 12
3.2 SAFETY VARIABLES 15
4 ANALYSIS SETS 16
4.1 ANALYSIS SET DEFINITIONS 16
4.2 PROTOCOL DEVIATIONS 16
5 DATA HANDLING 17
5.1 COMPUTING ENVIRONMENT 17
5.2 METHODS OF POOLING DATA 17
5.3 WITHDRAWALS, DROPOUTS, LOSS TO FOLLOW-UP 17
5.4 VISIT WINDOWS 17
6 STATISTICAL METHODS 18
6.1 SAMPLE SIZE JUSTIFICATION 18
6.2 GENERAL STATISTICAL METHODS 18
6.2.1 General Methods 18
6.2.2 Definition of Baseline 18
6.2.3 Adjustments for Covariates 18
6.2.4 Multiple Comparisons/Multiplicity 18
6.2.5 Subgroups 19
6.2.6 Missing, Unused, and Spurious Data 19
6.3 STUDY POPULATION 19
6.3.1 Subject Disposition 19
6.3.2 Demographic and Baseline Characteristics 20

Axcella Health Inc./ Cytel Confidential: This is a controlled document and shall not be changed, saved, copied or reproduced by any unauthorized person.

6.3.3 Extent of Exposure 20
6.4 EFFICACY EVALUATION 20
6.4.1 Primary Efficacy Analysis 20
6.4.2 Secondary/Exploratory Analysis 21
6.5 SAFETY EVALUATIONS 21
6.5.1 Adverse Events 21
6.5.2 Laboratory Data 22
6.5.3 Vital Signs and Physical Examinations 23
6.5.4 Echocardiogram 23
6.5.5 Concomitant Medications 23
7 CHANGES TO PLANNED ANALYSES 24
8 REFERENCES 25
9 APPENDICES 26
9.1 SCHEDULE OF ASSESSMENTS 26

Axcella Health Inc./ Cytel Confidential: This is a controlled document and shall not be changed, saved, copied or reproduced by any unauthorized person.

List on in-text Tables

Page

NO TABLE OF FIGURES ENTRIES FOUND.

Axcella Health Inc./ Cytel Confidential: This is a controlled document and shall not be changed, saved, copied or reproduced by any unauthorized person.

ABBREVIATIONS

Abbreviation	Definition
6MWD	6-minute walk test distance
6MWT	6-minute walk test
AA	Amino acid
ADP	Adenosine diphosphate
AE	Adverse event
ANCOVA	Analysis of covariance
ATC	Anatomic Therapeutic Class
BID	Twice daily
BMI	Body mass index
BNP	B-type natriuretic peptide
CFQ	Chalder Fatigue Questionnaire
COVID-19	Coronavirus Disease 2019
CSR	Clinical Study Report
CTCAE	Common Terminology Criteria for Adverse Events
DBL	Database Lock
Echo	Echocardiogram
EOT	End of Treatment
ET	Early Termination
FGF-21	Fibroblast growth factor-21
^{1}H-MRS	Proton magnetic resonance spectroscopy
IA	Interim Analysis
ICH	International Council on Harmonization
IRT	Interactive Response Technology
ITT	Intent-to-treat
MedDRA	Medical Dictionary for Regulatory Activities
MRS	Magnetic resonance spectroscopy
PASC	Post-acute sequelae of SARS-CoV-2 infection
PCr	Phosphocreatine
PI	Principal Investigator
^{31}P-MRS	Phosphorus magnetic resonance spectroscopy
PP	Per-protocol
PPS	Per-protocol analysis set
PT	Preferred Term
SAE	Serious adverse event
SAF	Safety Analysis Set
SAP	Statistical analysis plan

Axcella Health Inc./ Cytel Confidential: This is a controlled document and shall not be changed, saved, copied or reproduced by any unauthorized person.

**Abbreviation**	**Definition**
SARS-CoV-2	Severe acute respiratory syndrome coronavirus 2
SD	Standard deviation
SI	International System of Units
SOC	System Organ Class
TEAE	Treatment-emergent adverse event
WHO	World Health Organization

Axcella Health Inc./ Cytel Confidential: This is a controlled document and shall not be changed, saved, copied or reproduced by any unauthorized person.

1 INTRODUCTION AND OBJECTIVES OF ANALYSIS

1.1 Introduction

This document presents the statistical analysis plan (SAP) for Axcella Health Inc., AXA1125-201 study.

The statistical analysis plan (SAP) is based on the protocol version 3.0 dated 21st December 2021 and is designed to outline the methods to be used in the analysis of study data to answer the study objectives. Populations for analysis, data handling rules, statistical methods, and formats for data presentation are provided. The statistical analyses and summary tabulations described in this SAP will provide the basis for the results sections of the clinical study report (CSR) for this trial.

Post-hoc exploratory analyses not necessarily identified in this SAP may be performed to further examine study data. Such post-hoc analyses will be clearly identified in the final CSR.

1.2 Objectives

1.2.1 Primary objective

The primary objective is to:

- Assess the impact of AXA1125 on muscle function (metabolism) following exercise

1.2.2 Secondary objectives

The secondary objectives are to:

- Assess the relationship between AXA1125 and functional status
- Assess the safety and tolerability of AXA1125

1.2.3 Exploratory objectives

The exploratory objectives are to:

- Obtain additional insights into the mechanism of action of AXA1125
- Obtain baseline amino acid (AA) profile in subjects with post-acute sequelae of severe acute respiratory syndrome coronavirus 2 infection (PASC)

Axcella Health Inc./ Cytel Confidential: This is a controlled document and shall not be changed, saved, copied or reproduced by any unauthorized person.

2 STUDY DESIGN

2.1 Introduction

This is a minimum of one center, randomized, double-blind, placebo-controlled, pilot clinical study. The total study duration for each subject will be approximately 9 weeks. This study will comprise a Screening Period of up to 4 weeks, a Treatment Period of 4 weeks, and a Follow-up Period of 1 week. After obtaining informed consent, subjects will be screened, and approximately 40 eligible subjects (approximately 20 subjects per arm) will be randomized in a 1:1 ratio to receive twice daily (BID) oral administration of 33.9 g AXA1125 or a placebo, as shown in Figure 1. AXA1125-201 Study SchemaFigure 1.

Figure 1. AXA1125-201 Study Schema

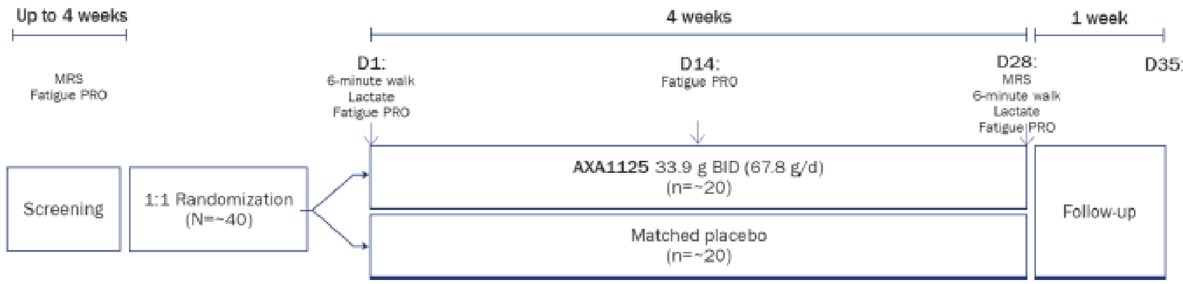

BID=twice daily; D=Day; MRS=magnetic resonance spectroscopy; PRO=patient-reported outcomes.

Doses will be self-administered on Days 1 to 28, inclusive. Subjects will have clinic visits on Day 1 and Day 28, as well as telephone visits on Day 14 and Day 35 (1 week after completion of study drug administration).

2.2 Randomization Methodology

At Visit 2, eligible subjects will be randomized through an IRT system into 1 of 2 groups (33.9 g BID AXA1125 or placebo BID) at a treatment allocation ratio of 1:1.

Details of randomization are provided in the separate Randomization Plan and Interactive Response Technology (IRT) system.

Axcella Health Inc./ Cytel Confidential: This is a controlled document and shall not be changed, saved, copied or reproduced by any unauthorized person.

2.3 Stopping Rules

There are no pre-specified stopping rules defined in the protocol. Nevertheless, there is an interim analysis planned where the unblinded Sponsor Statistician will review intermediate Safety and Efficacy data.

2.4 Blinding

The subject, investigators, study site personnel involved in direct care of the subject will remain blinded to individual study treatments. Personnel involved in the laboratory and image analysis will also remain blinded to the treatment assignment.
The Sponsor study team members will remain blinded to the treatment assignments during the study until the final Database Lock (DBL).
A Sponsor biostatistician will be unblinded to perform the interim analysis and data monitoring.

2.5 Interim Analyses

An interim analysis (IA) will be performed when a minimum of 10 subjects completes their Week 4 visit. This IA will be performed by the Sponsor Unblinded Statistician.

Axcella Health Inc./ Cytel Confidential: This is a controlled document and shall not be changed, saved, copied or reproduced by any unauthorized person.

3 STUDY ENDPOINTS

3.1 Efficacy Variables

The following efficacy variables will be analyzed to address the primary, secondary, and exploratory objectives:

Primary

- The absolute change from baseline at Week 4 in the phosphocreatine (PCr) recovery rate following moderate exercise, as assessed by ^{31}P-magnetic resonance spectroscopy (^{31}P-MRS)
  - Change from baseline at Week 4 is assessed as the difference between the Stress PCr recovery rate time constant (seconds) at Week 4 and at Baseline, as collected in the CRF.

Secondary

- Relative change from baseline in PCr recovery rate as assessed by ^{31}P-MRS at Week 4
  - Relative change from baseline at Week 4 is assessed as the percentage of the absolute change in the Stress PCr recovery rate time constant (seconds) at Week 4 and at Baseline divided by baseline value.
- The proportion of subjects with improvement in PCr recovery rate at Week 4
  - The proportion of subjects with improvement of 10-second in Stress PCr recovery rate constant time (seconds) at Week 4 from baseline.
  - The proportion of subjects in PCr recovery rate time constant ≤ 45 seconds at Week 4
  - The proportion of subjects in PCr recovery rate time constant < 50 seconds at Week 4
- Absolute and relative change from baseline in peak serum lactate level after a 6-minute walking test (6MWT) at Week 4
  - Evaluated for the highest post-6MWT serum lactate level at Baseline and Week 4.
- The proportion of subjects with peak serum lactate level ≤3 mmol/L after a 6MWT at Week 4
  - Evaluated for the highest post-6MWT serum lactate level at Week 4.
- The proportion of subjects with a decrease in peak serum lactate level from baseline after a 6MWT at Week 4
  - Decrease is defined as a strictly decrease from baseline in the highest post-6MWT serum lactate level at Week 4.
- Change from baseline in distance traveled during a 6MWT at Week 4
  - Absolute change from baseline at Week 4 is assessed as the difference between the total 6MWT distance (6MWD) walked (meters) at Week 4 and at Baseline, as collected in the CRF.

Axcella Health Inc./ Cytel Confidential: This is a controlled document and shall not be changed, saved, copied or reproduced by any unauthorized person.

  - Relative change from baseline at Week 4 is assessed as the percentage of the absolute change in the total 6MWT distance (6MWD) walked (meters) at Week 4 and at Baseline divided by baseline value.
  - Absolute and Relative change from baseline in percent predicted 6MWD at Week 4. The percent predicted 6MWD is computed as 100*(observed 6MWD / predicted 6MWD) where predicted 6MWD (meters) is calculated by gender as follows:
    - Men: (7.57 × height) – (5.02 × age) – (1.76 × weight) – 309
    - Women: (2.11 × height) – (5.78 × age) – (2.29 × weight) + 667
    - Height is expressed in cm, age in years and weight in kg

- Change from baseline in subjects' fatigue score as assessed by Chalder Fatigue Questionnaire (CFQ)-11 (by Bimodal Scoring and Likert Scoring) before and after a 6MWT at Week 4 and at Day 14 (without 6MWT)
  - Fatigue score will be computed as the total score and two subscales: physical fatigue and mental fatigue. The total score is computed as the sum of the score for all 11 individual questions. The physical fatigue score is measured by question 1- 7; the mental fatigue is measured by question 8 –11. The scales for responses using the bimodal scoring and Likert scoring are listed below:

**Scoring schemes for the Chalder Fatigue Scale**		
Response	Bimodal score	Likert score
Less than usual / Better than usual	0	0
No more than usual/ No Worse than usual	0	1
More than usual / Worse than usual	1	2

Axcella Health Inc./ Cytel Confidential: This is a controlled document and shall not be changed, saved, copied or reproduced by any unauthorized person.

Much more than usual / Much worse than usual	1	3

Fatigue total score in bimodal scoring has a value range of 0-11; the fatigue total score in Likert scoring has a value range of 0-33. If any question is missing, the fatigue total score will be considered as missing. If some questions are missing, but the questions within the domain (physical or mental) are completed, then the subscale for this domain will be calculated. .

- Following Absolute and Relative changes in fatigue score with Bimodal and Likert scoring including total score, physical score and mental score will be computed:
  - The change in score pre- 6MWT between Baseline at Week 4.
  - The change in score post-6MWT between Baseline at Week 4.
  - The change in the change between pre- 6MWT and post- 6MWT between Baseline and Week 4.
  - The change between pre- 6MWT at Baseline and Day 14 (no 6MWT).
  - The change between post- 6MWT at Baseline and Day 14 (no 6MWT).

- The proportion of subjects with an improvement in fatigue score including total score, physical score and mental score as assessed by CFQ-11 with Bimodal and Likert scoring before and after a 6MWT from baseline at Week 4
  - Improvement is defined as a strictly decrease in CFQ-11 fatigue score from pre-6MWT Baseline at pre-6MWT Week 4.
  - Improvement is defined as a strictly decrease in CFQ-11 fatigue score from post-6MWT Baseline at post-6MWT Week 4.
  - Improvement is defined as a strictly decrease difference of the change in CFQ-11 fatigue score between pre-6MWT and post-6MWT between Baseline and Week 4.
  - For the fatigue subjects defined by using overall CFQ bimodal fatigue score with a cut-off at < 4 (non-fatigue) vs. ≥ 4 (fatigue) at baseline,
    - Improvement is defined as the change in fatigue status pre-6MWT at baseline from fatigue to non-fatigue pre-6MWT at Week 4.
    - Improvement is defined as the change in fatigue status post-6MWT at baseline from fatigue to non-fatigue status post-6MWT at Week 4.

Exploratory

- Absolute and/or relative changes from baseline will be assessed at Week 4 for various exploratory biomarkers including circulating mitochondrial peptide Mots-C, inflammatory

Axcella Health Inc./ Cytel Confidential: This is a controlled document and shall not be changed, saved, copied or reproduced by any unauthorized person.

serum and plasma proteins, endothelial adhesion markers, muscle injury (eg, troponins, creatine kinase, fibroblast growth factor-21) biomarkers, and nitric oxide synthetic enzymes. Untargeted metabolomics and proteomics will also be run.

- Absolute and Relative change from baseline in Rest PCr concentration, Rest intramyocellular pH, Rest adenosine diphosphate concentration, Stress end of exercise PCr concentration, Stress end of recovery PCr concentration, Stress drop in PCr concentration during exercise, Stress end of exercise intramyocellular pH, Stress end of exercise adenosine diphosphate (ADP) concentration, Stress initial recovery rate of PCr, Stress maximal oxidative capacity, Rest 1H creatine concentration, Rest 1H intramyocellular lipid content, Rest 1H acetyl-carnitine concentration, Rest 1H carnosine concentration at Week 4

3.2 Safety Variables

- AEs and serious adverse events (SAEs)
    - The frequency of treatment emergent AEs (TEAE), defined as any AE which begins or worsens in severity after the first dose of study drug is taken
    - The frequency of TEAEs by CTCAE severity grade
    - The frequency of TEAEs related to study treatment
    - The frequency of TEAEs related to trial procedure
    - The frequency of TEAEs leading to drug withdrawal
    - The frequency of TEAEs leading to study withdrawal
    - The frequency of SAEs
- Physical examination findings, including vital signs (sitting systolic and diastolic blood pressure, heart rate, respiratory rate, body temperature, respiratory rate (breaths/min), resting O_2 saturation).
    - The summary of the general condition in the full physical examination at screening and symptom driven physical examination findings at baseline and post-baseline are performed.
    - The mean change in vital signs (systolic blood pressure (mmHg), diastolic blood pressure (mmHg), heart rate (bpm), temperature (C), respiratory rate (breaths/min) and resting O_2 saturation (%)) from baseline to Week 4
- Change in clinical laboratory assessments, including chemistry, hematology, and urinalysis
    - The mean change in chemistry, hematology, and urinalysis results from baseline to Weeks 4.

Axcella Health Inc./ Cytel Confidential: This is a controlled document and shall not be changed, saved, copied or reproduced by any unauthorized person.

4 ANALYSIS SETS

4.1 Analysis Set Definitions

- **Intent-to-treat analysis set**

The intent-to-treat population (ITT) will consist of all subjects randomized who received at least 1 dose of the study drug. Subjects will be assessed according to their randomized treatment regardless of the treatment they received. The primary population for efficacy analyses is the ITT analysis set.

- **Safety analysis set**

The Safety Analysis Set (SAF) will include all subjects who receive at least 1 dose of study drug. Subjects in the Safety Analysis Set will be analyzed by the treatment received on Day 1. The primary population for safety analyses is the Safety Analysis Set.

- **Per-protocol analysis set**

The per-protocol analysis set (PPS) will include all subjects in the ITT population without any major protocol deviations. Subjects will be analyzed according to their randomized treatment. All analyses performed in the PP analyses set will be considered as sensitivity analyses.

4.2 Protocol Deviations

At the discretion of the sponsor, major protocol deviations as determined by a review of the data prior to final database lock and the conduct of statistical analyses may result in the removal of a subject's data from the PP Analysis Set. The sponsor will be responsible for producing the final protocol deviation file (formatted as a Microsoft Excel file), in collaboration with Cytel as applicable; this file will include a description of the protocol deviation, and clearly identify whether this deviation warrants exclusion from the PP Analysis Set. This file will be finalized prior to final database lock.

All protocol deviations will be presented in the data listings.

Axcella Health Inc./ Cytel Confidential: This is a controlled document and shall not be changed, saved, copied or reproduced by any unauthorized person.

5 DATA HANDLING

5.1 Computing Environment

All statistical analyses will be performed using SAS statistical software (Version 9.4), unless otherwise noted. Medical History and adverse events will be coding using MedDRA version 24.1. Concomitant medications will be coded using World Health Organization (WHO) Drug version Sept 2021G B3.

5.2 Methods of Pooling Data

Not applicable to the present study.

5.3 Withdrawals, Dropouts, Loss to Follow-up

Subjects who discontinue or withdraw from the study will not be replaced.

Subjects may withdraw his or her consent to participate in the study at any point for any reason without prejudice or consequence. Likewise, a subject may also be withdrawn if the Principal Investigator (PI) deems a subject unfit to continue or complete the study or may be discontinued from study drug intake.

5.4 Visit Windows

Analysis visits will be derived with windowing where appropriate (e.g., Visit 4 ± 3 days) per the protocol. Screening can be up to 4 weeks before start of study treatment. Baseline is defined as the most recent assessment prior to randomization. If two or more treatment visits occur within a window, the closest visit to the target day will be used as the analysis visit; if two visits are equidistant from the scheduled analysis visit day, the later analysis visit will be used. Visits outside of the protocol visit windows will be identified, and the allocation will be reviewed prior to database lock. If necessary, analysis visit windows may be adjusted, and the details will be provided in the clinical study report.

Axcella Health Inc./ Cytel Confidential: This is a controlled document and shall not be changed, saved, copied or reproduced by any unauthorized person.

6 STATISTICAL METHODS

6.1 Sample Size Justification

The sample size calculation is based on the primary endpoint, the mean change from baseline at Week 4 in the PCr recovery rate. Based on literature review, the standard deviation (SD) of both the AXA1125 and the placebo group is assumed to be 10 seconds. To detect a clinically meaningful difference of 10-second improvement, approximately 32 subjects will provide 80% power at a 2-sided, 5% significance level. Assuming 20% dropout rate, approximately 40 subjects (20 subjects per arm) will be enrolled in this study.
This sample size will also allow for a general assessment of safety and tolerability of AXA1125 in this population.

6.2 General Statistical Methods

6.2.1 General Methods

All outputs will be incorporated into combined PDF files or Word files, sorted and labeled according to the International Conference on Harmonization (ICH) recommendations, and formatted to the appropriate page size(s).

Tabulations will be produced for appropriate demographic, baseline, efficacy and safety parameters. For categorical variables, summary tabulations of the number and percentage within each category (with a category for missing data) of the parameter will be presented. For continuous variables, the mean, median, standard deviation, minimum and maximum values will be presented.

Statistical analyses will be performed at the 2-sided, 0.05 level of significance. No multiplicity adjustment will be applied. Summary statistics will be presented, as well as confidence intervals on selected parameters, as described in the sections below.

6.2.2 Definition of Baseline

For all variables, Baseline value is defined as the most recent assessment prior to randomization.

6.2.3 Adjustments for Covariates

Baseline value will be adjusted in the analysis of covariance model (ANCOVA) for continuous endpoints.

6.2.4 Multiple Comparisons/Multiplicity

No adjustments for multiplicity will be made.

Axcella Health Inc./ Cytel Confidential: This is a controlled document and shall not be changed, saved, copied or reproduced by any unauthorized person.

6.2.5 Subgroups

No subgroup analysis is planned. Ad-hoc exploratory analyses in selected subgroups may be performed.

6.2.6 Missing, Unused, and Spurious Data

The following rules will be followed to impute fully or partially missing data unless otherwise specified in Sections 6.4, **Error! Reference source not found.** and 6.5.

For binary endpoints, both observed-case and worst-case methods will be applied as the missing imputation method. Worst-case method is defined as subjects with missing data due to any reason will be considered as not meeting the endpoint. Observed-case imputation method is defined as subjects with missing data will not be included in the analysis.

For continuous endpoints, single missing imputation BOCF (baseline observation carried forward) will be applied to analyze the missing data as a sensitivity analysis.

The following imputation rules will apply for partial dates where at least year is available:

- For start dates, missing months and days will be imputed as “01”, provided this occurs on or after the date of first study drug self-administration. Otherwise, the date or month (as appropriate) of the first self-administration of study drug will be used

- For stop dates, missing months will be imputed as “12” and missing days will be imputed as the last day of the month. If this creates a date after discontinuation/completion, the date of discontinuation/completion will be used

The imputed partial dates will only be used to classify events, medications, or therapy as treatment emergent or concomitant. Imputed dates will only be used in the table analyses. Listings will display the available date data.

All data recorded on the CRF will be included in data listings that will accompany the clinical study report.

6.3 Study Population

6.3.1 Subject Disposition

A tabulation of subject disposition will be provided by treatment, and overall, for the ITT, SAF and PPS including the number and percentages of subjects who have:

- Randomized

Axcella Health Inc./ Cytel Confidential: This is a controlled document and shall not be changed, saved, copied or reproduced by any unauthorized person.

- Dosed with any treatment
- Completed study treatments
- Early treatment termination and primary reason for treatment discontinuation
- Completed study (percentages based on the number dosed)
- Early study termination (percentages based on the number dosed) and primary reason for study discontinuation

Separate tabulations will be provided for:

- Screened, screen failed and primary reason for screen failure
- An overview of analysis sets by treatment and overall

A by-subject listing of study completion information will be presented.

6.3.2 Demographic and Baseline Characteristics

Subject demographics and baseline characteristics, including age, sex, race, ethnicity, weight, height, and BMI will be summarized using descriptive statistics and overall, for the ITT, SAF and PPS. No statistical comparison will be performed. Demographic and Baseline data will be provided in data listings.

Medical history will be summarized according to treatment and overall by System Organ Class (SOC) and Preferred Term (PT) for the ITT and SAF. Medical history will be included in a by-subject data listing.

6.3.3 Extent of Exposure

Description of Exposure will be performed on the SAF.

First and Last Dose of treatments will be determined using the Drug Accountability and the End of Treatment page from the CRF. The duration of treatment exposure, calculated as (date of last dose – date of first dose) + 1, will be summarized with descriptive statistics for the SAF. Compliance percentage will be calculated for each subject as the [(number of sachets dispensed - number of sachets returned)/(duration of exposure × 6)] × 100 and summarized with descriptive statistics. Compliance dosing information for each treatment and each subject will be provided in a data listing.

6.4 Efficacy Evaluation

Efficacy analysis will be conducted using the ITT and PPS as outlined below.

6.4.1 Primary Efficacy Analysis

The primary efficacy analysis will be performed using an analysis of covariance (ANCOVA) model with the absolute change from baseline in PCr recovery rate time constant (seconds) following moderate

Axcella Health Inc./ Cytel Confidential: This is a controlled document and shall not be changed, saved, copied or reproduced by any unauthorized person.

exercise as the dependent variable, the randomized treatment group as a factor and the PCr baseline value as a covariate. Least squares mean for each treatment group and for the difference between treatment groups will be presented along with two-sided p-values and 95% confidence intervals. Summary statistics will also be presented for actual values and mean change by treatment group and overall.

6.4.1.1 Sensitivity Analysis

The sensitivity analysis will include repeating the primary analysis on the PPS with observed data and imputing the missing data using single missing imputation BOCF (baseline observation carried forward) on the ITT if applicable.

6.4.2 Secondary/Exploratory Analysis

Mean change from baseline continuous secondary and exploratory endpoints in Section 3.1 will be analyzed with Analysis of Covariance models adjusted for the baseline value. Least squares mean for each treatment group and for the difference between treatment groups will be presented along with two-sided p-values and 95% confidence intervals. Summary statistics will also be presented for observed values and mean (percentage) change by treatment group and overall. The same ANCOVA SAS code used in primary analysis will be applied for the secondary and exploratory endpoints.

Comparisons between AXA1125 versus placebo for secondary and exploratory - binary endpoints in Section 3.1 will be analyzed using a Chi-squared test or Fisher's exact test depending on the frequency of each group. The risk difference with 95% confidence interval (Wald method) will be derived.

6.4.2.1 Sensitivity Analysis

For continuous endpoints, BOCF will be performed to impute the missing data as a sensitivity analysis. For binary endpoint, Worst-Case missing imputation (any missing data imputed to not meeting endpoint) will be used as sensitivity analysis, if applicable.

6.5 Safety Evaluations

Safety analyses will be conducted using the Safety Analysis Set.

6.5.1 Adverse Events

Adverse events will be coded using the Medical Dictionary for Regulatory Activities (MedDRA) and displayed in tables and listings using SOC and PT.

Analyses of adverse events will be performed for those events that are considered treatment emergent, where treatment emergent is defined as any AE which begins or worsens in severity after first dose of study drug is taken.

Axcella Health Inc./ Cytel Confidential: This is a controlled document and shall not be changed, saved, copied or reproduced by any unauthorized person.

Adverse events are summarized by subject incidence rates, therefore, in any tabulation, a subject contributes only once to the count for a given adverse event (SOC or PT), regardless of the number of episodes.

An overview table with the frequencies and percentages of subjects with the following will be provided by treatment and overall:

- Treatment emergent AEs (TEAE), defined as any AE which begins or worsens in severity after the first dose of study drug is taken
- TEAEs by worst CTCAE severity grade (1, 2, 3, 4, or 5)
- TEAEs by worst outcome (fatal, not recovered or not resolved, recovered or resolved, recovered or resolved with sequelae, recovering or resolving, or unknown)
- TEAEs by worst relationship to study treatment (Related vs. Not Related
- TEAEs by worst relationship to trial procedure (Related vs. Not Related)
- TEAEs by worst action taken with study treatment (drug withdrawn, drug interrupted, dose reduced, dose not changed, not applicable, or unknown)
- TEAEs by other action taken (non-drug therapy required, concomitant medication required, other, or none)
- Serious TEAEs (SAEs)

Frequencies and percentages will also be given of subjects with the following by SOC, PT, treatment, and overall:

- TEAEs
- TEAEs by worst CTCAE severity grade
- TEAEs by worst relationship to study treatment
- TEAEs by worst relationship to trial procedure
- TEAEs leading to drug withdrawal
- TEAEs leading to study withdrawal
- SAEs

No formal hypothesis-testing of adverse events incidence rates will be performed.

All adverse events reported during the study will be listed in data listings.

Data listings also will be provided for the following: subject deaths; serious adverse events; adverse events leading to drug withdrawal; and adverse events leading to study withdrawal.

6.5.2 Laboratory Data

Clinical laboratory values will be expressed using International System of Units (SI).

Axcella Health Inc./ Cytel Confidential: This is a controlled document and shall not be changed, saved, copied or reproduced by any unauthorized person.

The actual value and change from baseline to each on study evaluation will be summarized for each clinical laboratory parameter, including hematology, chemistry, and urinalysis, by treatment and overall. In the event of repeat values, the last non-missing value per study day/time will be used.

All laboratory data will be provided in data listings.

6.5.3 Vital Signs and Physical Examinations

The actual value and change from baseline to each on study evaluation will be summarized for vital signs by treatment and overall.

Vital sign measurements will be presented in a data listing.

Physical examination results at each time point will be summarized. All physical examination findings will be presented in a data listing.

6.5.4 Echocardiogram

All echocardiogram data for each subject will be provided in a data listing.

6.5.5 Concomitant Medications

Prior and Concomitant medications will be coded using the WHO Drug dictionary version Sept 2021G B3.

A medication given prior to the first dose of study drug will be classified as a prior medication. A medication given with, or after the first dose of study drug will be classified as concomitant. Prior medications continuing during the study will be classified as prior and concomitant medication and labelled accordingly in the listings. Concomitant medications will be summarized according to treatment and overall, by ATC classification system level 2 (therapeutic main group) and preferred term for the ITT and SAF.

If a medication date or time is missing, or partially missing, and it cannot be determined whether it was taken on or after start of treatment, it will be considered a concomitant medication.

The use of Concomitant medications will be included in by-subject data listing.

Axcella Health Inc./ Cytel Confidential: This is a controlled document and shall not be changed, saved, copied or reproduced by any unauthorized person.

7 CHANGES TO PLANNED ANALYSES

Besides of Bimodal scoring, CFQ-11 total score in Likert scoring will be also calculated. The two subscales such as physical and mental scores will be also calculated in both Bimodal and Likert scoring. The more detail is described in Section 3.1 page 13.

Axcella Health Inc./ Cytel Confidential: This is a controlled document and shall not be changed, saved, copied or reproduced by any unauthorized person.

8 REFERENCES

1. International Council on Harmonization, Statistical Principles for Clinical Trials (ICH E9)

2. Enright PL, Sherrill DL. Reference equations for the six-minute walk in healthy adults. American Journal of Respiratory and Critical Care Medicine.1998;158:1384-87

Axcella Health Inc./ Cytel Confidential: This is a controlled document and shall not be changed, saved, copied or reproduced by any unauthorized person.

9 APPENDICES

9.1 Schedule of Assessments

	Screening Visit 1	Baseline Visit 2	Treatment Period Visit 3 (Phone call)	EOT or ER Visit 4	Follow-up Visit 5 (Phone Call)
Day	-28 to -1	1	14	28	35
Week	-4 to-1	0	2	4	5
Window			±3	±3	±3
Informed consent	X				
Demographics	X				
Medical, surgical, and medication history	X				
Height, weight, and BMI	X				
Vital signs (BP, HR, RR, body temperature, and resting O2[a])	X	X		X	
Echocardiogram[b]	X				
Prior and/or concomitant medication	X	X		X	
Physical examination[c]	X	X	X	X	X
Magnetic resonance spectroscopy[d] (^{31}P-MRS and ^{1}H-MRS)	X			X	
Blood and urine collection for local laboratory Assessments[e]	X	X		X	
Serum NT-pro BNP[f]	X			X	
Alcohol breath test	X	X			
Blood/plasma collection for central laboratory assessments[g,h]		X		X	
Pregnancy testing (females with reproductive potential)[i]	X	X		X	
6-Minute Walk Test[j]		X		X	
Serum lactate[k]		X			
CFQ-11 PRO for fatigue[l]	X	X	X	X	
Treatment assignment		X			
Study drug dispensing		X			
Study drug self-administration[m]		X	X	X	
Study drug compliance review			X[n]	X	
AE assessment	X	X	X	X	X

6MWT=6-minute walk test; AA=amino acid; AE=adverse event; BMI=body mass index; BNP=B-type natriuretic peptide; BP=blood pressure; CFQ=Chalder Fatigue Questionnaire; CHF=congestive heart failure; ECAR=extracellular acidification rate; EOT=End of Treatment; ET=early termination; FGF-21=fibroblast growth factor-21; ^{1}H-MRS=proton magnetic resonance spectroscopy; HR=heart rate; LV=left ventricle; LVEF=left ventricular ejection fraction; MRS=magnetic resonance spectroscopy; NO=nitric oxide; OCR=oxygen consumption rate; PE=physical examination; PK=pharmacokinetics; ^{31}P-MRS=phosphorus magnetic resonance spectroscopy; PRO=patient-reported outcomes; RR=respiration rate; SRM=Study Reference Manual.

[a] To be performed prior to blood collection. Resting O_2 saturation to rule out significant pulmonary disease as a cause of fatigue (must be ≥95% on room air)

[b] To rule out significant tricuspid regurgitation, evidence of pulmonary hypertension, or any significant decrease in LVEF or LV wall abnormality consistent with CHF, unless
documentation of a normal cardiac echocardiogram within 3 months of Screening and after at least 3 months of acute COVID-19 infection. Note: An echocardiogram is not
required to be performed at Screening if documentation of a normal or not clinically significant echocardiogram was performed within 3 months of Screening and after at least

Client / Cytel Confidential: This is a controlled document and shall not be changed, saved, copied or reproduced by any unauthorized person.

3 months of acute COVID-19 infection.
[c] A full physical examination is performed at Screening to ensure eligibility. At Visits 2 and 4, a targeted symptom-driven examination can occur (eg, lungs, heart, or based on occurrence of symptoms or AEs).
[d] Documented ^{31}P-MRS within 3 months of Screening and after 3 months acute COVID-19 infection is acceptable. If a historical ^{31}P-MRS has been used to determine eligibility, then ^{31}P-MRS should be repeated within 1 month prior to Day 1 (ie, during the Screening Period) to confirm eligibility and establish baseline. Subjects are required to fast (water permitted) for a minimum of 6 hours prior to the MRS.
[e] Complete blood count, chemistry, and routine urine analysis
[f] To rule out significant CHF as a cause of fatigue (must be <400 pg/mL)
[g] Change in circulating mitochondrial peptides (eg, Mots-C), metabolomics, proteomics, inflammation biomarkers, adhesion markers, muscle injury biomarkers (eg, troponins, creatine kinase, FGF-21), and mitochondrial function/metabolism markers in plasma (~1.5 mL total); NO biology; immune profiling, and metabolism/phenotypic ECAR/OCR; plasma AA profiling; and metabolomics. Plasma AA (PK samples) are to be collected no more than 30 minutes before administration of study drug and after a fast (minimum duration) of 10 hours.
[h] Plasma metabolomic assays will be collected prior to and following moderate exercise.
[i] A serum pregnancy test will be performed at Screening. A urine pregnancy test will be performed at Baseline and EOT/ET (Visit 4).
[j] 6MWT should be performed at (or within 7 days of) Baseline and EOT/ET (Visit 4). The subject may have clinic visit assessments performed over 2 days. The subject will be provided an overnight stay at a nearby hotel.
[k] A capillary blood lactate test by earlobe puncture is measured just before starting, immediately after and 5, 10, 20, and 30 minutes after the 6MWT.
[l] At all visits other than Screening (Visit 1), it is to be given both before and after 6MWT.
[m] The study drug should be self-administered at the clinic visit on Day 1 and Day 28 or EOT/ET.
[n] At Visits 3 and 4, a compliance review will be performed.

Client / Cytel Confidential: This is a controlled document and shall not be changed, saved, copied or reproduced by any unauthorized person.
