# Supplementary material for: Efficacy and tolerability of an endogenous metabolic modulator (AXA1125) in fatigue-predominant long COVID: a single-centre, double-blind, randomised controlled phase 2a pilot study
Source: eClinicalMedicine. 2023 Apr 14;59:101946. doi: 10.1016/j.eclinm.2023.101946 (PMC10102537; doi:10.1016/j.eclinm.2023.101946)
Supplement: Caption for supplementary material [file mmc4.docx]

**Caption for supplementary material**

Post hoc analysis

Introduction

Methods

Results

Discussion

Supplement Table 1. Summary of fatigue responder analysis findings

Supplement Figure 1. Box plots of individual changes from baseline to Day 28 in phosphocreatine recovery time constant in (a) AXA1125 versus placebo-treated patients, (b) AXA1125 responders versus non-responders, and (c) placebo responders versus non-responders.

Supplement Figure 2. Box plots of individual changes from baseline to Day 28 in total distance covered during 6-minute walk test in (a) AXA1125 versus placebo-treated patients, (b) AXA1125 ‘responders’ versus ‘non-responders’, and (c) placebo ‘responders’ versus ‘non-responders’.
